# Influenza A virus subverts the LC3-pericentrin dynein adaptor complex for host cytoplasm entry

**DOI:** 10.1126/sciadv.adu7602

**Published:** 2025-06-11

**Authors:** Yingying Cong, Pauline Verlhac, Benjamin B. Green, Jacqueline de Vries-Idema, Line Moesgaard Strauss, Clàudia Río-Bergé, Anders Etzerodt, Lene N. Nejsum, Anke L. W. Huckriede, Fulvio Reggiori

**Affiliations:** ^1^ Department of Biomedicine, Aarhus University, Aarhus, Denmark.; ^2^ Department of Drug Design, University of Groningen, Groningen, Netherlands.; ^3^ Department of Biomedical Sciences, University of Groningen, University Medical Center Groningen (UMCG), University of Groningen, Groningen, Netherlands.; ^4^ Department of Clinical Medicine, Aarhus University, Aarhus, Denmark.; ^5^ Department of Medical Microbiology and Infection Prevention, UMCG, University of Groningen, Groningen, Netherlands.; ^6^ Aarhus Institute of Advanced Studies (AIAS), Aarhus University, Aarhus, Denmark.

## Abstract

Influenza A virus (IAV) enters host cells via endocytosis, and fusion of the viral particles (VPs) at endosomes releases the viral ribonucleoproteins (vRNPs) into the cytoplasm. This uncoating step that is vital for IAV infection remains to be fully understood. The aggresome processing machinery (APM) plays a relevant but not essential role in this. Here, we reveal a mechanism in which light chain 3 proteins (LC3s) and pericentrin (PCNT) form an adaptor complex that is required for vRNPs binding to the dynein 1 and IAV uncoating at endosomes. This function of LC3s and PCNT is independent from their established role in autophagy and centrosome assembly, respectively. LC3s or PCNT depletion severely impairs IAV cytoplasm entry and infection, which can be further inhibited by additional silencing of histone deacetylase 6, an APM component. Collectively, our results show that IAV has adopted two redundant strategies to hijack the dynein biomolecular motors and facilitate VP uncoating.

## Teaser

IAV exploits LC3-PCNT and HDAC6 pathways to engage dynein motors for uncoating and entry into the host cell cytoplasm.

## INTRODUCTION

Influenza A virus (IAV) is one of the principal respiratory human pathogens. IAV is an enveloped virus with a segmented genome consisting of single-stranded negative-sense RNA that encode 10 proteins: two nonstructural proteins, i.e., NS1 and NS2; and eight structural proteins that compose the viral particles (VPs), i.e., polymerase basic protein 2 (PB2), polymerase basic protein 1 (PB1), polymerase acidic protein (PA), hemagglutinin (HA), nucleoprotein (NP), neuraminidase (NA), matrix protein 1 (M1), and matrix protein 2 (M2) (*1*). NP is the most abundantly synthesized structural protein and has multiple functions, including mediating the transcription, replication, and encapsidation of the viral genome. The viral genomic RNA is wrapped around an oligomeric NP filament that is bound to a single copy of the viral polymerase consisting of PB1, PB2, and PA, forming viral ribonucleoproteins (vRNPs) that bundle together into a stable, capsid-like complex with the matrix protein M1 (*1*). IAV VPs enter cells through endocytosis and release their genome in the host cytoplasm by fusing with the membrane of late endosomes (LEs), leading to both the release of vRNPs into the cytoplasm and the dissociation of M1 proteins, which disperse into the cytosol (*2*). Cytoplasmic vRNPs are then imported into the nucleus, where IAV transcription and replication take place.

The aggresome processing machinery (APM) is an intracellular system in which unanchored ubiquitin chains on aggregates bind and activate the histone deacetylase 6 (HDAC6), which, in turn, stimulates actin- and myosin-dependent aggresome formation and disassembly (*3*). IAV vRNPs carry unanchored ubiquitin chains that are recognized by HDAC6 at the viral fusion sites at LEs (*4*–*6*). HDAC6 interacts with both the dynein 1 and myosin II, and this allows IAV to exploit the physical forces generated by microtubule (MT)– and actin-associated biomolecular motors for uncoating and, thus, cytoplasm entry (*4*). In agreement with these findings, a small interfering RNA (siRNA)–based genome-wide screen has shown that the dynein 1 complex subunit dynein cytoplasmic 1 intermediate chain 1 (DYNC1I1) is important for IAV infection (*7*). In addition, transportin-1 (TNPO1), a receptor for the nuclear import of cellular ribonucleoproteins and RNA binding proteins, completes the release of the IAV genetic material into host cells, as it promotes the removal of residual M1 inducing the disassembly of the vRNP bundles (*8*). The epidermal growth factor receptor pathway substrate 8 (EPS8), ISG lymphocyte antigen 6 complex, locus E (LY6E), and itchy E3 ubiquitin protein ligase (ITCH) also promote IAV uncoating (*9*–*11*), but it remains unclear what role these proteins fulfill. However, depletion of these proteins only partially impairs the IAV infection (*4*, *8*–*11*), suggesting that IAV may have additional mechanisms to effectively release its genetic material into host cells.

Autophagy is a degradative pathway mediated by the autophagy-related (ATG) proteins (*12*). One of the conserved functional ATG protein clusters is the ubiquitin-like conjugation system that, through the action of the ATG7 E1-, ATG3 E2-, and ATG12-ATG5-ATG16L1 E3-like enzymes, leads to the covalent linkage of the members of the ATG8 protein family mainly to phosphatidylethanolamine (PE) (*12*). The C terminus of ATG8 proteins is constitutively posttranslationally processed by ATG4 proteases to expose a Gly, generating their so-called ATG8-I forms, which are then conjugated to amino group of PE leading to the biosynthesis of the lipidated ATG8-II forms (*12*). The six human ATG8 proteins are divided into MT-associated protein 1 light chain 3 (LC3) and γ-aminobutyric acid receptor–associated protein (GABARAP) subfamilies, i.e., LC3s and GABARAPs. These subfamilies include LC3A, LC3B, and LC3C and GABARAP, GABARAPL1, and GABARAPL2, respectively. The lipidated ATG8 proteins have partially redundant functions in autophagosome expansion, closure, and fusion with lysosomes as well as the degradation of their internal lipid bilayer and the selective sequestration of distinct cargo by autophagosomes (*13*). Moreover, LC3s on the surface of autophagosomes can associate with biomolecular motors from the dynein and kinesin protein families, regulating the intracellular distribution of autophagosomes (*12*, *14*). ATG8 proteins can also be conjugated to PE present in the membrane of organelles such as endosomes, where they promote maturation, signaling, and fusion (*15*). Specific cellular functions have also been linked to the ATG8-I forms. Nonlipidated LC3s are involved in a membrane transport route out of endoplasmic reticulum (ER) known as the ER-associated degradation (ERAD) tuning pathway (*16*), which appears to be hijacked by the mouse hepatitis virus (MHV), the equine viral arteritis (EAV), and Japanese encephalitis virus (JEV), to generate their replication platforms (*17*–*19*). Nonlipidated LC3s are also associated with *Chlamydia trachomatis* inclusions, an essential for the intracellular growth of this bacterium (*20*). Last, coxsackievirus B3 (CVB3) can exploit both LC3B-I– and LC3B-II–coated membranes to promote its replication (*21*).

In this study, we uncovered a key role of LC3s and pericinetrin (PCNT) in IAV infection. Specifically, we revealed that LC3s and PCNT form an adaptor complex that is required for vRNPs binding to the dynein 1 biomolecular motors and IAV VP uncoating at LEs. We also show that this mechanism of IAV VP uncoating is redundant with the one involving the HDAC6-dependent APM. Our results suggest that IAV has developed two different strategies to subvert the dynein biomolecular motors to facilitate its host cytoplasm entry.

## RESULTS

### HDAC6 is not the only dynein adaptor protein that promotes IAV cytoplasm entry

**Fig. 1.**
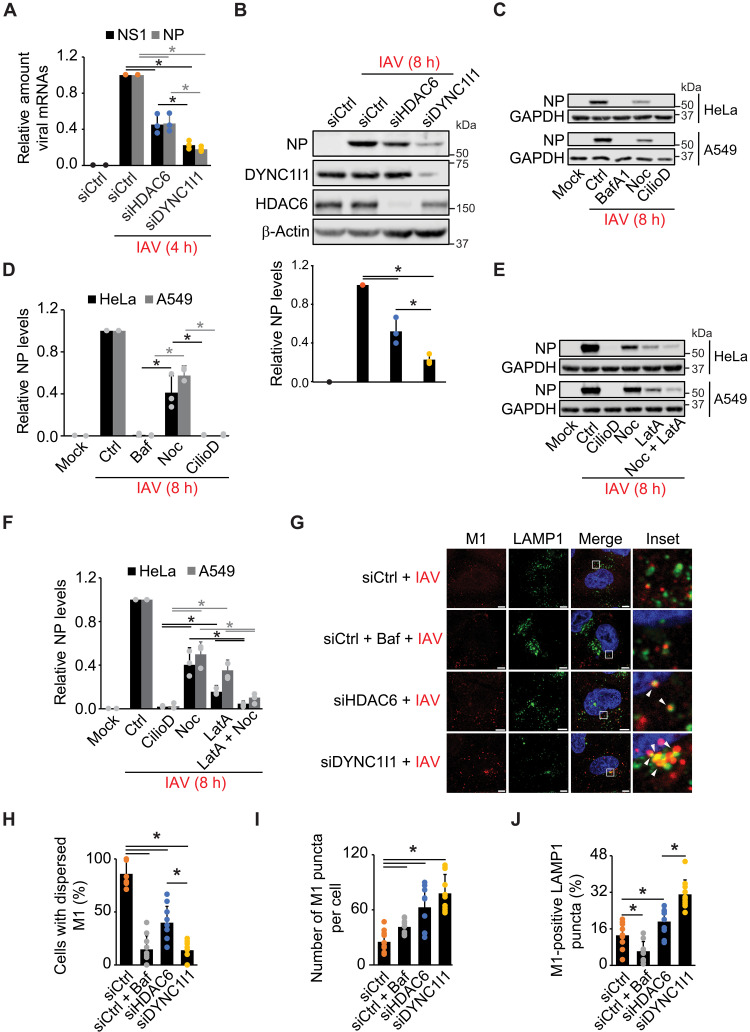
HDAC6 is not the only dynein adaptor promoting IAV cytoplasm entry. (**A**) HDAC6- or DYNC1I1-depleted A549 cells were infected with IAV at a multiplicity of infection (MOI) of 0.1 for 4 hours (h). NS1 and NP mRNA levels were quantified by RT-PCR, normalized to glyceraldehyde-3-phosphate dehydrogenase (GAPDH), and expressed relative to siCtrl cells. (**B**) Cells in (A) were infected for 8 hours before WB with anti HDAC6, DYNC1I1, NP, and β-actin antibodies. NP levels were quantified relative to siCtrl cells. (**C**) sHeLa and A549 cells were infected with IAV at an MOI of 0.1 for 8 hours in the presence of 200 nM Baf, 1 μM Noc, or 10 μM CilioD before WB analysis using anti-NP and anti-GAPDH antibodies. (**D**) NP levels in (C), relative to untreated cells (Ctrl). (**E**) sHeLa and A549 cells were infected with IAV at an MOI of 0.1 for 8 hours in the presence of 10 μM CilioD, 1 μM Noc, 0.1 μM LatA, or 1 μM Noc and 0.1 μM LatA simultaneously before WB analysis with anti-NP and anti-GAPDH antibodies. (**F**) NP levels in (E), relative to untreated cells (Ctrl). (**G**) The cytoplasm entry assay was carried out in siCtrl-, siHDAC6-, or siDYNC1I1-treated sHeLa cells, which were then processed for IF with anti-M1 and anti-LAMP1 antibodies. Baf (200 nM) was used under the siCtrl condition to block IAV cytoplasm entry. Insets highlight M1-positive LAMP1 puncta. Images were acquired using a ZEISS LSM800 microscope. Scale bars, 5 μm. (**H** to **J**) Quantification of the cell percentage with dispersed M1 (H), the number of M1 puncta per cell (I), and the percentage of M1-positive LAMP1 puncta (J) in (G). Error bars represent SDs [*n* = 3 in (A), (B), (D), and (F); *n* = 10 in (H) to (J), 50 cells counted per repeat]. Asterisks indicate significant differences.

It has been shown that IAV can subvert the host cell’s APM to promote VP uncoating at LEs (*4*). However, depletion of HDAC6, an APM component, only reduced IAV infection by approximately half, suggesting that IAV takes advantage of other host factors for cytoplasm entry. We reexamined HDAC6 relevance in the IAV viral cycle by depleting this protein in A549 cells using siRNA before assessing IAV infection by measuring viral NS1 and NP gene transcription at 4 hours postinfection (hpi) by reverse transcription polymerase chain reaction (RT-PCR) and NP protein levels at 8 hpi by Western blot (WB) (Fig. 1, A and B). We also knocked down DYNC1I1, a subunit of the dynein 1 biomolecular motor that is involved in HDAC6-mediated IAV uncoating (*4*). While DYNC1I1 knockdown almost completely blocked IAV replication, HDAC6 depletion decreased IAV infection by only approximately half (Fig. 1, A and B), as reported (*4*). These results were confirmed in sHeLa cells (fig. S1, A to C) and suggested that dynein 1 plays a more relevant role than HDAC6 in the IAV viral cycle.

The major relevance of dynein 1 in IAV replication may be linked to MT function in other steps of the IAV viral cycle. Thus, we evaluated the role of MTs in IAV infection by depolymerizing them with nocodazole (Noc) and assessing viral NP expression at 8 hpi in both A549 and sHeLa cells. Cells were treated with ciliobrevin D (CilioD), a specific inhibitor of the cytoplasmic dynein–based biomolecular motors, or bafilomycin A1 (Baf), a vacuolar H^+^-dependent adenosine triphosphatase (H^+^-ATPase) inhibitor, which reduces IAV cytoplasm entry. Noc reduced replication by only 50 to 60%, compared to nearly 90% reduction with CilioD and Baf (Fig. 1, C and D). This result indicated that dyneins are essential for IAV infection while MTs are only partly involved in this process. As dynein-based biomolecular motors not only associate with MTs but also cooperate with actin filaments for transport (*22*), we depolymerized actin filaments with latrunculin A (LatA), concomitantly or not with Noc treatment, and consistently with previous data (*4*), simultaneous treatment with Noc and LatA inhibited viral NP expression more severely than Noc or LatA individually, to an extent similar to CilioD treatment or DYNC1I1 knockdown (Fig. 1, E and F). We also examined the infection of luciferase-expressing herpes simplex virus (luc-HSV-1) and vaccinia virus (luc-VaV) in cells treated in the same way. While luc-VaV infection was minimally altered by CilioD or Noc, luc-HSV-1 infection was pronouncedly inhibited by Noc alone or in combination with LatA (fig. S1D). These data showed that dyneins and cytoskeleton are not essential for all infections, inferring for a specific role in the IAV viral cycle.

To determine whether the critical function of dyneins in the IAV viral cycle is at the cytoplasm entry, we used an established immunofluorescence (IF)–based cytoplasm entry assay that allows monitoring the uncoating of VPs at endosomes (*4*) (see Materials and Methods) in DYNC1I1- and HDAC6-depleted sHeLa cells. To determine in which endocytic organelle VPs were trapped in the absence of HDAC6 or DYNC1I1, we also colocalized the viral M1 protein with sorting nexin 1 (SNX1) and lysosomal-associated membrane protein 1 (LAMP1), an early endosome and an LE/lysosome marker protein, respectively (*23*). M1 was distributed in puncta and dispersed in the cytoplasm in the cells treated with scramble siRNA (siCtrl) (Fig. 1, G and H, and fig. S1E), as expected (*4*). In contrast, M1 was almost exclusively detected in puncta in Baf-treated or DYNC1I1-depleted cells, while HDAC6 silencing only partially reduced IAV cytoplasm entry. Quantification of the average number of M1-positive puncta per cell also showed that M1 puncta accumulated under all conditions in comparison to the siCtrl control (Fig. 1I). While it rarely colocalized with SNX1 in all the samples (fig. S1F), M1 was more markedly present in LAMP1-positive compartments especially in the siHDAC6 or siDYNC1I1 knockdown than in control cells (Fig. 1J), in agreement with the notion that the cytoplasmic release of vRNPs takes place at LEs (*4*, *24*). Notably, DYNC1I1 silencing led to a more elevated colocalization between M1 and LAMP1 than HDAC6 depletion, confirming that dynein 1 plays a more critical role than HDAC6 during IAV cytoplasm entry.

### LC3s but not the entire ATG machinery are important for IAV infection

Dyneins have crucial roles in numerous cellular processes, including autophagy, in which, together with MTs, they are responsible for the transport and localization of autophagosomes (*25*). Specific adaptor proteins and LC3s are key to anchoring autophagosomes to dyneins (*14*). LC3B interacts with NP and M2 proteins from an avian IAV strain (*26*). Therefore, we explored whether ATG8 proteins play a role in IAV uncoating. We took advantage of sHeLa cells that lack the three members of the LC3 or the GABARAP protein subfamily, i.e., LC3s triple knockout (LC3TKO) and GABARAPs triple knockout (GABARAPTKO), respectively (fig. S2A) (*27*). Viral replication was assessed in LC3TKO, GABATKO, and their parental sHeLa cell line by measuring the NP levels at 8, 16, and 24 hpi. NP levels were pronouncedly reduced at each hour postinfection in the LC3TKO but not in parental and GABATKO cells (fig. S2B). Quantification of the percentage of infected cells by IF and viral egression by titration confirmed that IAV infection is inhibited by the absence of LC3s but not GABARAP proteins (fig. S2, C to E). The simultaneous silencing of the three LC3 isoforms in A549 cells using siRNA also resulted in a decrease in 4viral NP expression at 16 hpi, showing that LC3s relevance in IAV infection is not cell type specific (fig. S2F).

Next, we explored whether the IAV viral cycle requires a specific LC3 isoform. LC3TKO cells were individually or simultaneously transfected with plasmids expressing LC3A, LC3B, or LC3C before IAV infection for 16 hours. The NP level analysis revealed that each of the LC3 isoforms could complement the IAV infection defect of LC3TKO cells (fig. S2G), indicating that these proteins have a redundant function in the cellular pathway critical for the IAV viral cycle.

**Fig. 2.**
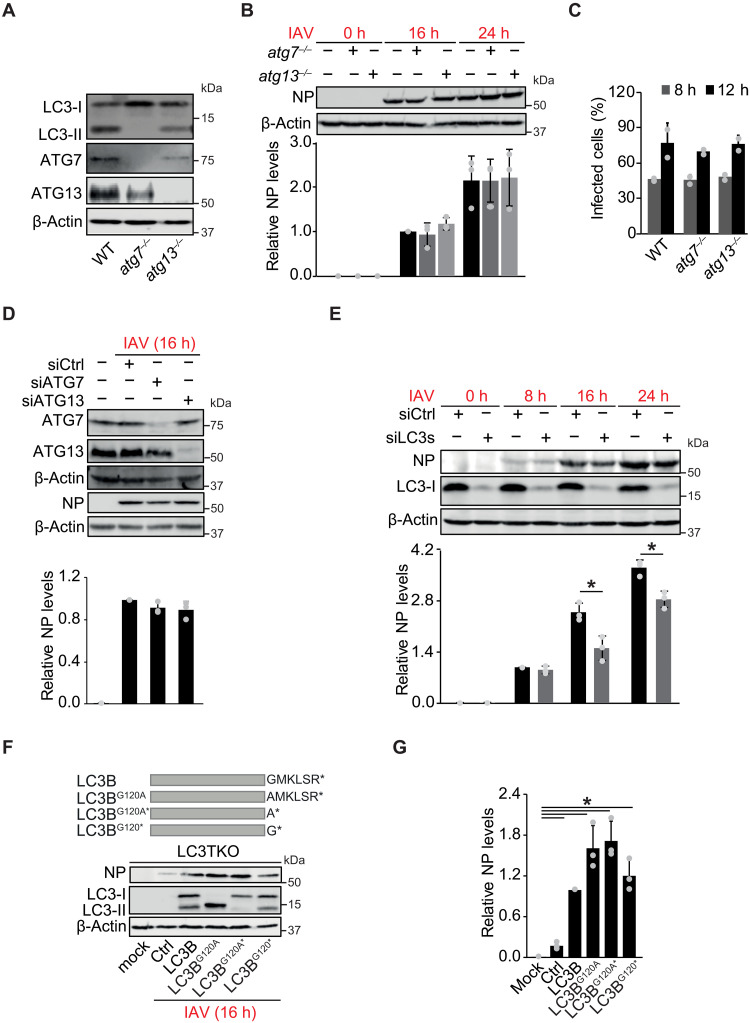
LC3-I proteins but not an intact ATG machinery are required for IAV infection. (**A**) Cell extracts from WT, *atg7^−/−^*, and *atg13^−/−^* U2OS cells were analyzed by WB with anti ATG7, ATG13, LC3, and β-actin antibodies. (**B**) Cells from (A) were infected with IAV at an MOI of 0.1 for 0, 16 and 24 hours before WB examination with antibodies against NP and β-actin (top). NP levels were quantified relative to WT cells infected for 16 hours (bottom). (**C**) Cells from (A) were infected with IAV at an MOI of 0.1 before being IF with the anti-NP antibody at 8 or 12 hpi to determine the percentage of infected cells using a TissueFAXS microscope. (**D**) ATG7- or ATG13-depleted sHeLa cells were infected with IAV at an MOI of 0.1 for 16 hours and analyzed by WB with anti-NP, ATG7, ATG13, and β-actin antibodies (top). NP levels were quantified relative to siCtrl cells (bottom). (**E**) The *atg7^−/−^* cells were transfected with siCtrl or a pool of siRNA targeting the three members of the LC3 subfamily (siLC3s) before infection with IAV at an MOI of 0.1. Cells extracts were collected at 0, 8, 16, and 24 hpi and examined by WB with anti-NP, LC3, and β-actin antibodies (top). NP levels were quantified relative to siCtrl cells infected for 8 hours (bottom). (**F**) LC3TKO cells were transfected with pcDNA3.1, pCDN3.1-LC3B, pCDN3.1-LC3B^G120A^, pCDN3.1-LC3B^G120A*^, or pCDN3.1-LC3B^G120*^ for 48 hours and then infected with IAV at an MOI of 0.1 for 16 hours before WB with anti-NP, LC3, and β-actin antibodies. (**G**) NP level quantification in (F). Bars represent amounts relative to infected LC3TKO cells transfected with the pCDN3.1-LC3B plasmid. Error bars represent SDs [*n* = 3 in (B), (D), (E), and (G); *n* = 2 in (C)]. Asterisks indicate significant differences. h, hours.

We then investigated whether other key ATG proteins are critical for IAV infection by using two U2OS cell lines (Fig. 2A), one lacking ATG13, a subunit of the unc-51-like kinase (ULK) complex that regulates autophagy initiation (*28*), and the other deficient of E1-like enzyme ATG7, which is essential for the conjugation of LC3s with PE (*29*). Parental, *atg7^−/−^*, and *atg13^−/−^* cells showed no significant differences in the percentage of infected cells at 8 and 12 hpi and the NP levels at 16 and 24 hpi upon exposure to IAV (Fig. 2, B and C). To rule out differences between cell lines, we also examined IAV infection in sHeLa cells depleted of ATG13 or ATG7 and detected no differences in NP levels at 16 hpi (Fig. 2D). Unlike canonical autophagy, conjugation of ATG8s to single membranes (CASM) targets ATG8s to preexisting single-membrane compartments (*30*). To exclude CASM, we repeated IAV infection in ATG16L1-deficient cells, a key component recruited to the V-ATPase proton pump during CASM (*30*), and observed no significant differences in NP levels at 16 and 24 hpi (fig. S2H). To confirm that LC3s have a noncanonical function in the IAV viral cycle, we knocked down the three LC3 isoforms in *atg7^−/−^* cells by siRNA and found that lower LC3 levels resulted in a decrease in NP synthesis in *atg7^−/−^* cells at 16 hpi (Fig. 2E), indicating that LC3 role in IAV infection is both autophagy and CASM independent.

The finding that ATG7 is dispensable for IAV infection suggested that the function of LC3s in the IAV viral cycle does not require their conjugation to PE, a prerequisite for autophagy (*12*). We created variants of LC3B to determine whether LC3 conjugation to PE is unnecessary for IAV infection (Fig. 2F). In the first variant, the critical Gly recognized and exposed by ATG4 proteases was mutated into an Ala, creating a protein that mimics precursor LC3B (LC3B^G120A^). In the second, which mimics LC3B-I, the amino acids removed by ATG4 proteases were deleted, and the exposed C-terminal Gly was changed into an Ala to block its conjugation to PE (LC3B^G120A*^). Last, the last variant was the one mimicking LC3B-I (LC3B^G120*^). LC3TKO cells were then individually transfected with one of these variants or wild-type (WT) LC3B, before assessing NP levels at 16 hpi. All the LC3B variants were able to reestablish IAV infection susceptibility in LC3TKO cells to the same extent as WT LC3B (Fig. 2, F and G).

### LC3s facilitate IAV uncoating from LAMP1-positive LEs

**Fig. 3.**
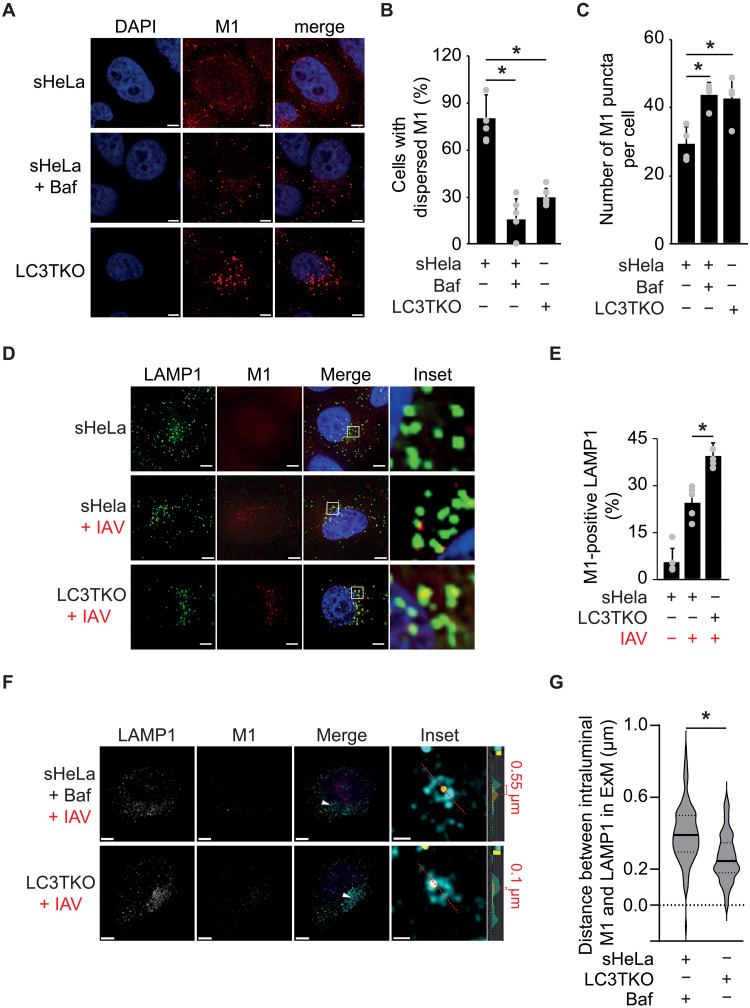
IAV cytoplasm entry requires LC3s. (**A**) IAV cytoplasm entry in sHeLa and LC3TKO cells was assayed as in Fig. 1G. Cells were processed for IF with anti-M1 antibodies. Images were acquired using a ZEISS LSM800 microscope. Scale bars, 5 μm. (**B** and **C**) Quantification of the percentage of cells with dispersed M1 (B) and the number of M1 puncta per cell (C) in (A). (**D**) IAV cytoplasm entry in sHeLa and LC3TKO cells was assayed as in Fig. 1G. Cells were then processed for IF with anti-M1 and anti-LAMP1 antibodies. Insets highlight M1-positive LAMP1 puncta. Images were acquired using a DeltaVision microscope. Scale bars, 5 μm. (**E**) Quantification of M1-positive LAMP1 puncta percentage in (D). (**F**) sHeLa and LC3TKO cells were transfected with siCtrl before infection with IAV as in Fig. 1G and processing for ExM with antibodies against M1 and LAMP1. Baf (200 nM) was used during the IAV cytoplasm entry to block IAV fusion at LEs in sHeLa cells. Cells were expanded ~4.5×, and maximum projection images of individual LAMP1 and M1 signals are displayed in grayscale. In the merged images, LAMP1 is shown in cyan, M1 in yellow, and nuclei in magenta. White arrows highlight the selected area used to draw the red line scans (insets) and measure the signal intensity. The resulting intensity plots were used to determine the distance between M1 and LAMP1 signals. Scale bars, ~4.5 μm (maximum projections) and ~0.2 μm (insets). (**G**) Quantification of the distance of luminal M1 puncta from the LAMP1-positive membrane in single slices of expanded cells shown in (F). Error bars represent SDs [*n* = 4 in (B), (C), and (E), 50 cells counted per repeat; *n* = 3 in (G)]. Asterisks indicate significant differences.

Next, we investigated whether nonlipidated LC3s are involved in the same step of the IAV viral cycle as HDAC6 and dyneins. Using the cytoplasm entry assay, we observed that M1 protein was almost exclusively detected as puncta in LC3TKO cells as in Baf-treated sHeLa cells (Fig. 3, A and B). The number of M1 puncta was also higher in comparison to the control (Fig. 3C), supporting the notion that LC3s are required for IAV cytoplasm entry. To determine whether IAV VPs become entrapped within LEs in LC3TKO cells, we examined the colocalization between M1 and LAMP1 or SNX1 in LC3TKO cells at 3 hpi. M1-positive puncta colocalized predominantly with LAMP1 and not with SNX1 in LC3TKO cells (Fig. 3, D and E, and fig. S3, A and B). This analysis, which also showed a markedly higher colocalization of M1 with LAMP1 in the LC3TKO in comparison to sHeLa cells (Fig. 3E), showed that IAV uncoating requires LC3s. We also examined whether LC3s are possibly involved in other early steps of the IAV viral cycle. We first assessed the binding of IAV VPs to the cell surface that is required for their cell internalization using a standard RT-PCR–based method (*31*). Treatment with neuramidase was used as a positive control since this enzyme removes the sialic acids from the glycans appended to the cell surface proteins and required for IAV attachment. We did not observed differences between the WT and LC3TKO cells (fig. S3C). We checked the virion internalization by endocytosis. For this, we performed the IAV cell entry assay as Fig. 1G but in the present of Baf, to inhibit the cytoplasm entry and thus be able to estimate the quantity of endocytoses IAV trapped in endosomes by IF. We detected no significant differences between the control and LC3TKO cells (fig. S3, D and E).

To obtain insights into the IAV cytoplasm entry defect upon ablation of LC3s, we repeated the cytoplasm entry assay but examined the samples by expansion microscopy (ExM) to resolve the distribution of IAV VPs within LEs (*32*). The ExM procedure led to an expansion of approximately 4.5 times in each dimension (*xyz*) and a concomitant distinct visualization of expanded LAMP1-positive LEs that contained in several instances one or more M1 puncta (Fig. 3F). In contrast to Baf-treated sHeLa cells, luminal VPs appeared more associated with the limiting membrane of LEs in LC3TKO cells. We quantified the average distance between luminal M1 puncta and the LAMP1-positive membrane and found that in the expanded samples, this distance was 265 ± 132 nm in LC3TKO cells and 398 ± 159 nm in Baf-treated cells (Fig. 3G). This result reinforced the notion that LC3s are important for IAV uncoating at LEs.

Last, to exclude that the absence of LC3s could alter general endosomal functions, we infected the LC3TKO cells with luc-HSV-1 and luc-VaV, which enter host cells in a low endosomal pH-dependent manner (*33*, *34*). No significant differences in HSV-1 and VaV infection were observed between sHeLa and LC3TKO cells (fig. S3F). In contrast, treatment of sHeLa cells with Baf significantly impaired HSV-1 and VaV infection as expected. This result indicated that a general endosomal defect is not what causes IAV uncoating impairment in LC3TKO cells.

### LC3s interact with IAV vRNPs and dynein 1 during IAV cytoplasm entry

**Fig. 4.**
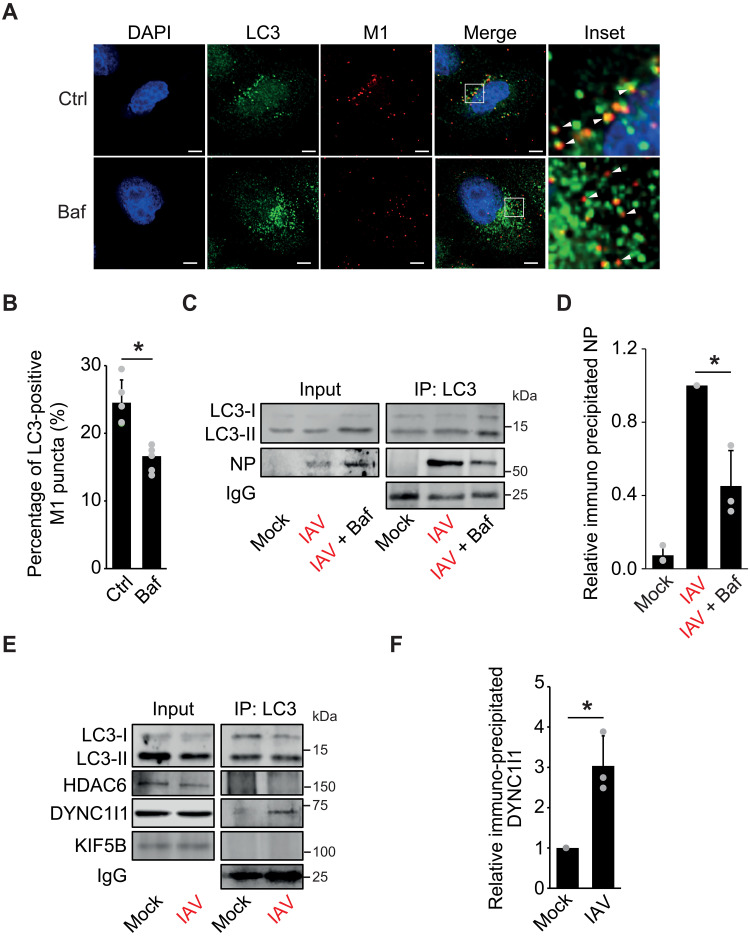
Cytoplasm entry of IAV is modulated by LC3s and dynein 1. (**A**) sHeLa cells treated as in Fig. 1G in the presence or absence of Baf were processed for IF with anti-M1 and anti-LC3 antibodies. Insets highlight the colocalization between LC3 and M1, and white arrowheads point to colocalization (in untreated cells) and noncolocalization (in Baf-treated cells). Images were collected using a DeltaVision microscope. Scale bars, 5 μm. (**B**) Quantification of the LC3-positive M1 puncta in (A). Error bars represent SDs. (**C**) IAV cytoplasm entry in sHeLa cells was carried out as in Fig. 1G, except that IAV was at an MOI of 30. Baf (200 nM) was used to block IAV fusion at LEs. Cell extracts were subjected to IP with LC3 antibodies before separating the coisolated proteins and WB analysis with anti-LC3, NP, and immunoglobulin G (ΙgG) (control) antibodies. (**D**) Quantification of the NP bound to LC3 in (C), expressed relative to infected cells not treated with Baf. (**E**) Cell extracts from sHeLa cells treated and processed as in (C). WB membranes were probed with anti-LC3, HDAC6, DYNC1I1, KIF5B, and ΙgG antibodies (control). (**F**) Quantification of the DYNC1I1 bound to LC3s in (E), expressed relative to mock-treated cells. Error bars represent SDs [*n* = 3 in (D) and (F); *n* = 3 in (B), 50 cells counted per repeat]. Asterisks indicate significant differences.

We next investigated whether LC3s associate with VPs at LEs. The IAV cytoplasm entry assay was repeated in the presence or absence of Baf before examining M1 and LC3 distribution by IF. Part of the LC3 puncta colocalized with M1 in infected sHeLa cells not incubated with Baf. In contrast, treatment with Baf significantly reduced the colocalization between LC3s and M1 (Fig. 4, A and B), showing that LC3s are recruited to the sites where VPs release their vRNPs. To substantiate that LC3s are recruited onto LEs to promote cytoplasmic vRNP release, we immunoprecipitated LC3s and examined the interaction of VP components with LC3s during IAV cytoplasm entry in the presence or absence of Baf. This analysis revealed an interaction between NP and LC3s in IAV-infected cells, which was strongly impaired upon Baf treatment (Fig. 4, C and D). We also examined whether DYNC1I1 interacts with LC3s during IAV cytoplasm entry by immunoprecipitation (IP) and detected binding between these proteins in infected cells (Fig. 4, E and F), consistently with an interactome mapping studies that uncovered an interaction between LC3B and both DYNC1I1 and dynactin 1, another component of the dynein 1 biomolecular motor (*35*, *36*). This binding was specific because kinesin family member 5B (KIF5B), a component of the kinesin biomolecular motors that plays a role in the trafficking of LC3-positive autophagosomes (*37*), was not detected in the coisolates (Fig. 4E). In addition, we did not detect the binding between LC3s and HDAC6, suggesting that LC3 is part of an uncoating mechanism that does not involve HDAC6.

### The dynein adaptor PCNT is required for IAV cytoplasm entry

**Fig. 5.**
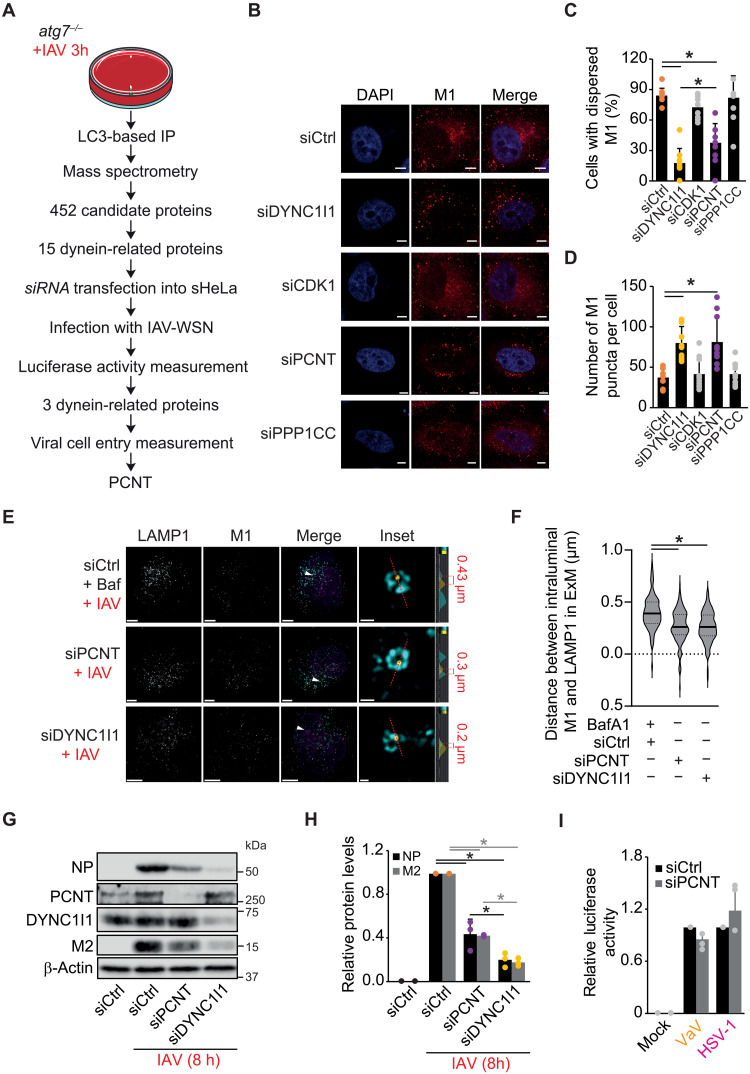
The dynein 1 adaptor PCNT is required for IAV cytoplasm entry. (**A**) Working flow that led to the identification of PCNT as a factor in IAV cytoplasm entry. (**B**) DYNC1I1-, CDK1-, PCNT-, or PPP1CC-depleted sHeLa cells were infected with IAV at an MOI of 10 and processed for IF with the anti-M1 antibody at 3 hpi as in Fig. 1G. Images were acquired using a ZEISS LSM800 microscope. Scale bars, 5 μm. (**C** and **D**) Quantification of both the percentage of cells with dispersed M1 (C) and the amount of M1 puncta per cell (D) in (B). (**E**) sHeLa cells were transfected with siCtrl, siPCNT, or siDYNC1I1 for 48 hours, infected, and processed for ExM as in Fig. 3F. Baf (200 nM) was used to block IAV fusion at LEs in sHeLa cells. Scale bars, ~4.5 μm (maximum projection images) and ~0.2 μm (inset images). (**F**) Quantification of the distance of luminal M1 puncta from the LAMP1-positive LE membrane in single slices of expanded cells shown in (E). (**G**) DYNC1I1- or PCNT-depleted sHeLa cells were infected with IAV at an MOI of 0.1 for 8 hours. Cell extracts were examined by WB with anti-NP, M2, PCNT, DYNC1 I1, and β-actin antibodies. (**H**) NP and M2 level quantification in (G). Bars represent average amounts relative to infected cells treated with siCtrl. (**I**) PCNT-depleted sHeLa cells were infected with luc-HSV-1 or luc-VaV at an MOI of 1 for 6 hours. Luciferase activity in cell extracts was then measured. Data represent the average luciferase activities expressed relative to the siCtrl for each virus. Error bars represent SDs [*n* = 10 in (C) and (D), 50 cells counted per repeat; *n* = 3 in (F), (H), and (I)]. Asterisks indicate significant differences. h, hours.

Since LC3s often interact with dynein biomolecular motors via adaptor proteins such as FYVE and coiled-coil domain autophagy adaptor 1 (FYCO1), c-Jun N-terminal kinase (JNK)-interacting protein 1 (JIP1), and rab-interacting lysosomal protein (RILP1) (*25*, *38*, *39*), we decided to gain more insight into the molecular interaction between vRNPs, LC3s, and DYNC1I1 by identifying the adaptor protein(s) that functionally link LC3s with dynein 1. We therefore subjected cell lysates from *atg7^−/−^* cells infected with IAV for 3 hours to IP with the anti-LC3 antibody before identifying the coisolated proteins by mass spectrometry (MS) (Fig. 5A and table S1). The *atg7^−/−^* cells were used instead of the WT to exclude dynein biomolecular motor complexes involved in autophagosome trafficking. We found that all the vRNP components bind to LC3s, in agreement with our data that LC3s interact with IAV vRNPs (Fig. 4, C and D, and table S1). We scrutinized this hit list for proteins that are known to directly or indirectly interact with dyneins and found 15 proteins that fulfill this criterion (Fig. 5A and table S2). To determine which of them is involved in the IAV viral cycle, we individually knocked them down in sHeLa cells by siRNA before inoculating the IAV A/WSN/1933 strain (IAV-WSN) strain, which expressed luciferase (*40*). As positive controls, we included siRNAs targeting not only DYNC1I1 but also ATP6V1A and ATP6V1B2 (*41*), two subunits of the vacuolar H^+^-ATPase. As expected, silencing of ATP6V1A or ATP6V1B2 and DYNC1I1 reduced luciferase expression in sHeLa cells (fig. S4A). Bridging integrator 1 (BIN1), cyclin-dependent kinase 1 (CDK1), palladin (PALLD), pericentrin (PCNT), or protein phosphatase 1 catalytic subunit gamma (PPP1CC) knockdown led to a decrease in IAV replication. To corroborate these data with a WT strain, we silenced these proteins before examination of NP or NS1 gene expression at 3 or 4 hpi upon IAV inoculation. We observed a reduction in NP and NS1 expression early in IAV infection only upon CDK1, PCNT, or PPP1CC depletion (fig. S4, B and C). To establish which of those proteins is required for IAV cytoplasm entry, we then used the cytoplasm entry assay to determine which of these proteins is required for VP uncoating and uncovered that the knockdown of PCNT, but not CDK1 or PPP1CC, significantly impaired IAV cytoplasm entry (Fig. 5, B to D). To corroborate whether PCNT is involved in IAV uncoating at LEs, we first examined the localization of M1 by IF and found increased colocalization between M1 and LAMP1 in PCNT-depleted cells (fig. S4, D and E). The extent of colocalization between these two proteins in siPCNT-treated cells, however, was less pronounced than in DYNC1I1-depleted cells (fig. S4E), suggesting a partial block in IAV exit at LEs. We then examined VP distribution inside LEs in cells depleted of PCNT or DYNC1I1 as a control by ExM. This analysis revealed that similarly to what was observed in LC3TKO cells (Fig. 3, F and G), luminal M1 puncta were more adjacent to the LE membrane in both PCNT and DYNC1I1 knockdown cells in comparison to Baf-treated cells (Fig. 5, E and F). We also verified whether not only PCNT but also DYNC1I1 were involved in IAV binding to the cell surface and subsequent endocytosis and found no significant differences between PCNT- or DYNC1I1-depleted cells and the control (fig. S4, F to H), indicating that these two proteins are not important for these two steps of the viral cycle.

To investigate the relevance of PCNT in IAV infection, we infected PCNT-depleted cells and evaluated viral protein expression at 8 and 16 hpi. NP and M2 levels were significantly decreased in PCNT knockdown cells (Fig. 5, G and H, and fig. S5, A and B). Extracellular amounts of infectious IAV VPs were also reduced at 16 hpi in the absence of PCNT or DYNC1I1 (fig. S5C), which was used as a control, showing the critical role of PCNT in IAV infection. We also checked whether PCNT is specifically required for IAV infection by infecting sHeLa cells, depleted or not of PCNT, with luc-HSV-1 and luc-VaV, before assessing luciferase activity at 6 hpi. No differences in the replication of these viruses were detected (Fig. 5I).

### IAV infection does not rely on the PCNT role in centrosome assembly

**Fig. 6.**
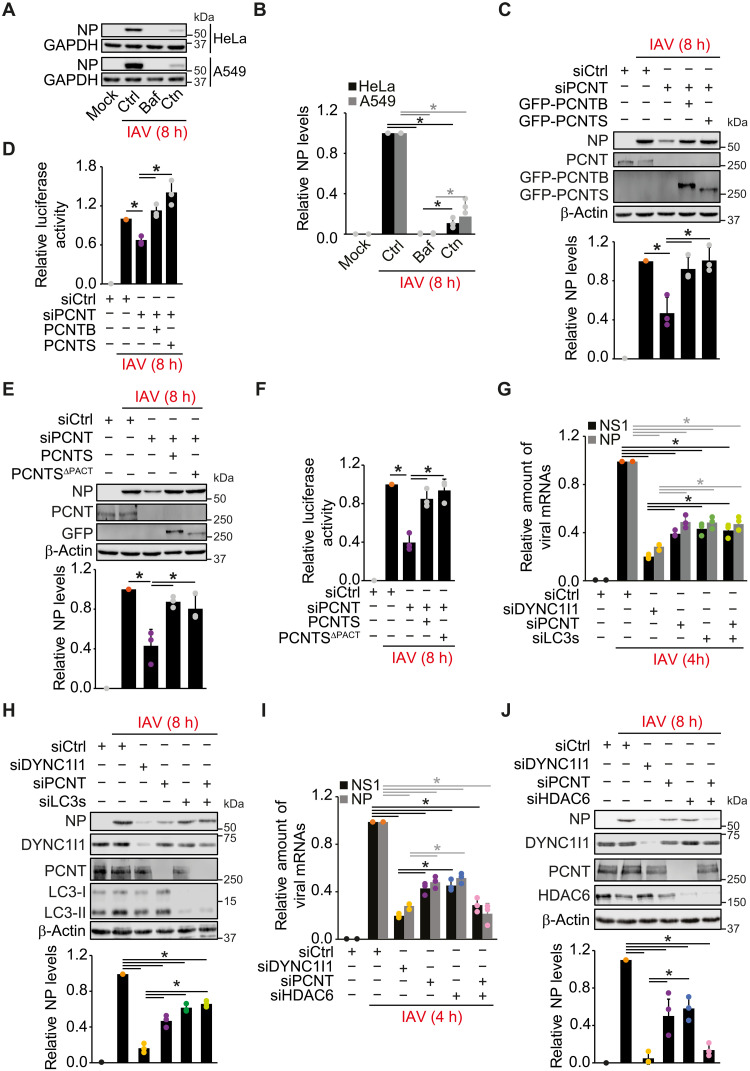
Both forms of PCNT can promote IAV cytoplasm entry. (**A**) sHeLa and A549 cells were infected with IAV at an MOI of 0.1 for 8 hours in the presence of 200 nM Baf or 100 nM Ctn before WB with the indicated antibodies. (**B**) NP levels in (A) relative to siCtrl. (**C**) PCNT-depleted sHeLa cells transfected with the GFP-PCNTB or GFP-PCNTS plasmid were infected with IAV at an MOI of 0.1 for 8 hours and examined by WB with the indicated antibodies. NP levels are relative to siCtrl. The GFP antibody was used for GFP-PCNTS. The PCNT antibody only detects PCNTB. (**D**) Cells as in (C) were infected with WSN-luc IAV for 16 hours, and luciferase activity was measured relative to infected siCtrl cells. (**E**) PCNT-depleted sHeLa cells were transfected with the GFP-PCNTS or GFP-PCNTS^ΔPACT^ plasmid before IAV infection at an MOI of 0.1 for 8 hours and examined by WB analysis with the indicated antibodies. NP levels are relative to siCtrl. (**F**) Cells as in (E) were infected with WSN-luc IAV for 16 hours, and luciferase activity was measured relative to siCtrl. (**G**) DYNC1I1-, PCNT-, or LC3/PCNT-depleted sHeLa cells were infected with IAV at an MOI of 0.1 for 4 hours, and NP and NS1 mRNA levels were quantified by RT-PCR, normalized to GAPDH, and expressed relative to siCtrl. (**H**) Cells from (G) were infected with IAV for 8 hours before WB with the indicated antibodies. NP levels are relative to siCtrl. (**I**) DYNC1I1-, PCNT-, HDAC6-, or PCNT/HDAC6-depleted sHeLa cells were infected with IAV at an MOI of 0.1 for 4 hours, and NP and NS1 mRNA levels were quantified as in (G). (**J**) Cells as in (I) were infected for 8 hours before WB with the indicated antibodies. NP levels are relative to siCtrl. Error bars represent the SDs [*n* = 3 in (B) to (J)]. Asterisks indicate significant differences. h, hours.

PCNT is an adaptor protein that interacts with dynein 1 via the dynein light intermediate chain (DYNC1LI1) to transport specific components such as centrosomal protein 192 (CEP192) and centrosomal protein 215 (CEP215) to centrosomes, along MTs (*42*, *43*). Thus, PCNT contributes to the assembly and function of the centrosomes and the mitotic spindles, which are key for chromosome segregation during cell division. PCNT is also a centrosome component and a core pericentriolar material (PCM) protein (*44*). To investigate whether the role of PCNT in IAV infection is connected to its function at centrosomes, we used centrinone (Ctn), a Polo-like kinase 4 inhibitor that blocks PCM gathering and centrosome assembly (*45*). We assessed IAV replication by quantifying NP levels in both sHeLa and A549 cells upon Ctn treatment. As reported by others (*46*), Ctn treatment induced a dispersion of PCNT without affecting the overall organization of MTs, something that was not caused by IAV infection alone (fig. S6A). Ctn blocked IAV infection to an extent comparable to the one with Baf (Fig. 6, A and B), suggesting that centrosomes play an important role in facilitating IAV infection.

PCNT exists in two splicing isoforms, PCNTB and PCNTS, with molecular weights of ~380 and 250 kDa, respectively (*47*). Both isoforms are characterized by one C-terminal pericentrin-A-kinase anchor protein 450 (AKAP450) centrosomal targeting (PACT) motif and three (PCNTB) or one (PCNTS) coiled-coil domain (*48*). PCNT proteins also interact with both protein kinase A and dynein 1, although the specific binding domains remain to be identified (*42*, *47*, *49*, *50*). To investigate the specific relevance of each PCNT isoform in IAV viral life cycle, we examined IAV infection in PCNT-depleted cells complemented with either green fluorescent protein (GFP)–PCNTS or GFP-PCNTB and found that both isoforms restored WT and WSN-luc IAV infection to a level comparable to the siCtrl-treated cells (Fig. 6, C and D).

Since disruption of centrosomes using Ctn leads to the disruption of multiple cell functions, potentially indirectly impairing the IAV viral cycle, we turned to the PACT domain, which is instrumental in the centrosomal localization of PCNT proteins (*51*). We reasoned that ablation of this domain would disrupt the localization and function of PCNT proteins at centrosomes but preserve the remaining cellular roles of these proteins. We assessed NP expression levels in PCNT knockdown cells back-transfected with plasmids expressing GFP-PCNTS or GFP-PCNTS^ΔPACT^, a variant of PCNTS lacking the PACT domain. Both fusion proteins were able to restore both WT and WSN-luc IAV infection in PCNT-silenced cells (Fig. 6, E and F).

To exclude that the knockdown of PCNT, LC3s, or DYNC1I1 affects MTs and/or centriole organization, potentially leading to indirect negative impacts on IAV infection, we examined MTs and centrioles by IF in PCNT-, LC3-, or DYNC1I1-depleted cells upon IAV infection using antibodies against α-tubulin and centrin (fig. S6, B and C). Notably, depletion of PCNT, LC3s, or DYNC1I1 did not alter the assembly of both MTs and centrioles in IAV-infected cells.

### LC3s and PCNT form a dynein adaptor complex involved in IAV cytoplasm entry

To confirm that PCNT and LC3s are part of the same IAV cytoplasm entry pathway, we tested viral gene and protein expression in cells depleted of PCNT and/or LC3s. No significant difference in the expression of viral genes NS1 and NP or the viral NP protein was observed between the double knockdown and the single depletion of PCNT or LC3s, suggesting that PCNT function in IAV infection is associated with LC3s (Fig. 6, G and H). We also explored whether the function of PCNT in IAV infection is redundant with that of HDAC6 as the one of LC3s. While the individual knockdown of PCNT or HDAC6 reduced IAV infection, simultaneous silencing of PCNT and HDAC6 strongly inhibited viral gene and protein expression to a similar extent as DYNC1I1 depletion (Fig. 6, I and J). NP levels were assessed in LC3TKO cells individually depleted of PCNT, HDAC6, or DYNC1I1 as well. While the knockdown of HDAC6 or DYNC1I1 exacerbated the IAV infection defect of LC3TKO cells, silencing of PCNT did not (fig. S6D). This result reinforced the notion that HDAC6 and PCNT/LC3s are part of two different and redundant dynein 1–dependent pathways for IAV uncoating. Since the IAV A/Puerto Rico/8/1934 strain (PR8) and WSN strains used here both belong to the hemagglutinin type 1–neuraminidase type 1 (H1N1) subtype that is part of the IAV group 1, we also examined the relevance of LC3, PCNT, HDAC6, and dynein in the cytoplasm entry of strains from the H5N1 and H3N2 subtypes, which belong to the IAV group 1 and group 2, respectively (*52*). As shown in fig. S7, the LC3-PCNT– and HDAC6-dependent cytoplasm entry mechanisms were also required for the H5N1 strain to infect cells. In contrast, the cytoplasm entry of the H3N2 strain partially involved the LC3-PCNT module and dynein but not HDAC6. This result indicates that not all the IAV subtypes use the same strategies for cytoplasm entry and those in the group 2, in addition to relying on the LC3-PCNT-dynein pathway, may rely on one or more mechanisms that do not depend on HDAC6 and dynein.

**Fig. 7.**
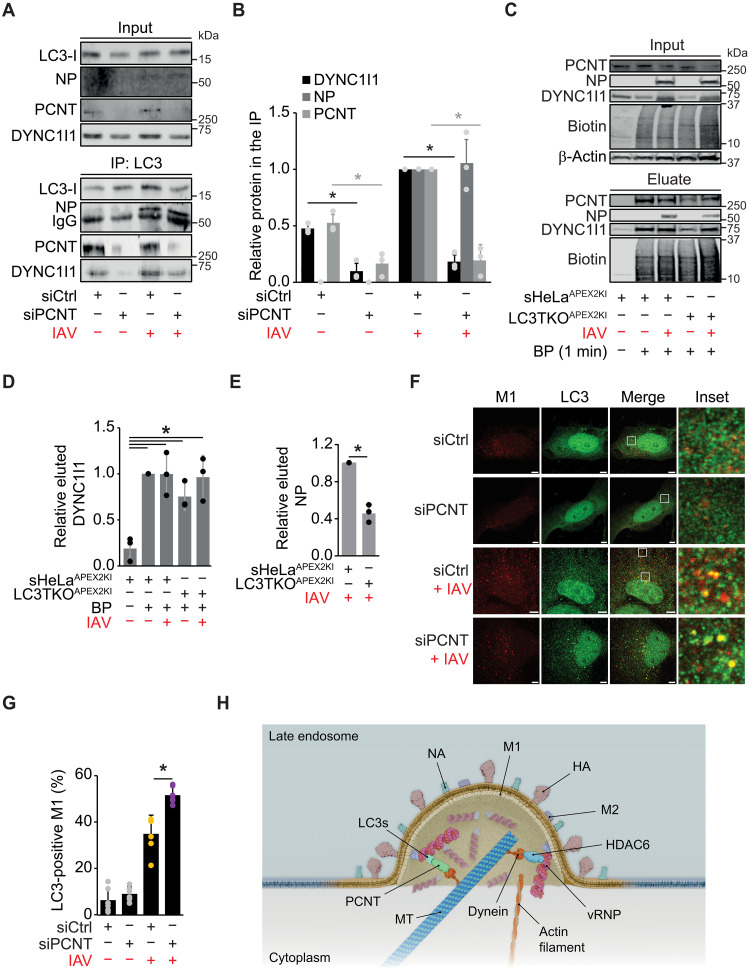
LC3-PCNT constitutes a dynein 1 adaptor complex involved in IAV cytoplasm entry. (**A**) PCNT-depleted *atg7^−/−^* cells were infected with IAV at MOI 30 for 3 hours, and cell extracts were subjected to IP with an anti-LC3 antibody before examining the input and the coisolated proteins by WB with anti-LC3, NP, PCNT, DYNC1I1, and ΙgG (control) antibodies. (**B**) DYNC1I1, NP, and PCNT bound to LC3s in (A) relative to the infected siCtrl cells. (**C**) sHeLa^APEX2KI^ and LC3 ^APEX2KI^ cells were infected with IAV as in Fig. 1G, but 500 μM biotin phenol (BP) and 1 mM H_2_O_2_ were added 30 and 1 min, respectively, before isolating biotinylated proteins. sHeLa^APEX2KI^ cells without BP incubation were used as a negative control. The input and the affinity-purified proteins were analyzed by WB with antibodies against biotin, NP, PCNT, DYNC1I1, or β-actin. (**D** and **E**) Biotinylated DYNC1I1 (D) and NP (E) in (C) relative to the noninfected sHeLa^APEX2KI^ cells. (**F**) PCNT-depleted *atg7^−/−^* cells were processed for IF as in Fig. 1G with antibodies against M1 and LC3. Insets highlight colocalization between M1 and LC3. Images were acquired using a ZEISS LSM800 microscope. Scale bars, 5 μm. (**G**) Percentage of the LC3-positive M1 puncta in (F). (**H**) Model for IAV host cytoplasm entry. The lower pH of LEs triggers the fusion between endocytoses IAV VPs at LEs. Uncoating and cytoplasmic vRNP release is mediated by two dynein-dependent systems that take advantage of the pulling force of MT-based motors. vRNPs are linked to dynein motors via the LC3-PCNT adaptor complex or HDAC6, which binds ubiquitin. It is unknown which vRNP components interact with LC3s and HDAC6. Error bars represent SDs [*n* = 3 in (B), (D), and (E); *n* = 5 in (C), 50 cells counted per repeat]. Asterisks indicate significant differences.

To determine whether PCNT is the dynein 1 adaptor that promotes IAV uncoating together with LC3s, we performed an IP using the LC3 antibody in *atg7^−/−^* cells depleted of PCNT. NP interacted specifically with LC3s under both siCtrl and siPCNT conditions, while the PCNT deficiency only impaired the binding between LC3s and DYNC1I1 (Fig. 7, A and B). These data indicated that the PCNT bridges the vRNP-LC3 complex with dynein 1. To corroborate this result, we also tried to examine whether PCNT binding to NP depends on LC3s, but our anti-PCNT antibody did not work in IP. Thus, we generated sHeLa^APEX2KI^ and LC3TKO^APEX2KI^ cells by C-terminally tagging endogenous PCNT with ascorbate peroxidase 2 (APEX2) using CRISPR-Cas9 editing and carry out proximity biotinylation to isolate neighbor proteins with streptavidin-conjugated beads (*53*). sHeLa^APEX2KI^ and LC3TKO^APEX2KI^ cells express functional PCNT-APEX2 chimeras, as IAV infection was identical to the control cells and show a biotinylation pattern (fig. S8, A and B). sHeLa^APEX2KI^ and LC3TKO^APEX2KI^ cells were inoculated with IAV before performing the biotinylation reaction at 3 hpi and isolating the biotinylated neighbors. In addition to biotinylating itself, PCNT-APEX2 also modified DYNC1I1 under all conditions (Fig. 7, C and D). In contrast, NP proximity to PCNT was strongly reduced in the absence of LC3s (Fig. 7, C and E), indicating that PCNT interacts indirectly with vRNP, via LC3s, and directly with the dynein 1. Overall, this approach confirmed the vRNP–LC3–PCNT–dynein 1 interaction axis.

To further sustain this notion, we explored whether PCNT is important for the association of LC3s with LEs during IAV cytoplasm entry by examining M1 and LC3 distribution in *atg7^−/−^* cells depleted of PCNT. Inoculation of *atg7^−/−^* cells treated with the siCtrl or siPCNT induced the formation of LC3 puncta colocalizing with M1 under both conditions (Fig. 7F), indicating that LC3 recruitment to LEs during IAV cytoplasm entry does not depend on PCNT. Moreover, we observed higher colocalization between LC3 and M1 in *atg7^−/−^* cells depleted of PCNT (Fig. 7, F and G), suggesting that PCNT may drive vRNP-LC3 complex away from LEs during IAV uncoating.

## DISCUSSION

It has previously been shown that HDAC6 binds unanchored ubiquitin chains present in IAV virions to engage the APM and facilitate VP uncoating by exploiting the physical forces generated by MT- and actin-associated biomolecular motors (*4*, *6*, *54*). However, HDAC6 depletion only partially inhibits IAV cytoplasm entry, whereas dynein deficiency completely blocks it. Here, we show that a dynein adaptor complex composed by LC3s and PCNT promotes IAV uncoating at LEs as well. Our findings provide a mechanistic explanation for IAV cytoplasmic entry in the absence of a functional APM and support the notion that IAV uncoating at LEs relies on two redundant mechanisms (Fig. 7H). PCNT, LC3, and HDAC6 are involved in multiple intracellular pathways and are ubiquitously expressed in many cell types, including in HeLa, U2OS, and A549 cell lines used in this study and in macrophages (fig. S9). All these cell types are generally infected by IAV, and although it cannot be completely excluded, our data suggest that IAV has not developed two cytoplasmic entry mechanisms to target different cell types but, instead, allows IAV to adopt a dual strategy to increase the chances of infection and consequently of propagation. Our data also suggest that certain IAV strains, possibly those belonging to the group 2, may have additional stratagems to enter the host cell cytoplasm, although the LC3-PCNT-dynein module seems to be a universally important. Future investigations are needed to obtain a more comprehensive picture about how different IAV groups and subtypes infect cells.

PCNT binds DYNC1LI1 to regulate centrosome positioning, MT organization, and intracellular transport of various cellular cargoes (*42*, *50*, *55*). It has previously been shown that IAV infection can lead to spindle body misorientation and a concomitant decrease in PCNT at the spindle poles when infection was assessed in A549 cells at 15 hpi (*56*). We did not observe any apparent PCNT mislocalization nor redistribution of MTs or centrosomal components in IAV-infected HeLa and A549 cells at 8 hpi. These observations suggest that IAV is unlikely to subvert PCNT to reorganize spindle bodies to promote its viral cycle. This scenario is also ruled out by our experiment showing that PCNTS^ΔPACT^ sustains IAV infection, indicating that PCNT recruitment and function at centrosomes are not required for the IAV viral cycle. Since dynein 1 is absolutely required for IAV cytoplasm entry, our data infer that IAV subverts PCNT to exploit the dynein 1 biomolecular motors for VP uncoating. However, we cannot exclude the possibility that vRNP anchoring to dynein 1 not only via PCNT but also via HDAC6 is important for vRNP transport to the nucleus to promote their translocation into the nucleus and, thus, IAV replication.

Many studies have investigated the role of the autophagy machinery during IAV infection, but whether autophagy is beneficial for the IAV viral life cycle remains unclear (*57*, *58*). Initial reports showed that IAV infection induces the formation of autophagosomes but blocks their fusion with lysosomes (*59*, *60*). However, autophagy inhibition does not interfere with IAV replication, but it rather compromises IAV-infected cell survival (*61*, *62*). This latter finding is consistent with other recent studies showing that autophagy is dispensable for IAV infection (*63*–*66*), which is in line with our observation that an intact ATG machinery is not required for the IAV viral cycle. LC3s have so far been linked to IAV infection mainly through their role in the selective type of autophagy known as xenophagy and LC3-associated phagocytosis (LAP). In IAV-infected cells, GFP-LC3B initially distributes in perinuclear puncta (*60*, *65*, *67*), which probably represent autophagosomes and/or endosomes decorated by LC3s. The redistribution of GFP-LC3B to endosomes is induced by the viral M2 proton channel, which raises the pH of endosomes triggering both autophagy and LAP (*61*, *68*). GFP-LC3B also localizes to the plasma membrane at the late time points of IAV infection through a direct interaction with M2 at this location (*65*). In our study, however, we describe a function of LC3s in the IAV viral cycle. We show that nonlipidated LC3s are required for IAV uncoating at LEs. This unconventional function of LC3s has very likely been overlooked, as HA-tagged LC3B does not complement the IAV infection defect of LC3TKO cells (fig. S10, A and B), indicating that N-terminal tagging of LC3s hampers this function. Nonlipidated LC3s have previously been implicated in the ERAD tuning pathway as well as MHV, EAV, JEV, CVB3, and *Chlamydia* infections (*16*–*21*), but their specific molecular role in these contexts remains unknown. LC3-II has been detected in Epstein-Barr virus virions (*69*). Consistent with a previous study (*70*), however, we have been unable to detect LC3s in the IAV particles in preliminary analyses (fig. S10, C and D). These results suggest that LC3s are probably not already associated with vRNPs within IAV virions.

In conclusion, our study has uncovered a pathway crucial for IAV uncoating at LEs (Fig. 7H). This knowledge provides a more complete picture of the mechanisms that IAV uses to enter the cytoplasm of host cells and may be important to develop effective anti-IAV treatments targeting this step of the IAV viral cycle.

## MATERIALS AND METHODS

### Cell culture and virus

U2OS, A549, sHeLa, and Madin-Darby canine kidney (MDCK), MDCK-HA cell lines were maintained in Dulbecco’s modified Eagle’s medium (DMEM; Cambrex Bioscience, Walkersville, MD), supplemented with 10% fetal bovine serum (FBS; Bodinco Alkmaar, The Netherlands), 100 IU/ml penicillin, and streptomycin (100 μg/ml) (both from Life Technologies, Rochester, NY). The *atg7^−/−^*, *atg13^−/−^*, and *atg16L1^−/−^* knockout U2OS cell lines were generated using the CRISPR-Cas9 system and have been described elsewhere (*71*–*73*). The LC3TKO and GABATKO cell lines and parental sHeLa cells were provided by Lazarou and colleagues (*27*).

The WT A/PR8/H1N1, A/Perth/H3N2, and A/NIBRG14/H5N1 IAV strains were produced in embryonated chicken as previously described (*74*). The WSN-luc IAV strain was propagated in MDCK-HA cells, a gift of García-Sastre and colleagues (*40*), and the virus titer was determined by measuring the median tissue culture infectious dose (TCID_50_) per milliliter on U2OS cells. The luc-HSV-1 and luc-VaV viruses were propagated in baby hamster kidney (BHK) and African green monkey kidney BSC-40 cells, respectively (*41*). Virus titers of the culture supernatants were determined with a diluted factor of five on LR7 cells, and the TCID_50_ per milliliter was calculated according to the Reed-Muench method (*75*).

### Human monocyte-derived macrophages

Human peripheral blood mononuclear cells (PBMCs) were purified from buffy coats obtained from either the blood bank at the Department of Clinical Immunology, Aarhus University Hospital, Aarhus, Denmark or Nordic Biosite. Macrophages were derived from anonymous blood donors, and according to Danish law, reporting to an ethics committee is not required. Buffy coats were diluted 1:2 in warm phosphate-buffered saline (PBS; Cytiva, Marlborough, MA), and PBMCs were isolated using PluriMate II 50-ml centrifugation tubes (Pluriselect, Leipzig, Germany) filled with 17 ml of Histopaque-1077 (Sigma-Aldrich, Saint Louis, MO) by centrifugation at 800*g* for 30 min at room temperature (RT; acceleration 4 and deacceleration 3). Cells were washed once in PBS, and red blood cells (RBCs) were lysed in RBC lysis buffer (150 mM NH_4_Cl, 10 mM KHCO_3_, and 0.5 mM EDTA). PBMCs were frozen down in Cryostor CS10 (Stemcell Technologies, Vancouver, Canada) and stored in liquid nitrogen. On the day of monocyte purification, frozen PBMCs were defrosted in a water bath. Cells were then washed in RPMI 1640 and, afterward, incubated for 15 min at RT with deoxyribonuclease (DNase) solution 1 (Stemcell Technologies) diluted 1:10 with RPMI 1640. The monocytes were then isolated by negative selection using the EasySep Monocyte Enrichment Kit (Stemcell Technologies) according to the manufacturer’s protocol. Monocytes were subsequently cultured in RPMI 1640 (Thermo Fisher Scientific, Waltham, MA) with 10% heat-inactivated FBS (Biowest, Nuaille, France), penicillin (100 U/ml), streptomycin (100 μg/ml), and 2 mM glutamine (Thermo Fisher Scientific). A total of ~2 × 10^5^ monocytes were plated in one 12-well plate and either incubated in the culture medium supplemented with macrophage colony-stimulating factor (10 ng/ml; Thermo Fisher Scientific) for the differentiation into the Mac1 phenotype or the medium supplemented with granulocyte-macrophage colony-stimulating factor (10 ng/ml; Thermo Fisher Scientific), transforming growth factor–β1 (5 ng/ml; Thermo Fisher Scientific), and interleukin-10 (5 ng/ml; Thermo Fisher Scientific) for differentiation into the Mac2 phenotype for 7 days.

### Plasmids and siRNAs

The plasmids expressing LC3A, LC3B, LC3C, 3× HA-LC3B, LC3B^G120A^, LC3B^G120A*^, LC3B^G120*^, LC3B^T12A^, LC3B^T12D^, LC3B^T50A^, LC3B^T50E^, and LC3B^K49Q_K51Q^ were generated by amplifying these coding sequences by PCR using appropriate primers from pDest27-LC3A, pDest27-LC3B, and pDest27-LC3C constructs, provided by Faure and colleagues (*76*) and subsequent cloning into the pCDNA3.1 vector using Bam HI/Xho I or Hind III/Xho I, creating the pCDNA3.1-LC3A, pCDNA3.1-LC3B, pCDNA3.1-LC3C, pCDNA3.1 to 3× HA-LC3B, pCDNA3.1-LC3B^G120A^, pCDNA3.1-LC3B^G120A*^, and pCDNA3.1-LC3B^G120*^. All constructs were verified by DNA sequencing. The pEGFP-LC3B plasmid was provided by Kampinga and colleagues (*77*). p3xFLAG-CMV10-eGFP-hPCNTB and p3xFLAG-CMV10-eGFP-hPCNTS constructs were gifts from Engel and colleagues (*47*), and the hPCNTS^ΔPACT^ was generated by amplifying the gene by PCR using appropriate primers from p3xFLAG-CMV10-eGFP-hPCNTS and then cloning it back into p3xFLAG-CMV10-eGFP using Avr II/Age I, creating the construct p3xFLAG-CMV10-eGFP-hPCNTS^ΔPACT^. The SMARTpool siRNA and the single siRNA used in this study were listed in table S3, and the cherry-picked siRNA library targeting the MS hits (table S2) were purchased from GE Healthcare (Chicago, IL).

### Chemical compounds

The chemical compounds used in this study were Baf (Tebubio, Paris, France), Noc (Sigma-Aldrich), CilioD (Merck Millipore, Burlington, MA), LatA (Sigma-Aldrich), cycloheximide (Sigma-Aldrich), and Ctn (MedChemExpress, Monmouth Junction, NJ).

### siRNA and plasmids transfection

Cells grown in 96-, 24-, 12-, or 6-well plates were transfected with 20 nM control siRNA or siRNA targeting the indicated genes for 48 hours using 0.1, 0.5, 1, and 2 μl of Lipofectamine RNAiMAX (Invitrogen, Waltham, MA), respectively, according to the manufacturer’s protocol. Cells grown in 12-well plates were transfected with 1 μg of plasmid for 48 hours using 3 μl of the FuGENE HD Transfection Reagent (Promega, Madison, WI), also following the manufacturer’s protocol.

### Generation of APEX2 knock-in cells using CRISPR-Cas9 editing

Guide sequences for the integration at the 3′ of the *PCNT* gene (CACCGTCCTCAAATAGAATTGTCGC and AAACGCGACAATTCTATTTGAGGAC) were hybridized before cloning into the pX458 vector (Addgene, catalog no. 48138) between the two Bbs I sites, while the donor DNA comprising the sequences coding for APEX2 and the puromycin resistance cassette flanked by homology arms of 800 base pairs on either side of the insertion in *PCNT* was synthesized in pEX-A258 vector from Eurofins Scientific (Luxembourg). The donor DNA also contained a puromycin resistance gene with a stop codon at its 5′ to select for successful edits.

sHeLa and LC3TKO cells in six-well plates were cotransfected with the plasmids carrying the guide sequences and the donor DNA using the FuGENE HD Transfection Reagent for 48 hours before selecting the positive cells by addition of puromycin (2 μg/ml) to the DMEM supplemented with 10% FBS. Cells were trypsinized after 72 hours and seeded in 10-cm dishes containing DMEM culture medium and puromycin (2 μg/ml) to grow single-cell clones. Those were then picked and grown in abovementioned medium before amplifying the *PCNT* locus by PCR and DNA sequencing to determine which clones have a correct integration of *APEX2*. Last, APEX2-PCNT expression and biotinylation were assessed by WB. This procedure generated the sHeLa^APEX2KI^ and LC3TKO^APEX2KI^ cell lines.

### IF analyses

Cells were grown on 12-mm coverslips, transfected, and/or infected for 24 hours before being fixed with 4% paraformaldehyde at the indicated time points and permeabilized in PBS [0.137 M NaCl, 0.0027 M KCl, 0.01 M Na_2_HPO_4_, and 0.0018 M KH_2_PO_4_ (pH 7.4)] containing 0.1% Triton X-100 (Sigma-Aldrich) and 1% of bovine serum albumin (BSA; Sigma-Aldrich). After blocking with PBS containing 1% FBS, preparations were first incubated with primary antibodies at RT for 1 hour and after washing with PBS, with a secondary antibody conjugated to either Alexa Fluor 488 or Alexa Fluor 568 (Molecular Probes, Eugene, OR) also at RT for 1 hour. The primary antibodies were against LC3 (MBL international, Woburn, MA, #PM036), GABARAP (a gift of Takeda Pharmaceuticals, Osaka, Japan), NP (Bio-Rad, Berkeley, CA or Abcam, Cambridge, UK, #MCA400), M1 (American Type Culture Collection, Manassas, VA, #M2-1C6-4R3), M2 (Abcam, #ab5416), SNX1 (Proteintech, Rosemont, IL, #10304-1-AP), LAMP1 (Cell Signaling Technology, Danvers, MA, #9091S), PCNT (Sigma-Aldrich, #HPA016820), centrin (Merck Millipore, #04-1624), and α-tubulin (tubulin α4a; Sigma-Aldrich, #T5168). Preparations were lastly incubated with ProLong Gold Antifade Mountant with 4′,6-diamidino-2-phenylindole (DAPI; Thermo Fisher Scientific) before imaging. Fluorescence signals were captured with a Leica sp8 confocal microscope (Leica Microsystems, Wetzlar, Germany), a DeltaVision RT fluorescence microscope equipped with a CoolSNAP HQ camera (GE Healthcare), a TissueFAXS microscope (ZEISS, Jena, Germany), or a ZEISS LSM800 confocal microscope (ZEISS). Quantifications of puncta number and size and colocalization events of the acquired images were carried out using the Icy software (http://icy.bioimageanalysis.org) using spot detector plugin. Quantifications of M1 cytoplasmic and puncta distribution were assessed using the ImageJ software using the intensity plot file (*78*). Each experiment was examined at least in 50 cells.

### WB analyses

Cells grown in 12-well plates were washed with ice-cold PBS and, after addition of 100 μl of 2× Laemmli sample buffer [65.8 mM tris-HCl (pH 6.8), 26.3% glycerol, 2.1% SDS, and 0.01% bromophenol blue], incubated on ice for 30 min, sonicated for 1 min, and boiled. Equal protein amounts were separated by SDS–polyacrylamide gel electrophoresis (PAGE) and subsequently transferred on polyvinylidene difluoride membranes (Sigma-Aldrich). Proteins of interest were detected using specific antibodies against LC3, GABARAP, NP, M2, ATG7 (Cell Signaling Technology, Danvers, MA, #2631S), ATG13 (Rockland Immunochemicals, Pottstown, PA, #SAB4200100), β-actin (Merck Millipore, #MAB1501), PCNT (Sigma-Aldrich, #HPA016820), GFP (monoclonal; Takara, Shiga, Japan, #632381), GFP (polyclonal; Abcam, #ab6556), DYNC1I1 (Novus, St. Charles, MO, #NBP1-87972), HDAC6 (Abcam, #ab1440), biotin (Rockland, #100-4198), vinculin (Cell Signaling Technology, #13901S), glyceraldehyde-3-phosphate dehydrogenase (GAPDH; Thermo Fisher Scientific, #4333764T), and secondary antibodies conjugated to Alexa Fluor 680 or Alexa Fluor 800 (Molecular probes). The signals were captured with an Odyssey Imaging System (LI-COR Biosciences, Lincoln, NE), and the protein signal intensities were quantified using the ImageJ software (*79*) and normalized to the loading control, i.e., β-actin or GAPDH.

### IP experiments

sHeLa cells were seeded in a 15-cm petri dish at a density of 1 × 10^6^ cells and incubated overnight until a confluency of ~80%. Cells were then inoculated with IAV at a multiplicity of infection (MOI) of 30 at 37°C for 3 hours, washed three times with ice-cold PBS, and lysed with 500 μl of IP buffer [20 mM tris-HCl (pH 7.5), 150 mM NaCl, 5 mM MgCl_2_, 0.5% NP-40, 10% glycerol, 4 mM NaF, 2 mM β-glycerophosphate, 200 μM sodium vanadate, and 1 mM dithiothreitol] supplemented with the cOmplete EDTA-free protease inhibitor cocktail (Roche, Basel, Switzerland). Cell lysis was performed on ice by passing the cells through a 27-gauge needle (BD, Franklin Lakes, NJ). After incubation on ice for 30 min, lysates were centrifuged at 15,000*g* for 30 min to remove the insoluble material. Five microliters of the sample were taken and boiled with 2× Laemmli sample buffer; this served as the input control. The rest of the supernatant was added to 15 μl of the protein A–sepharose beads (Abcam), prewashed with the IP buffer, and incubated at 4°C for 6 hours on a rotating wheel to clear the cell lysate of components that nonspecifically binds to the protein A–sepharose beads. Upon centrifugation at 1000*g* for 1 min at 4°C, the supernatant was mixed with 5 μl of anti-LC3 antibody (MBL International) and incubated at 4°C overnight on a rotating wheel. Fifteen microliters of the protein A–sepharose beads prewashed with the IP buffer were then added to the lysate and incubated on a rotating wheel for an additional 6 hours at 4°C. The protein A–sepharose beads were subsequently washed five times with 1 ml of ice-cold IP buffer before boiling them in 40 μl of 2× Laemmli sample buffer. Inputs and IP samples were lastly separated by SDS-PAGE, and, upon WB, membranes were probed with antibodies against LC3, NP, M1, HDAC6 (Cell Signaling Technology, #7558S), PCNT, and DYNC1I1 or rabbit serum.

### Luciferase measurements

Cells grown in 96-well plates and infected with luciferase-carrying viruses were washed with PBS and incubated with 50 μl of lysis buffer (Thermo Fisher Scientific) at RT for 15 min before storing the cell lysates at −20°C. Then, 25-μl aliquots of thawed cell lysates were used to measure either Firefly or *Renilla* luciferase expression (depending on the gene carried by the assayed virus) using either the Firefly or *Renilla* luciferase flash assay kit (both from Thermo Fisher Scientific). Alternatively, *Renilla* luciferase activity was measured in the following reaction buffer: 45 mM EDTA, 30 mM sodium pyrophosphate, 1.425 M NaCl, and 10 μM coelenterazine h (Promega). Enzymatic activities were measured using a GloMax-Multi Detection System (Promega) and the following program: 25-μl substrate, 2-s delay, and 10-s measuring.

### RNA isolation, cDNA synthesis, and quantitative PCR

Cells grown in 96-well plates and infected with IAV as indicated were washed with ice-cold PBS and lysed with 30 μl of lysis buffer containing DNase I at RT for 5 min before adding 3 μl of Stop solution at RT for 3 min, according to the manufacturer’s protocol (Power SYBR Green Cells-to-CT kit, Thermo Fisher Scientific). Reverse transcription of the RNA, cDNA synthesis, and quantitative PCR were performed in the CFX Connect Thermal Cycler (Bio-Rad). Primers used were 5′ GTGAGAGAGAGCCGGAACCC and 3′ GCAGGACTTGTGAGCAACCG for the NP gene, 5′ CCACACGTGCTGGAAAGCAG and 3′ GACGCAGGTACAGAGGCCAT for NS1, and 5′ AGCCACATCGCTCAGACAC and 3′ GCCCAATACGACCAAATCC for GAPDH. The levels of NP and NS1 were normalized to those of housekeeping GAPDH according to the comparative cycle threshold method used for quantification, as recommended by the manufacturer’s protocol.

### IAV cell surface attachment and cytoplasm entry assays

The measurement of IAV attachment to cell surfaces was performed in DMEM containing 50 mM Hepes (pH 6.8) and 0.2% BSA as follows. Cells were put on ice for 1 hour before inoculation with IAV at an MOI of 10 in the presence or absence of 1 U of neuraminidase (Sigma-Aldrich). After 2 hours in which they were kept at 4°C, cells were washed three times with ice-cold PBS and processed for RT-PCR as described above.

To assess IAV cytoplasm entry, cells were also put on ice for 1 hour before inoculation with IAV at an MOI of 10 and kept for an additional 2 hours on ice. The medium and unattached VPs were then removed, and cells were washed three times with ice-cold PBS before incubating them with DMEM containing 50 mM Hepes (pH 6.8), 0.2% BSA, and 1 mM cycloheximide at 37°C for 3 hours. Cells were then processed for IF using anti-M1 and anti-LC3 antibody. IAV cytoplasm entry leads to the staining of both the internalized VPs within the endolysosomal system and the intracellularly released M1, which appear as fluorescent puncta and a diffuse staining, respectively (*4*). The presence of cycloheximide blocks the biosynthesis of new viral proteins, and, as a result, the M1 signal is exclusively due to the pool delivered by endocytosed IAV. To exclusively assess endocytosis, the same experiment was repeated, but Baf was added to the culture medium to trap the IAV virions in endosomes.

### Protein MS

Protein A–sepharose IP followed by mass spectrometric analysis was performed as previously described (*80*). sHeLa cells were seeded in two 15-cm petri dishes at a density of 1 × 10^6^ cells and incubated overnight until a confluency of ~80%. Cells were then inoculated with IAV at an MOI of 30 at 37°C for 3 hours, washed three times with ice-cold PBS, and immediately lysed with 500 μl of IP buffer supplemented with the cOmplete EDTA-free proteinase cocktail. Complete cell lysis was obtained by passing the cells through a 27-gauge needle on ice. After incubation on ice for 30 min, lysates were centrifuged at 15,000*g* for 30 min to remove cell debris and insoluble material. The supernatant was then added to 15 μl of the protein A–sepharose beads (Abcam) prewashed with the IP buffer and incubated on a rotating wheel at 4°C for 6 hours to eliminate those components that nonspecifically bind to the beads. Upon centrifugation at 1000*g* for 1 min at 4°C, the supernatants were first incubated with 8 μl of the anti-LC3 antibody (MBL International) at 4°C overnight, before adding 15 μl of the protein A–sepharose beads prewashed with the IP buffer and incubating for additional 6 hours on a rotating wheel at 4°C. The beads were subsequently washed five times with 1 ml of ice-cold IP buffer before boiling them in 40 μl of 2× Laemmli sample buffer. Proteins samples were loaded on an 8% precast RunBlue gel (Abcam) and run at 100 V for 5 min. The gel was stained using InstantBlue (Abcam) and then washed with ultrapure water. The Coomassie-stained bands were excised in one single gel slice, subsequently cut into small pieces, and destained using a mixture of 50 mM NH_4_HCO_3_ and acetonitrile (7:3). Tryptic digestion was performed by addition of 25 μl of 50 mM NH_4_HCO_3_ containing sequencing-grade modified trypsin (10 ng/ml; Promega) and overnight incubation at 37°C. Peptides were extracted using 5% formic acid, followed by a second elution with 5% formic acid in 75% acetonitrile. Samples were dried in a SpeedVac (Thermo Fisher Scientific), centrifuged, and dissolved in 20 μl of 5% formic acid.

The samples were lastly analyzed by nanoLC-MS/MS, consisting of an Ultimate 3000 LC system (Thermo Fisher Scientific) interfaced with a Q Exactive plus mass spectrometer (Thermo Fisher Scientific). Peptide mixtures were loaded onto a 5 mm by 300 μm inner diameter C18 PepMAP100 trapping column with 2% acetonitrile in 0.1% formic acid at an elution speed of 20 μl/min. After loading and washing for 3 min, peptides were eluted onto a 15 cm–by–75 μm i.d. C18 PepMAP100 nanocolumn (Thermo Fisher Scientific). A mobile phase gradient at a flow rate of 300 nl/min and a total run time of 120 min was used, with the following specifications: a 2 to 40% linear gradient of solvent B over 90 min; a 40 to 80% linear gradient of solvent B over 5 min, followed by a return to 2% solvent B. Solvent B was 0:100 water/acetonitrile (v/v) with 0.1% formic acid. In the nanospray source, a stainless-steel emitter (Thermo Fisher Scientific) was used at a spray voltage of 2 kV with no sheath or auxiliary gas flow. The ion transfer tube temperature was 250°C. Spectra were acquired in the data-dependent mode with a survey scan at mass/charge ratio of 300 to 1650 at a resolution of 70,000, followed by MS/MS fragmentation of the top 10 precursor ions at a resolution of 17,500. Singly charged ions were excluded from the MS/MS experiments, and fragmented precursor ions were dynamically excluded for 20 s. The PEAKS Studio version Xpro (Bioinformatics Solutions Inc., Waterloo, Canada) software was used to identify the interactors of LC3s that were detected at least in two of the experiments, which were done in triplicate.

### APEX2-mediated proximity labeling followed by WB

Cells were infected with IAV for 3 hours at an MOI of 10 and 500 μM biotin phenol (BP; Iris Biotech, Marktredwitz, Germany) were added during the last 30 min of this incubation. One millimolar of hydrogen peroxide (H_2_O_2_; Sigma-Aldrich) was added and gently mixed with the medium, and after 1 min incubation at RT, 1 ml of quenching buffer [PBS, 10 mM sodium ascorbate (Sigma-Aldrich), 10 mM NaN_3_, and 5 mM Trolox (Sigma-Aldrich)] was added to stop the proximity labeling reaction. To remove the excess BP and phenoxyl radicals, cells were washed five times with quenching buffer by gentle mixing and another three times with PBS, before scraping cells in quenching buffer. Cells were then collected by centrifugation at 250*g* for 7 min before lysis in 250 μl of ice-cold radioimmunoprecipitation assay (RIPA) buffer (Invitrogen) supplemented with 10 mM sodium ascorbate, 10 mM NaN_3_, 5 mM Trolox, 1 mM phenylmethylsulfonyl fluoride (Sigma-Aldrich), and cOmplete EDTA-free protease inhibitor cocktail. Samples were vortexed at RT for 1 min a total of three times, while being kept on ice in between for 6-min intervals. The lysates were diluted with additional 550 μl of ice-cold RIPA buffer supplemented with 10 mM sodium ascorbate, 10 mM NaN_3_, 5 mM Trolox, 1 mM phenylmethylsulfonyl fluoride, and cOmplete EDTA-free protease inhibitor cocktail, before being clarified by centrifugation at 13,000*g* for 30 min at 4°C. For the total lysate control, 2% of the clarified lysates were mixed with 4× Laemmli sample buffer and boiled for 8 min at 95°C. The remaining cell lysates were incubated with 40 μl of Pierce High-Capacity Streptavidin agarose beads (Sigma-Aldrich) on a rotating wheel overnight at 4°C. Samples were centrifuged at 250*g* for 2 min at 4°C, and agarose beads were washed five times with 1 ml of ice-cold RIPA buffer, once with 1 ml of ice-cold 1 M KCl, once with 1 ml of ice-cold 0.1 M Na_2_CO_3_, and once with 1 ml of ice-cold 2 M urea in 10 mM tris-HCl (pH 8.0). Biotinylated proteins were eluted from the agarose beads by boiling in 40 μl of 4× Laemmli sample buffer containing 2 mM biotin (Sigma-Aldrich) for 5 min at 95°C. Both total lysates and affinity-purified proteins were resolved using 4 to 20% Criterion TGX Precast Midi Protein Gel (Bio-Rad) and WB membrane probed with antibodies against LC3, NP, PCNT, DYNC1I1, HDAC6, and KIF5B (Abcam, #ab151558).

### Expansion microscopy

Cells were fixed as for the IF analyses and permeabilized with PBS containing 0.1% Triton X-100 and 3% BSA for 10 min at RT. Preparations were then blocked in 3% BSA-containing PBS for 30 min at RT and incubated with the primary antibodies against LAMP1 and M1, diluted 1:20 and 1:5 with 3% BSA-containing PBS, respectively, overnight at 4°C in a humidity chamber. Cells were washed three times with PBS and incubated with secondary antibodies conjugated to either Alexa Fluor 488 or Alexa Fluor 568 and diluted 1:50 in 3% BSA containing PBS at RT for 1 hour. Samples were postfixed with 0.25% glutaraldehyde (Sigma-Aldrich) and then washed three times with PBS. Subsequently, preparations were incubated with 30 μl of monomer solution [8.1% sodium acrylate (Merck Millipore), 2.66% acrylamide (Merck Millipore), 0.32% *N*,*N*′-methylenebisacrylamide (Merck Millipore), and 11.2% NaCl in PBS] at RT for 1 hour. Coverslips were placed upside down on a 52-μl drop of the gelation solution [monomer solution with freshly added 0.2% tetramethylenediamine (Merck Millipore) and 0.2% ammonium persulfate (Merck Millipore)] for 1 hour at RT. The coverslips were turned, with gel on the top, and incubated in digestion buffer [0.5% Triton X-100, 0.8 M guanidine-HCl (Merck Millipore), and proteinase K (8 U/ml; Merck Millipore) in TAE buffer (40 mM tris, (pH 8), 20 mM acetic acid, and 1 mM EDTA)] for 1 hour at 37°C. For each sample, a piece of the gel containing cells was cut out and placed in 3 ml of ice-cold water and incubated at 4°C overnight. The following day, gels were washed twice with 3 ml of water for 30 min at RT, with gentle rocking before labeling cell nuclei with Hoechst (2 mg/ml; Thermo Fisher Scientific) for 30 min at RT. Gels were washed again three times with water before imaging on an Andor BC43 spinning dish confocal microscope (Oxford Instruments, Abingdon, UK) equipped with a ×60 oil 1.42 numerical aperture objective and a complementary metal-oxide semiconductor camera with 1 by 1 binning, and controlled by the Fusion software from Andor. Images were acquired by collecting z-stacks of approximately 60 images at a thickness of 25 to 30 nm and deconvolved with the Fusion software (10 iterations). Images were analyzed with Imaris cell imaging software (Oxford Instruments). Images in the figures show representative examples of the imaged LEs.

The average distance between the luminal M1-positive VPs and the LAMP1-positive membrane of the LEs in expanded cells was determined in three biological replicates with at least 10 cells per replicate. For this, a line through the LAMP1-positive LEs and covering the shortest distance between the center of this compartment and the M1 punctum was drawn in the single z-stack slice in which both signals were best focused using the measurement point function in Imaris. Then, the distance between the maximum peak intensity of the M1 and LAMP1 fluorescent signals was manually extracted from the intensity plots. All the z-stacks of each collected image were inspected to find LAMP1-positive LEs containing at least one M1-positive VP, which were typically between 10 and 15 LEs per image (one to two cells per image). All the data were presented in violin graph using the GraphPad Prism 10 software (Dotmatics, Boston, MA). The control sample was used both for 3F and 5F.

### Purification and analysis of IAV VPs

Ten T125 flasks of 80% confluent MDCK cells were infected with IAV at an MOI of 0.01 for 72 hours before harvesting, combining, and clarifying the cell culture supernatants by centrifugation at 850*g* for 10 min at 4°C. The clarified supernatant was then layered over 20% sucrose in HNE buffer [20 mM Hepes, (pH 7.4), 150 mM NaCl, and 1 mM EDTA] to concentrate the virus by ultracentrifugation at 112,600*g* for 3 hours at 4°C in a SW32 rotor (Beckman Coulter, Fullerton, CA). The concentrated virus was lastly purified over a 20 to 60% sucrose gradient by ultracentrifugation at 112,600 *g* overnight at 4°C in a SW32 rotor. Twelve fractions were collected from the top to bottom, proteins were precipitated with 10% trichloroacetic acid (Sigma-Aldrich), and 50% of each precipitate was resolved by SDS-PAGE and silver stained as follow. Proteins were fixed in the gel with 50% methanol (Sigma-Aldrich), 10% acetic acid (Sigma-Aldrich), and 0.0185% formaldehyde (Sigma-Aldrich) for 1 hour at RT, followed by three washes with 50% ethanol (Sigma-Aldrich). The gel was then sensitized in 0.02% sodium thiosulfate (Sigma-Aldrich) for 1 min at RT, rinsed briefly with water three times, and incubated in 0.2% silver nitrate (Sigma-Aldrich) for 20 min at RT. The gel was then washed again three times with water, and the staining was developed using a mixture of 3% sodium carbonate (Sigma-Aldrich) and 0.05% formaldehyde (Sigma-Aldrich) at RT until protein bands became visible. The reaction was stopped by adding 5% acetic acid for 10 min at RT. The rest of the trichloroacetic acid precipitate of fraction 8, which the silver staining revealed to contain the IAV VPs, was also analyzed by WB using anti-NP, anti-LC3, and anti-ubiquitin antibodies (Enzo Life Sciences, Farmingdale, NY, #ENZ-ABS840-0100).

### Statistical analyses

Data represent the means of at least three independent biological replicates ± SD. Data were statistically analyzed using the Microsoft Excel (Microsoft, Redmond, WA) and the GraphPad Prism 10 software to determine significant differences (*P* < 0.05) between groups using the paired two-tailed Student’s *t* test and the two-way analysis of variance (ANOVA), followed by the Tukey’s post hoc test for multiple comparisons, respectively. The statistically significance (*P* < 0.05) were highlighted with the symbol *. Images in the figures show representative experiments.
