# Supplementary material for: Influenza A virus subverts the LC3-pericentrin dynein adaptor complex for host cytoplasm entry
Source: Sci Adv. 2025 Jun 11;11(24):eadu7602. doi: 10.1126/sciadv.adu7602 (PMC12154174; doi:10.1126/sciadv.adu7602)
Supplement: Supplementary file 1 — Figs. S1 to S10 Tables S1 to S3 [file sciadv.adu7602_sm.pdf]

Science Advances
AAAS

Supplementary Materials for

Influenza A virus subverts the LC3-pericentrin dynein adaptor complex for host cytoplasm entry

Yingying Cong *et al.*

Corresponding author: Fulvio Reggiori, f.m.reggiori@aias.au.dk

Sci. Adv. **11**, eadu7602 (2025)
DOI: 10.1126/sciadv.adu7602

This PDF file includes:

Figs. S1 to S10
Tables S1 to S3

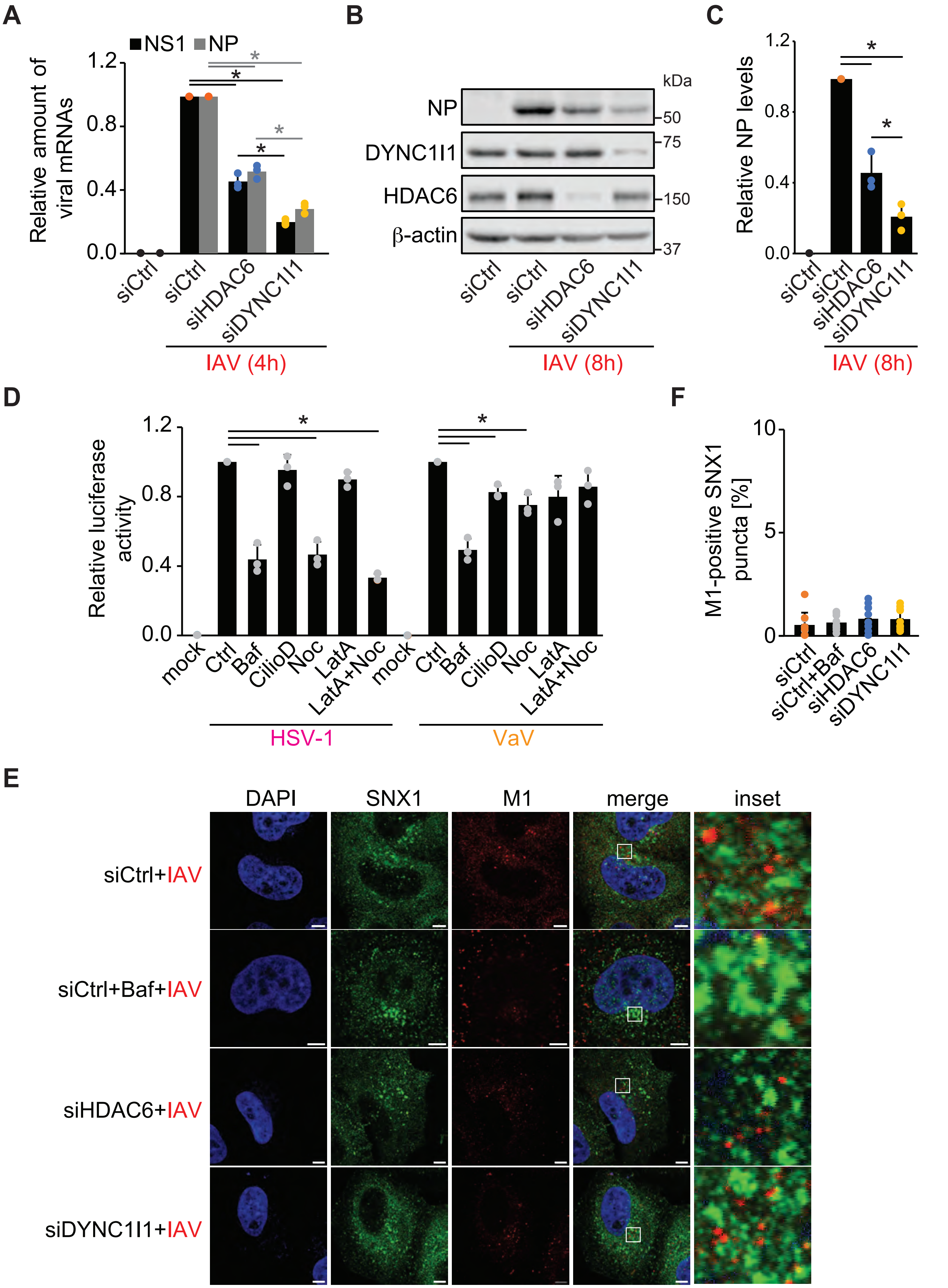

Fig. S1. HDAC6 is not the only dynein adaptor required for IAV cytoplasm entry. (**A**) HDAC6- or DYNC1I1-depleted sHeLa cells were infected with IAV at MOI 0.1 for 4 h, NP and NS1 mRNAs quantifed by RT-PCR, normalized to GAPDH mRNA and expressed relative to siCtrl cells. (**B**) In sHeLa cells treated as in Fig. 1A, IAV infection was examined by WB at 8 hpi using anti HDAC6, DYNC1I1, NP, and β-actin antibodies. (**C**) NP levels in panel B, relative to siCtrl cells. (**D**) sHeLa cells were infected with luc-HSV-1 or luc-VaV at MOI 1 for 6 h in the presence of 200 nM Baf, 10 μM CilioD, 1 μM Noc, 0.1 μM LatA or 1 μM Noc and 0.1 μM LatA simultaneously, before luciferase activity measurement, relative to the Ctrl cells. (**E**) sHeLa cells treated as in Fig. 1G were processed for IF with antibodies against M1 and SNX1. 200 nM Baf was used in the siCtrl condition. Insets highlight low colocalization between M1 and SNX1. Images were collected using a ZEISS LSM800 microscope. Scale bars, 5 μm. (**F**) Percentage of M1-positive SNX1 puncta in panel E. Error bars represent SDs (n=3, A, C, D; n=10, 50 cells counted per repeat). Asterisks indicate significant differences.

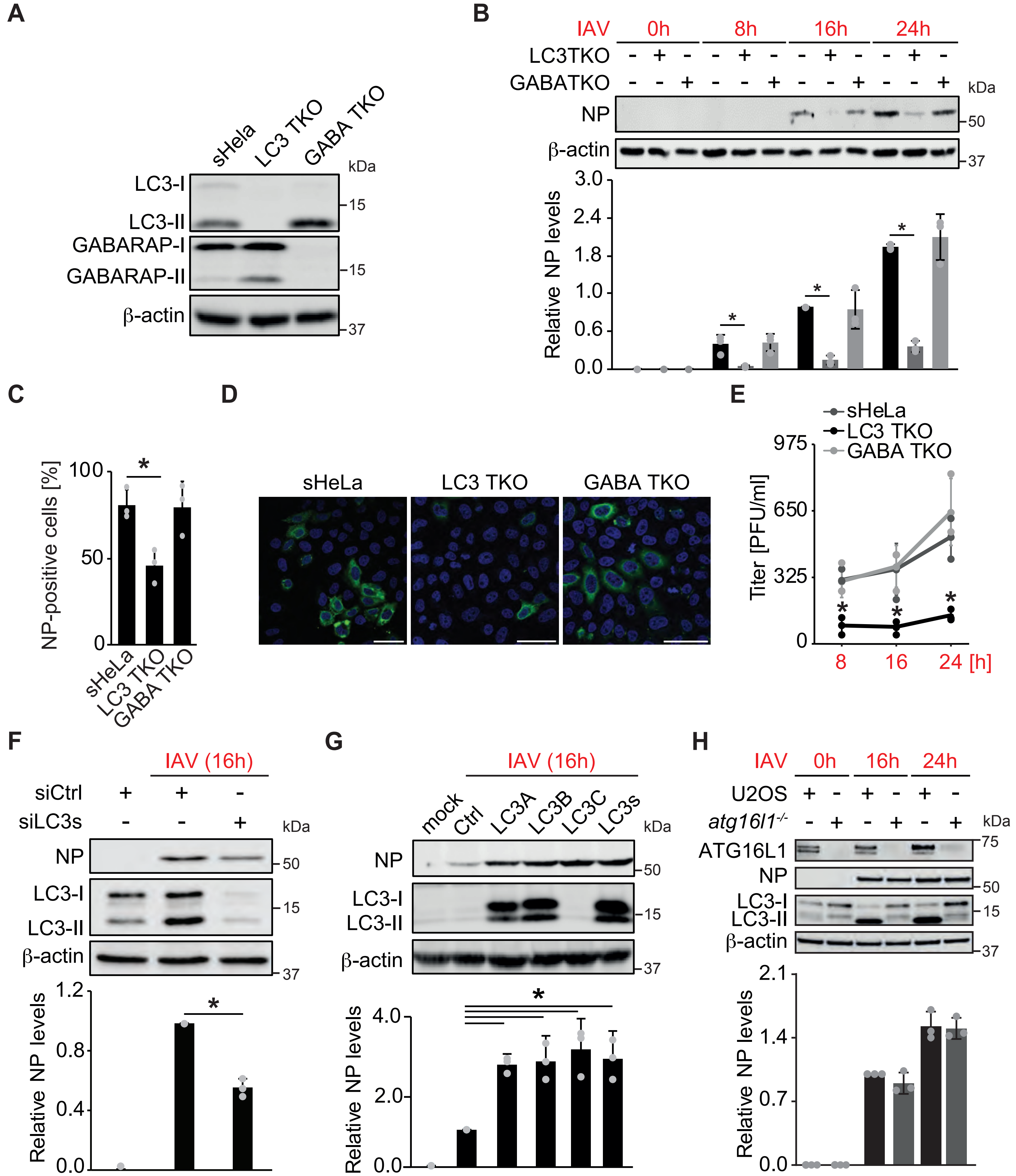

Fig. S2. LC3s but not GABARAPs are required for IAV infection. (**A**) sHeLa, LC3TKO and GABATKO cells were examined by WB with anti GABARAP, LC3 and β-actin antibodies. (**B**) Cells in panel A were infected with IAV at MOI 0.1 for 0, 8, 16 and 24 h before WB with anti NP and β-actin antibodies. NP levels are relative to sHeLa cells infected for 16 h. (**C**) Cells in panel A were infected with IAV at MOI 0.1 before IF with anti-NP antibodies at 12 hpi, to determine the percentage of infected cells. (**D**) Representative fluorescence images of the experiments in panel C collected using a DeltaVision microscope. Scale bars, 50 μm. (**E**) $TCID_{50}$/ml was determined in culture supernatants of panel B experiment. (**F**) A549 cells were transfected with siCtrl or of siRNAs targeting LC3s (siLC3s), infected with IAV at MOI 0.1 for 16 h and anlyzed by WB with anti NP, LC3 and β-actin antibodies. NP levels relative to infected cells transfected with siCtrl. (**G**) LC3TKO cells were transfected with pcDNA3.1-LC3A, pcDNA3.1-LC3B, pcDNA3.1-LC3C or these three plasmids in combination and infected with IAV at MOI 0.1 for 16 h before WB with anti NP, LC3 and β-actin antibodies. NP levels relative to LC3TKO cells transfected with the empty plasmid (Ctrl). The anti-LC3 antibody does not recognize LC3C but because the LC3TKO phenotype was complemented by transfection, showing that this protein is expressed. (**H**) IAV infected WT and *atg16L1*$^{-/-}$ U2OS cells at MOI 0.1 for 0, 16 and 24 h were analyzed by WB with anti ATG16L1, NP, LC3 and β-actin antibodies. NP levels relative to WT cells infected for 16 h. Error bars represent SDs (n=3 in B-H). Asterisks indicate significant differences.

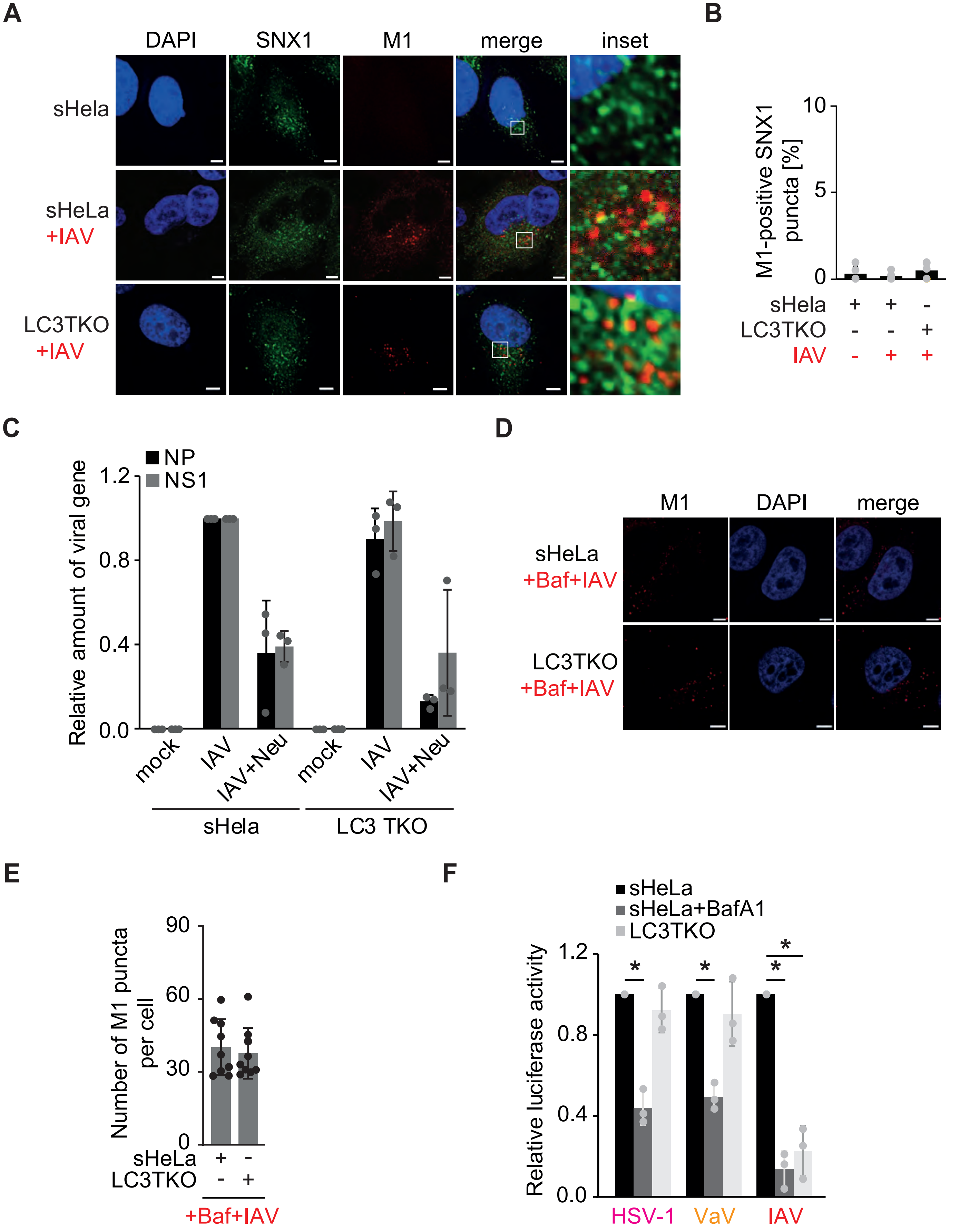

Fig. S3. IAV cytoplasm entry at LAMP1-positive compartments is impaired in the absence of LC3s. (**A**) IAV cytoplasm entry in sHeLa and LC3TKO cells was assayed as in Fig. 1G. Cells were then processed for IF with antibodies against IAV M1 and SNX1. Insets highlight the low colocalization between M1 and SNX1. Images were acquired using a ZEISS LSM800 microscope. Scale bars, 5 μm. (**B**) Percentage of M1-positive SNX1 puncta in panel A. (**C**) IAV virion attachment to the cell surface was assyed in sHeLa and LC3 TKO cells and cell surface-bound IAV was assessed by quantifying NP and NS1 genes by RT-PCR, and levels normalized to GAPDH and expressed relative to sHeLa incubated with IAV in the absence of Neu. (**D**) sHeLa and LC3TKO cells were assayed as in Fig 1G, but in presence of 200 nM Baf, and processed for IF with anti-M1 antibodies. Images were acquired using a ZEISS LSM800 microscope. Scale bars, 5 μm. (**E**) Quantification of the average number of M1 puncta per cell in panel D. (**F**) sHeLa and LC3TKO cells were infected with luc-HSV-1 or luc-VaV at MOI 1 for 6 h or luc-IAV at MOI 1 for 16 h before measuring the luciferase activity, expressed relative to the sHeLa cells in each condition. Error bars represent SDs (n=3 in B and n=4 in E, 50 cells per repeat; n=3 in F). Asterisks indicate significant differences.

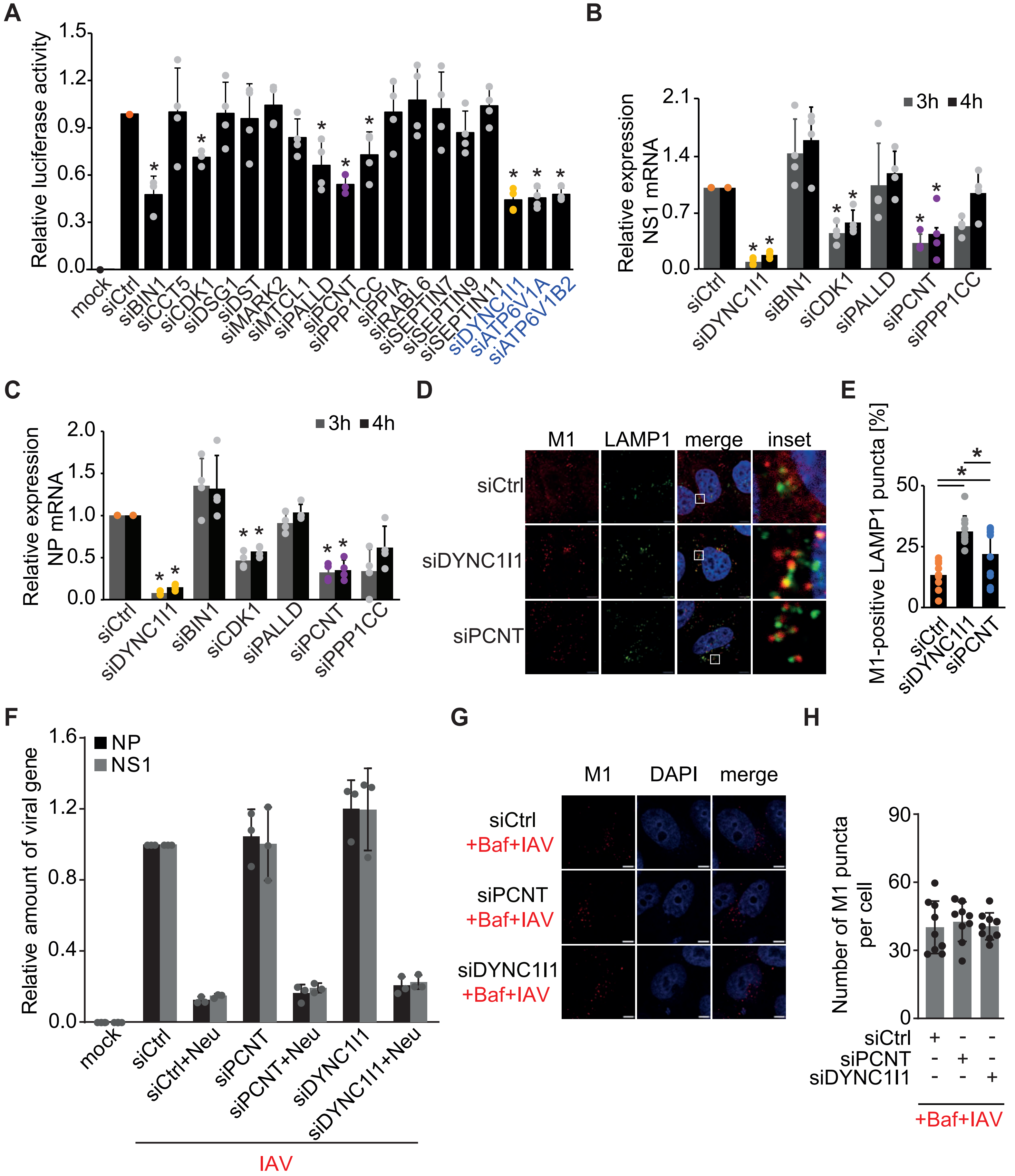

Fig. S4. PCNT is important for IAV cytoplasm entry. (**A**) sHeLa cells in which the indicated genes (table S2) were depleted were infected with the WSN-luc IAV strain at MOI 1 before measuring the luciferase activity at 16 hpi, and expressing relative to cells treated with siCtrl. (**B** and **C**) DYNC1I1-, BIN1-, CDK1-, PALLD-, PCNT- or PPP1CC-depleted sHeLa cells were infected with IAV at MOI 0.1. NS1 (B) and NP (C) mRNA levels at 3 and 4 hpi were quantified by RT-PCR, normalized to GAPDH and expressed relative to siCtrl. (**D**) sHeLa transfected with siCtrl, siPCNT or siDYNC1I1 were infected and processed for IF as in Fig. 1G with antibodies against M1 and LAMP1. Insets highlight the M1-positive LAMP1 puncta. Scale bars, 5 μm. (**E**) Percentage of M1-positive LAMP1 puncta in panel D. (**F**) IAV virion attachment to the cell surface was assayed in sHeLa cells treated with indicated siRNA, and cell surface-bound IAV was assessed by quantifying NP and NS1 genes by RT-PCR, and levels normalized to GAPDH and expressed relative to infected sHeLa cells without Neu. (**G**) sHeLa cells treated with indicated siRNA were treated as in Fig. 1G but in presence of 200 nM Baf, and processed for IF with anti-M1 antibodies. Images were acquired using a ZEISS LSM800 microscope. Scale bars, 5 μm. (**H**) Number of M1 puncta per cell in panel G. Error bars represent SDs (Error bars represent SDs (n=4 in A, B, C; n=10 in D or n=4 in H, 50 cells counted per repeat).

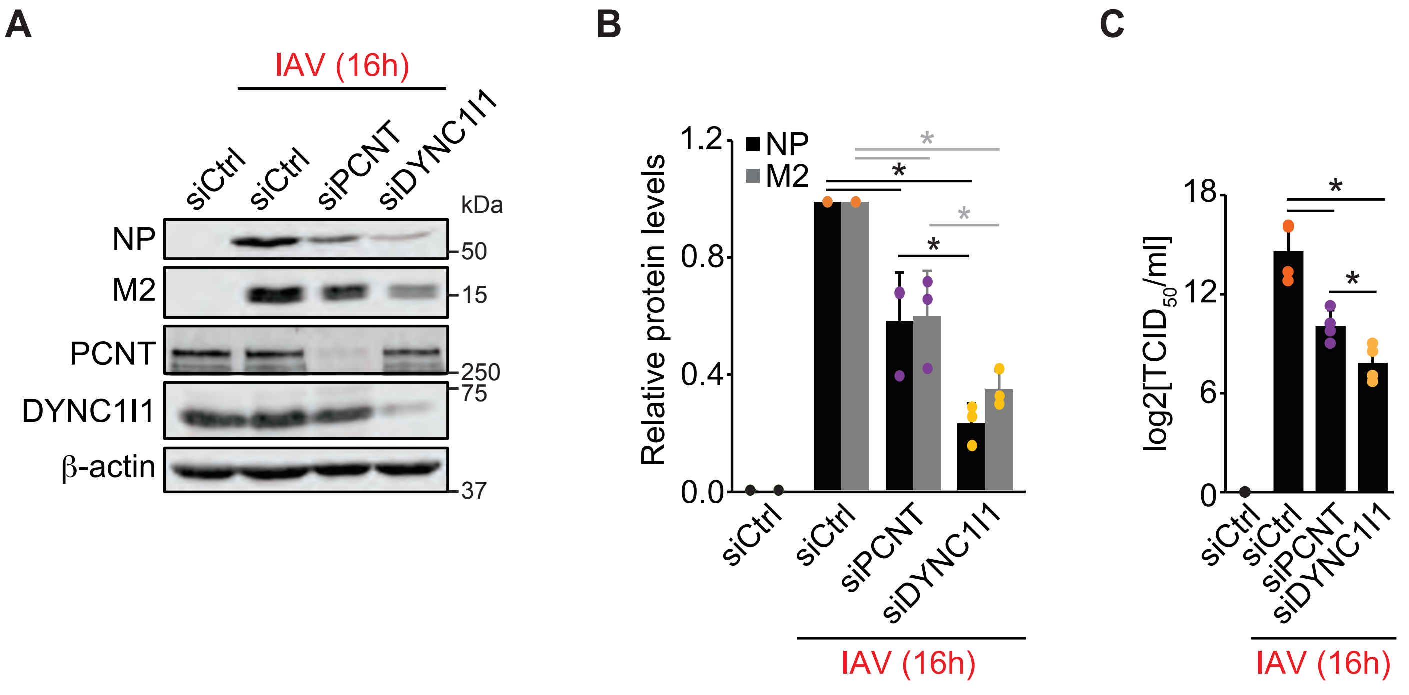

Fig. S5. PCNT is important for IAV viral cycle. (**A**) Cells prepared as in Fig. 5G were infected with IAV at MOI 0.1 for 16 h and cell extracts analyzed by WB with anti NP, M2, PCNT, DYNC1I1 and -actin antibodies. (**B**) Quantification of NP and M2 levels in panel A, expressed relative to the infected cells treated with siCtrl. (**C**) Cell culture supernatants from the experiment in panel A were collected to assess the production of infectious virus progeny by determining $TCID_{50}$/ml of supernatants. Error bars represent SDs (n=3 in B, C). Asterisks indicate significant differences.

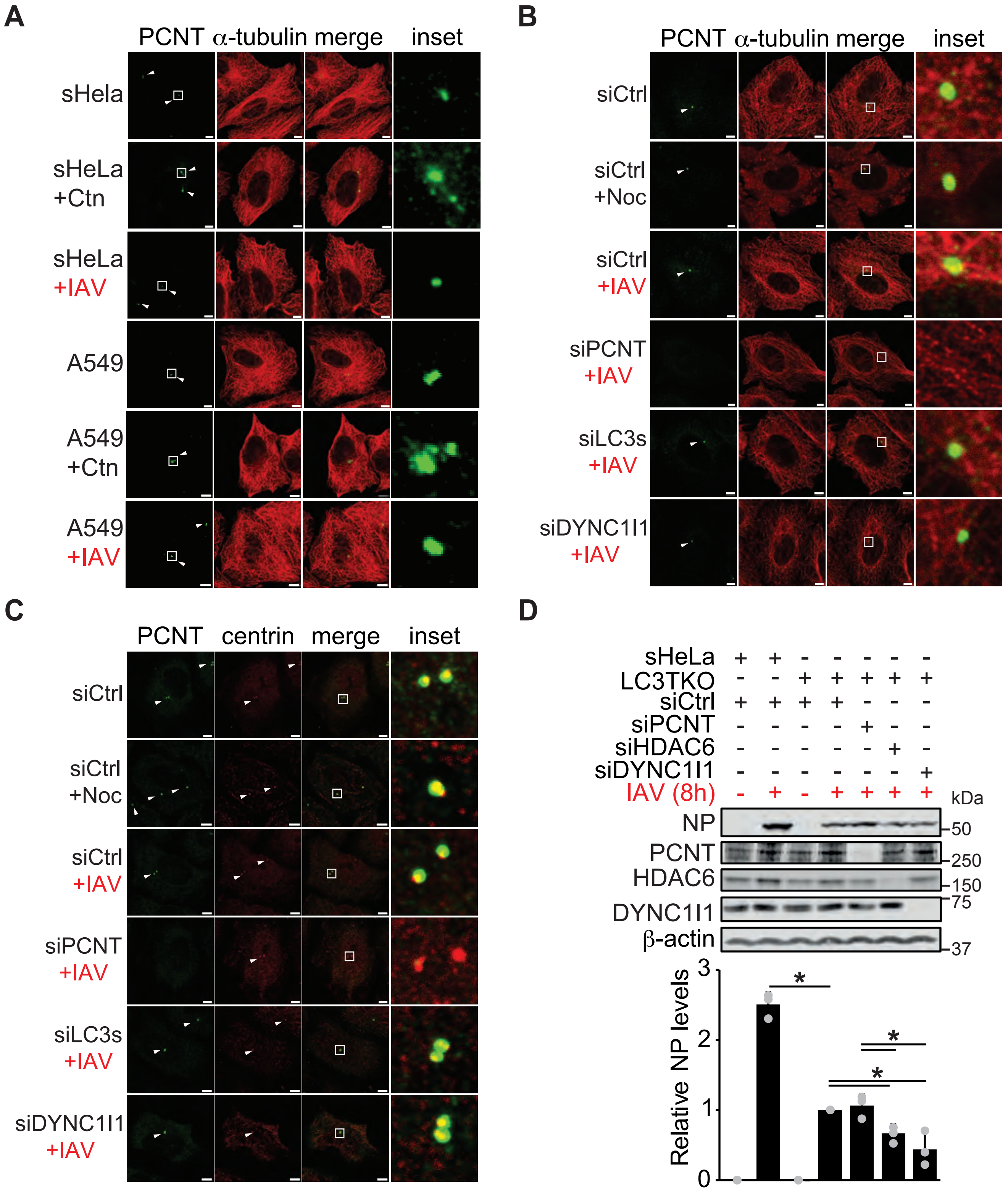

Fig. S6. IAV virus exploits the host centrosome assembly machinery for cytoplasm entry. (**A**) sHeLa or A549 cells were treated with 100 nM Ctn for 8 h or inoculated with IAV at MOI 0.1 for 8 h and processed for IF with antibodies against PCNT and α-tubulin. The PCNT puncta are highlighted with white arrowheads and their organization with insets. Images were collected using a ZEISS LSM800 microscope. Scale bars, 5 μm. (**B**) sHeLa cells were depleted of PCNT, LC3s or DYNC1I1 and infected with IAV at MOI 0.1 for 8 h, before being processed for IF with antibodies against PCNT and α-tubulin. While PCNT staining was used to visualize its silencing and centrosomes, treatment with 1 μM Noc served as a control to disrupt MTs. The PCNT puncta are highlighted with white arrowheads and the organization of MTs with insets. (C) Cells in panel B were also processed for IF with anti-PCNT and anti-centrin antibodies. Both PCNT and centrin puncta are indicated with white arrowheads and colocalization between these two proteins is highlighted with insets. Images were acquired using a ZEISS LSM800 microscope. Scale bars, 5 μm. (D) sHeLa cells treated with indicated siRNA, were infected with IAV at MOI 0.1 for 8 h and cell extracts examined by WB with anti NP, PCNT, HDAC6, DYNC1I1 and β-actin antibodies and NP levels expressed relative to infected siCtrl LC3TKO cells. Error bars represent SDs (n=3). Asterisks indicated significant differences.

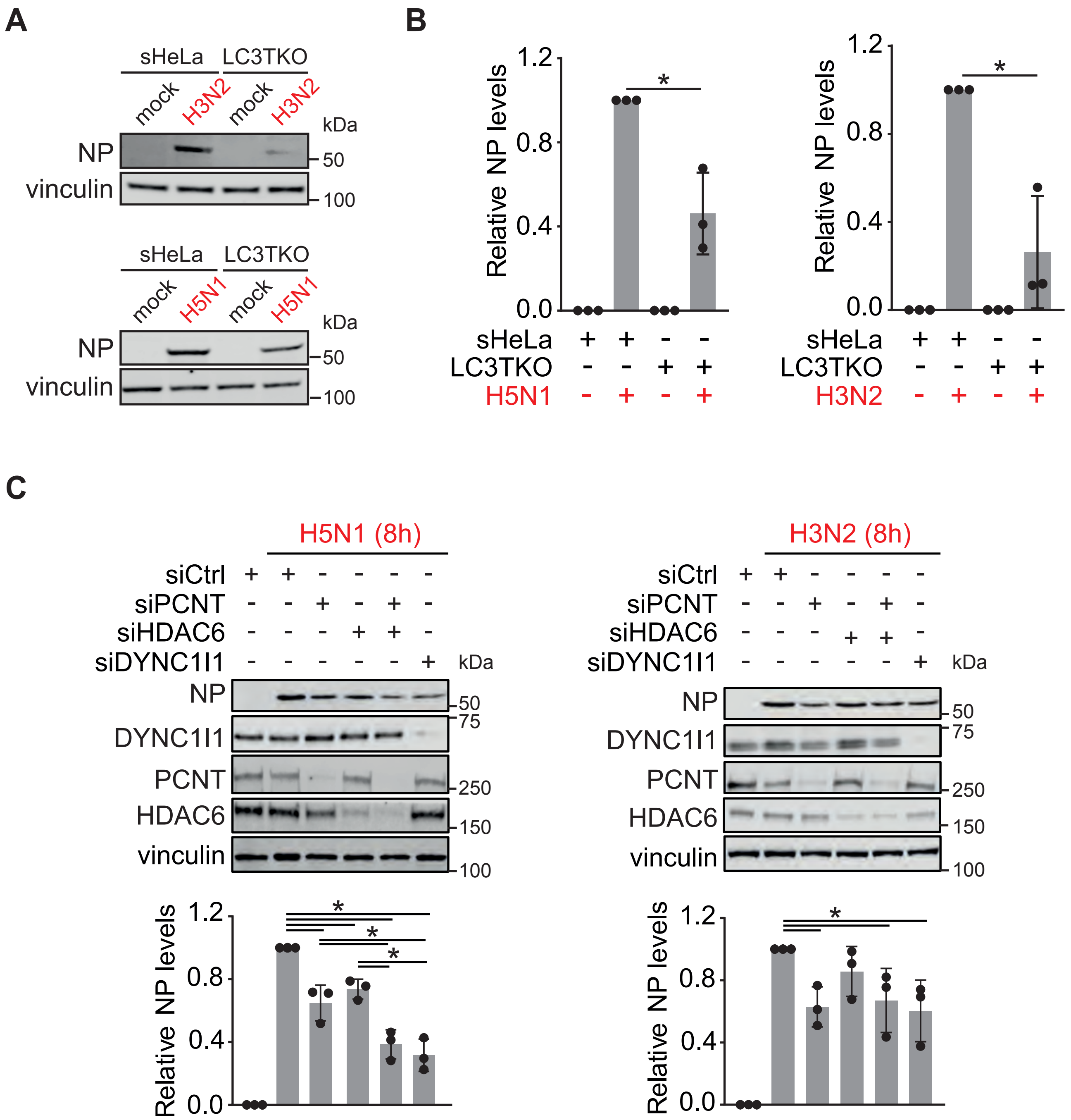

Fig. S7. Relevance of LC3-PCNT and HDAC6 in H3N2 and H5N1 subtype infections. (**A**) sHeLa and LC3TKO cells were infected with either the A/NIBRG14/H5N1 or the A/Perth/H3N2 strain at MOI 1 and 5, respectively, for 8 h before WB using anti-NP and -vinculin antibodies. (**B**) NP levels in the experiments in panel A expressed relative to infected sHeLa cells. (**C**) DYNC1I1, PCNT, HDAC6 or PCNT and HDAC6 in combination were knocked down in sHeLa cells before infection with the A/NIBRG14/H5N1 or A/Perth/H3N2 strain at MOI 1 and 5, respectively, for 8 h. Cell lysates were analyzed by WB analysis using antibodies against NP, PCNT, HDAC6, DYNC1I1 and vinculin. NP levels are expressed relative to infected siCtrl cells (lower panel). Error bars represent the SDs (n=3 in B, C). Asterisks indicate significant differences.

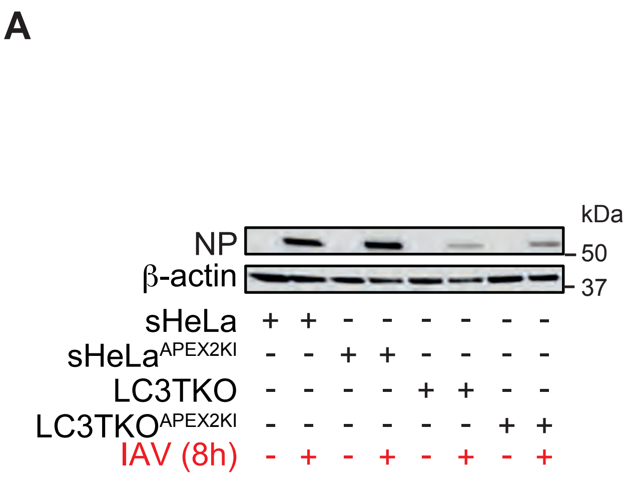

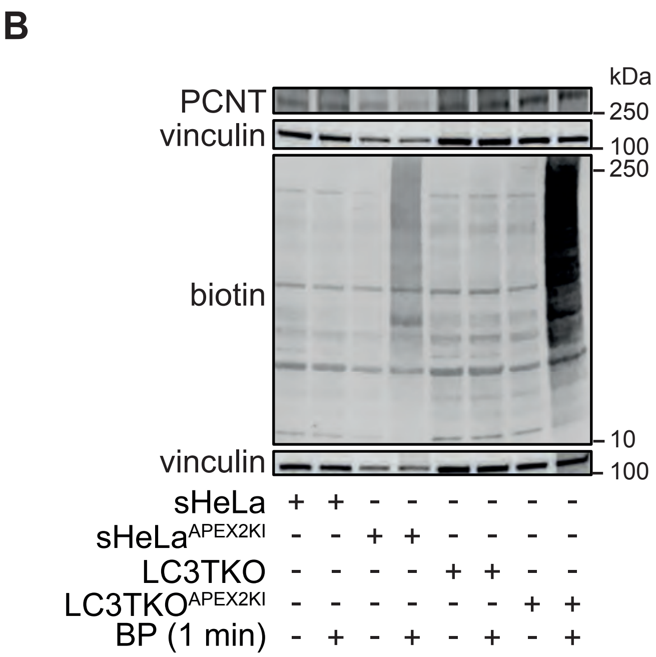

S8. The LC3-PCNT module connect the vRNPs with dynein. (**A**) sHeLa, sHeLa^{APEX2KI}, LC3TKO and LC3TKO^{APEX2KI} cells were infected with IAV at MOI 0.1 for 8 h before analyzing cell extracts by WB with anti NP and β-actin antibodies. (**B**) sHeLa, sHeLa^{APEX2KI}, LC3TKO and LC3TKO^{APEX2KI} cells grown in DMEM medium supplemented with 10% FCS, were incubated with 500 μM BP and 1 mM H_2O_2 for 30 min and 1 min, respectively, before examining the cell extracts by WB with anti PCNT, biotin and vinculin antibodies. Asterisks indicate significant differences.

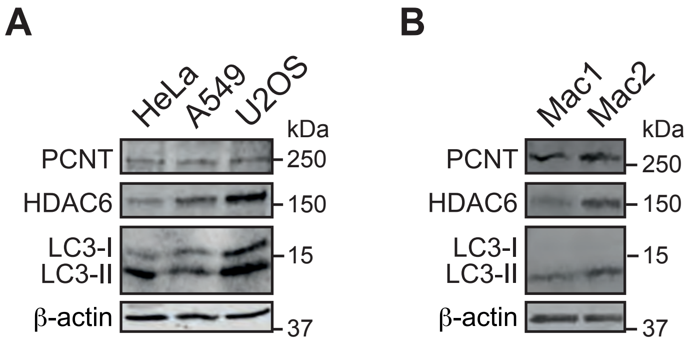

Fig. S9. Expression of PCNT, LC3 and HDAC6 in different cell types. (**A**) Cell lysates from HeLa, A549 and U2OS cells were analyzed by WB using antibodies against PCNT, HDAC6, LC3 and β-actin antibodies. (**B**) Cell lysates from human monocyte-derived macrophages were examined using antibodies against PCNT, HDAC6, LC3 and β-actin. Mac1 represent standard macrophages induced by treating human monocytes with 10 ng/ml of M-CSF for 7 d. Mac2 resemble alveolar-like macrophages and they are induced by treating human monocytes with 10 ng/ml GM-CSF, 5 ng/ml IL-4 and 5 ng/ml TGF-β1 for 7 d.

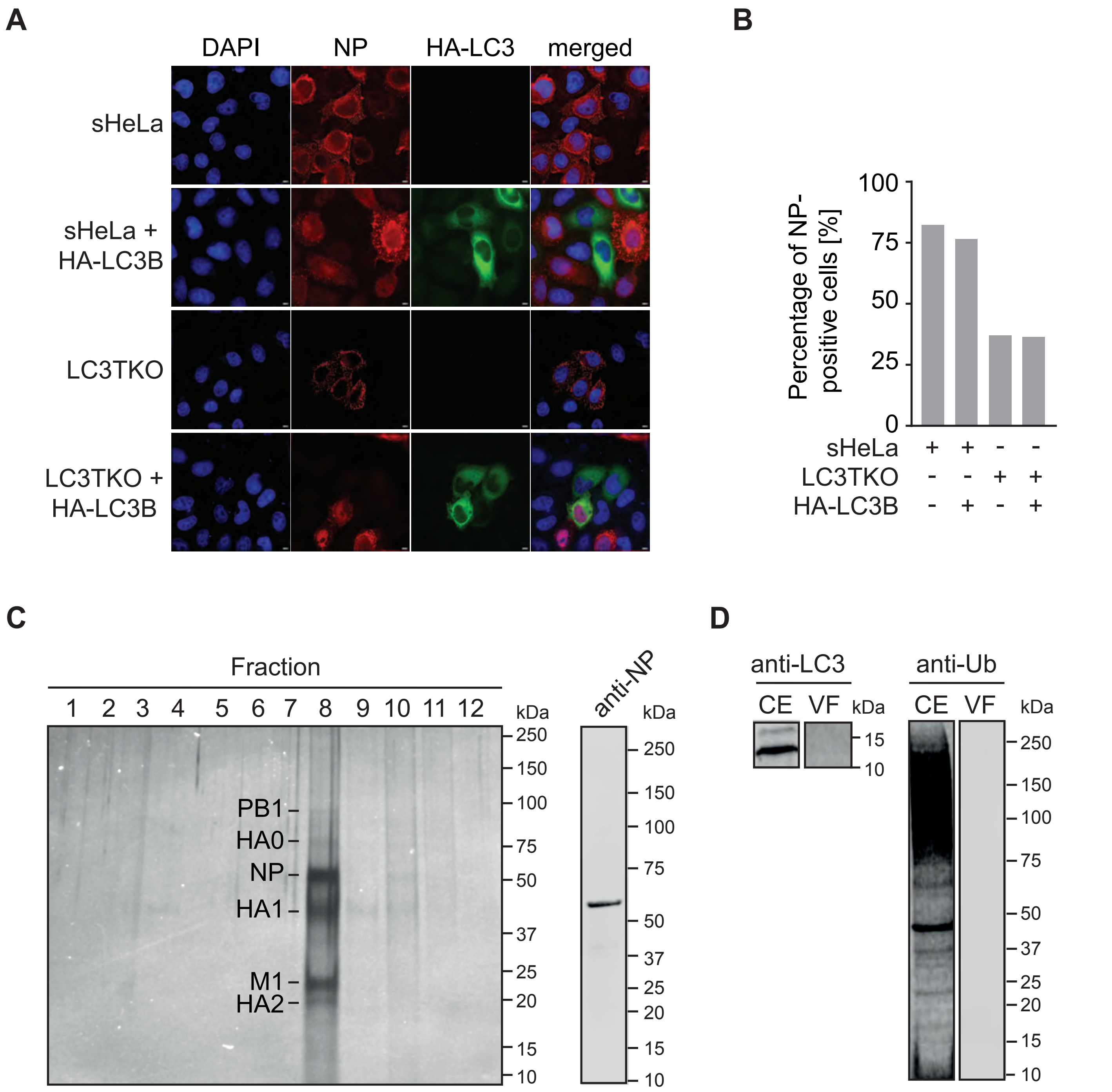

Fig. S10. Tagged LC3s LC3s are not associated with IAV virion. (**A**) sHeLa and LC3TKO cells were transfected or not with the pCDNA3.1-3xHA-LC3B plasmid for 48 h and then infected with IAV at a MOI of 0.1 before being processed for IF at 16 hpi. Cells were stained with anti-NP and anti-HA antibodies. Size bar, 5 µm. (**B**) Percentage of the NP-positive, infected cells in the experiment shown in panel A (n=1). (**C**) MDCK cells were infected with IAV at MOI 0.01 for 72 h before harvesting the cell culture supernatants and purify viral particles initially enriched by ultracentrifugation on a 10-60% sucrose gradient. Twelve fractions were collected and precipitated with 10% tri-chloroacetic acid. 50% of the precipitated proteins were resolved by SDS-PAGE and the gel was then silver stained (left). The presence of IAV viral particles in fraction 8 was confirmed by WB using anti-NP antibodies (right). (**D**) TFraction 8 (viral fraction, VF) was also analysed by WB using antibodies against LC3s and ubiquitin. Cell extracts (CE) from MDCK cells were used as a control to show the presence of the examined proteins in MDCK cells and that the used antibodies work.

Table S1: List of proteins that interact with LC3s upon IAV cell entry in $atg7^{-/-}$ U2OS cells identified by mass spectrometry.

		Exp1						Exp2						Exp3					
Protein_ID	Protein Description	Score	#Spec	#Pep	#Uniq	%Pep	%Cov	Score	#Spec	#Pep	#Uniq	%Pep	%Cov	Score	#Spec	#Pep	#Uniq	%Pep	%Cov
PB2_I34A1	Polymerase basic protein 2 OS=Influenza A virus (strain A/Puerto Rico/8/1934 H1N1) OX=211044 GN=PB2 PE=1 SV=2	106,15	3	3	3	2,8	4,08							50,56	1	1	1	0,93	1,05
RDRP_I34A1	RNA-directed RNA polymerase catalytic subunit OS=Influenza A virus (strain A/Puerto Rico/8/1934 H1N1) OX=211044 GN=PB1 PE=1 SV=2	105,57	3	3	3	2,97	3,7							71,45	1	1	1	1,11	1,54
A_I34A1	Polymerase acidic protein OS=Influenza A virus (strain A/Puerto Rico/8/1934 H1N1) OX=211044 GN=PA PE=1 SV=2	74,09	1	1	1	1,11	1,54							93,58	1	1	1	1,79	1,77
HEMA_I34A1	Hemagglutinin OS=Influenza A virus (strain A/Puerto Rico/8/1934 H1N1) OX=211044 GN=HA PE=1 SV=2							116,58	3	2	2	3,57	3,36	359,15	128	30	30	43,48	58,03
NCAP_I34A1	Nucleoprotein OS=Influenza A virus (strain A/Puerto Rico/8/1934 H1N1) OX=211044 GN=NP PE=1 SV=2	430,93	154	34	34	49,28	59,64	394,11	78	29	29	42,03	56,63	317,98	97	20	20	68,97	82,94
M1_I34A1	Matrix protein 1 OS=Influenza A virus (strain A/Puerto Rico/8/1934 H1N1) OX=211044 GN=M PE=1 SV=1	393,24	94	22	22	75,86	85,71	360,16	55	18	18	62,07	69,05	50,56	1	1	1	1,37	1,57
A0A075B6S5	Immunoglobulin kappa variable 1-27 OS=Homo sapiens OX=9606 GN=IGKV1-27 PE=3 SV=1	40,73	1	1	1	11,11	13,68							49,54	1	1	1	11,11	13,68
A0A0B4J2D9	Immunoglobulin kappa variable 1D-13 OS=Homo sapiens OX=9606 GN=IGKV1D-13 PE=3 SV=1	52,73	1	1	1	12,5	13,68	76,14	3	1	1	12,5	13,68	61,02	1	1	1	12,5	13,68
A0A0C4DH67	Immunoglobulin kappa variable 1-8 OS=Homo sapiens OX=9606 GN=IGKV1-8 PE=3 SV=1	40,73	1	1	1	11,11	13,91							49,54	1	1	1	11,11	13,91
A0A0C4DH69	Immunoglobulin kappa variable 1-9 OS=Homo sapiens OX=9606 GN=IGKV1-9 PE=3 SV=1	40,73	1	1	1	12,5	13,68							49,54	1	1	1	12,5	13,68
A0M8Q6	Immunoglobulin lambda constant 7 OS=Homo sapiens OX=9606 GN=IGLC7 PE=1 SV=3	71,32	3	1	1	9,09	7,55	58,78	2	1	1	9,09	7,55	61,58	2	1	1	9,09	7,55

A6NCE7	Microtubule-associated proteins 1A/1B light chain 3 beta 2 OS=Homo sapiens OX=9606 GN=MAP1LC3B2 PE=2 SV=1	182,47	10	7	7	35	38,4	162,65	4	4	4	20	30,4	100,8	3	2	2	10	19,2
O00159	Unconventional myosin-Ic OS=Homo sapiens OX=9606 GN=MYO1C PE=1 SV=4							124,68	3	3	3	2,01	2,63	249,87	18	17	17	11,41	20,6
O00303	Eukaryotic translation initiation factor 3 subunit F OS=Homo sapiens OX=9606 GN=EIF3F PE=1 SV=1	145,96	7	3	3	13,04	11,2	115,85	2	1	1	4,35	5,32	85,09	2	1	1	4,35	5,32
O00425	Insulin-like growth factor 2 mRNA-binding protein 3 OS=Homo sapiens OX=9606 GN=IGF2BP3 PE=1 SV=2							56,51	1	1	1	1,41	2,42	49,81	1	1	1	1,41	2,42
O00499	Myc box-dependent-interacting protein 1 OS=Homo sapiens OX=9606 GN=BIN1 PE=1 SV=1	233,51	8	7	7	11,86	19,06	121,35	2	2	2	3,39	6,24						
O00571	ATP-dependent RNA helicase DDX3X OS=Homo sapiens OX=9606 GN=DDX3X PE=1 SV=3	253,26	14	11	11	12,94	18,13	241,58	11	8	8	9,41	15,26	202,48	8	7	7	8,24	13,44
O14556	Glyceraldehyde-3-phosphate dehydrogenase, testis-specific OS=Homo sapiens OX=9606 GN=GAPDHS PE=1 SV=2	91,32	2	2	1	4,65	4,41	70,07	1	1	1	2,33	2,7						
O14950	Myosin regulatory light chain 12B OS=Homo sapiens OX=9606 GN=MYL12B PE=1 SV=2	69,12	1	1	1	4,35	5,81							64,81	1	1	1	4,35	5,81
O14980	Exportin-1 OS=Homo sapiens OX=9606 GN=XPO1 PE=1 SV=1	146,26	5	3	3	2,97	4,58							66,1	1	1	1	0,99	1,12
O15042	U2 snRNP-associated SURP motif-containing protein OS=Homo sapiens OX=9606 GN=U2SURP PE=1 SV=2	112,49	4	4	4	2,21	7,97							72,58	2	1	1	0,55	1,65
O15156	Zinc finger and BTB domain-containing protein 7B OS=Homo sapiens OX=9606 GN=ZBTB7B PE=1 SV=2	115,29	3	2	2	4,26	3,71	69,77	1	1	1	2,13	1,86	61,26	1	1	1	2,13	1,86
O15372	Eukaryotic translation initiation factor 3 subunit H OS=Homo sapiens OX=9606 GN=EIF3H PE=1 SV=1	123,39	3	3	3	7,69	15,91	91,43	1	1	1	2,56	5,4	78,46	1	1	1	2,56	5,4
O43143	Pre-mRNA-splicing factor ATP-dependent RNA helicase DHX15 OS=Homo sapiens OX=9606 GN=DHX15 PE=1 SV=2	160,63	4	4	4	3,81	9,06							69,21	1	1	1	0,95	1,64
O43175	D-3-phosphoglycerate dehydrogenase OS=Homo sapiens OX=9606 GN=PHGDH PE=1 SV=4	252,64	9	9	9	19,15	26,08	54,16	1	1	1	2,13	1,5	97,31	2	2	2	4,26	5,07

O43390	Heterogeneous nuclear ribonucleoprotein R OS=Homo sapiens OX=9606 GN=HNRNPR PE=1 SV=1	183,24	5	5	3	5,49	9,48	129,94	2	2	2	2,2	3,95						
O43399	Tumor protein D54 OS=Homo sapiens OX=9606 GN=TPD52L2 PE=1 SV=2	387,58	56	18	18	66,67	59,71	296,19	20	11	11	40,74	55,34	285,19	27	12	12	44,44	50,49
O43683	Mitotic checkpoint serine/threonine-protein kinase BUB1 OS=Homo sapiens OX=9606 GN=BUB1 PE=1 SV=1	365,1	32	19	19	18,27	27,83	296,15	14	10	10	9,62	15,85	278,25	16	13	13	12,5	18,99
O43684	Mitotic checkpoint protein BUB3 OS=Homo sapiens OX=9606 GN=BUB3	153,34	3	3	3	8,82	10,98	82,38	1	1	1	2,94	4,27	155,76	3	3	3	8,82	11,28
O43707	Alpha-actinin-4 OS=Homo sapiens OX=9606 GN=ACTN4 PE=1 SV=2							128,21	4	3	1	2,88	3,4	167,72	5	5	2	4,81	6,48
O43795	Unconventional myosin-Ib OS=Homo sapiens OX=9606 GN=MYO1B PE=1 SV=3	51,17	1	1	1	0,56	1,85							125,6	3	3	3	1,67	3,61
O43809	Cleavage and polyadenylation specificity factor subunit 5 OS=Homo sapiens OX=9606 GN=NUDT21 PE=1 SV=1	39,29	1	1	1	4	12,78	60,52	1	1	1	4	12,78	53,46	1	1	1	4	12,78
O60506	Heterogeneous nuclear ribonucleoprotein Q OS=Homo sapiens OX=9606 GN=SYNCRIP PE=1 SV=2	140,78	3	3	1	3,33	5,46							107,39	2	2	2	2,22	3,37
O60814	Histone H2B type 1-K OS=Homo sapiens OX=9606 GN=H2BC12 PE=1 SV=3	240,51	20	9	3	32,14	51,59	239,22	15	6	3	21,43	32,54	193,26	19	7	2	25	42,86
O60884	DnaJ homolog subfamily A member 2 OS=Homo sapiens OX=9606 GN=DNAJA2 PE=1 SV=1							61,18	1	1	1	1,79	3,16	103,67	2	2	2	3,57	14,81
O75152	Zinc finger CCCH domain-containing protein 11A OS=Homo sapiens OX=9606 GN=ZC3H11A PE=1 SV=3	312,35	22	18	18	14,63	28,02	230,34	9	7	7	5,69	12,96	228,76	13	10	10	8,13	15,93
O75427	Leucine-rich repeat and calponin homology domain-containing protein 4 OS=Homo sapiens OX=9606 GN=LRCH4 PE=1 SV=2	208,94	7	7	7	10	13,18							50,57	1	1	1	1,43	1,32
O75449	Katanin p60 ATPase-containing subunit A1 OS=Homo sapiens OX=9606 GN=KATNA1 PE=1 SV=1							66,9	1	1	1	1,39	2,44	57,87	1	1	1	1,39	2,44
O75489	NADH dehydrogenase [ubiquinone] iron-sulfur protein 3, mitochondrial OS=Homo sapiens OX=9606 GN=NDUFS3 PE=1 SV=1	65,22	2	1	1	3,23	5,3	56,49	1	1	1	3,23	5,3						
O75494	Serine/arginine-rich splicing factor 10 OS=Homo sapiens OX=9606 GN=SRSF10 PE=1 SV=1	92,63	2	2	2	2,9	11,07	54,82	1	1	1	1,45	4,96						

O75533	Splicing factor 3B subunit 1 OS=Homo sapiens OX=9606 GN=SF3B1 PE=1 SV=3	222,41	13	9	9	5,77	8,13	106,63	2	2	2	1,28	3,22	125,53	3	2	2	1,28	2,3
O75955	Flotillin-1 OS=Homo sapiens OX=9606 GN=FLOT1 PE=1 SV=3							188,2	5	5	5	8,77	23,89	267,94	16	15	15	26,32	50,59
O75964	ATP synthase subunit g, mitochondrial OS=Homo sapiens OX=9606 GN=ATP5MG PE=1 SV=3	107,62	1	1	1	8,33	14,56							84,4	1	1	1	8,33	14,56
O76031	ATP-dependent Clp protease ATP-binding subunit clpX-like, mitochondrial OS=Homo sapiens OX=9606 GN=CLPX	70,4	1	1	1	1,2	2,05	85,15	1	1	1	1,2	2,05	77,06	1	1	1	1,2	2,05
O94915	Protein furry homolog-like OS=Homo sapiens OX=9606 GN=FRYL PE=1 SV=2	311,39	22	21	21	7,64	9,56	135,77	3	3	3	1,09	1,39	248,8	16	15	15	5,45	6,87
O95071	E3 ubiquitin-protein ligase UBR5 OS=Homo sapiens OX=9606 GN=UBR5	118,17	5	4	4	1,34	3,18	106,38	1	1	1	0,34	0,86	82,73	2	1	1	0,34	0,89
O95104	SR-related and CTD-associated factor 4 OS=Homo sapiens OX=9606 GN=SCAF4 PE=1 SV=3	289,52	15	12	12	9,6	17,52	181,67	2	2	2	1,6	4,88	223,01	9	7	7	5,6	11,68
O95425	Supervillin OS=Homo sapiens OX=9606 GN=SVIL PE=1 SV=2							88,23	1	1	1	0,34	0,86	155,97	4	3	3	1,01	1,49
O95613	Pericentrin OS=Homo sapiens OX=9606 GN=PCNT PE=1 SV=4	245,17	14	12	12	2,67	4,59							85,88	2	2	2	0,45	0,78
O95793	Double-stranded RNA-binding protein Staufen homolog 1 OS=Homo sapiens OX=9606 GN=STAU1 PE=1 SV=2	84,02	2	2	2	2,99	4,85							73,47	1	1	1	1,49	2,25
P00338	L-lactate dehydrogenase A chain OS=Homo sapiens OX=9606 GN=LDHA	87,33	2	2	2	5	6,02	131,88	3	3	2	7,5	9,04	95,11	2	2	1	5	6,63
P01593	Immunoglobulin kappa variable 1D-33 OS=Homo sapiens OX=9606 GN=IGKV1D-33 PE=1 SV=2	105,88	6	1	1	14,29	13,68	95,48	4	1	1	14,29	13,68	91,5	4	1	1	14,29	13,68
P01594	Immunoglobulin kappa variable 1-33 OS=Homo sapiens OX=9606 GN=IGKV1-33 PE=1 SV=2	105,88	6	1	1	14,29	13,68	95,48	4	1	1	14,29	13,68	91,5	4	1	1	14,29	13,68
P01857	Immunoglobulin heavy constant gamma 1 OS=Homo sapiens OX=9606 GN=IGHG1 PE=1 SV=1	139,55	77	3	3	9,68	5,76	96,81	7	2	2	6,45	4,55	107,95	28	3	3	9,68	5,76
P01860	Immunoglobulin heavy constant gamma 3 OS=Homo sapiens OX=9606 GN=IGHG3 PE=1 SV=2	139,55	77	3	3	8,11	5,04	96,81	7	2	2	5,41	3,98	107,95	28	3	3	8,11	5,04
P02511	Alpha-crystallin B chain OS=Homo sapiens OX=9606 GN=CRYAB PE=1 SV=2	217,08	8	7	7	33,33	45,71	172	7	6	6	28,57	44,57	171,92	6	6	6	28,57	36,57

P02533	Keratin, type I cytoskeletal 14 OS=Homo sapiens OX=9606 GN=KRT14 PE=1 SV=4	364,01	39	24	9	43,64	57,63	346,77	34	23	10	41,82	53,6	331,49	61	29	10	52,73	60,38
P02538	Keratin, type II cytoskeletal 6A OS=Homo sapiens OX=9606 GN=KRT6A PE=1 SV=3	335,88	38	24	1	35,82	35,99	328,41	32	21	2	31,34	41,49	294,46	43	24	2	35,82	39,36
P02545	Prelamin-A/C OS=Homo sapiens OX=9606 GN=LMNA PE=1 SV=1	246,62	11	11	11	10,48	19,73	210,9	5	5	5	4,76	10,54	238,08	13	12	11	11,43	22,89
P02768	Albumin OS=Homo sapiens OX=9606 GN=ALB PE=1 SV=2	191,3	12	7	7	8,43	11,33	187,2	8	5	5	6,02	9,03	147,68	6	4	4	4,82	7,06
P04264	Keratin, type II cytoskeletal 1 OS=Homo sapiens OX=9606 GN=KRT1 PE=1 SV=6	512,78	156	46	40	69,7	68,17	490,05	137	42	37	63,64	67,24	440,66	228	47	41	71,21	67,55
P04406	Glyceraldehyde-3-phosphate dehydrogenase OS=Homo sapiens OX=9606 GN=GAPDH PE=1 SV=3	162,49	5	4	3	11,11	13,73	207,82	7	5	5	13,89	26,87	157,38	4	4	4	11,11	17,31
P04792	Heat shock protein beta-1 OS=Homo sapiens OX=9606 GN=HSPB1 PE=1 SV=2	342,16	29	13	13	59,09	90,73	309,75	18	12	12	54,55	86,83	291,05	23	14	14	63,64	86,83
P04843	Dolichyl-diphosphooligosaccharide--protein glycosyltransferase subunit 1 OS=Homo sapiens OX=9606 GN=RPN1 PE=1 SV=1	121,26	2	2	2	2,94	3,62							64,06	1	1	1	1,47	1,65
P04844	Dolichyl-diphosphooligosaccharide--protein glycosyltransferase subunit 2 OS=Homo sapiens OX=9606 GN=RPN2 PE=1 SV=3	56,58	1	1	1	2,13	4,91							58,17	1	1	1	2,13	4,91
P04908	Histone H2A type 1-B/E OS=Homo sapiens OX=9606 GN=H2AC4 PE=1 SV=2	196,65	15	5	1	19,23	36,15	209,66	15	5	1	19,23	36,15	193,19	22	6	2	23,08	57,69
P05023	Sodium/potassium-transporting ATPase subunit alpha-1 OS=Homo sapiens OX=9606 GN=ATP1A1 PE=1 SV=1	120,09	4	4	4	3,96	5,38							61,06	1	1	1	0,99	1,08
P05109	Protein S100-A8 OS=Homo sapiens OX=9606 GN=S100A8 PE=1 SV=1	180,93	12	6	6	40	46,24	101,18	1	1	1	6,67	11,83						
P05141	ADP/ATP translocase 2 OS=Homo sapiens OX=9606 GN=SLC25A5 PE=1 SV=7	285,37	24	16	5	39,02	43,29	209,53	6	6	1	14,63	21,48	203,71	13	8	1	19,51	28,19
P05204	Non-histone chromosomal protein HMG-17 OS=Homo sapiens OX=9606 GN=HMGN2 PE=1 SV=3	82,27	2	1	1	4,35	16,67	73,78	2	1	1	4,35	16,67						
P05386	60S acidic ribosomal protein P1 OS=Homo sapiens OX=9606 GN=RPLP1 PE=1 SV=1	110,27	1	1	1	12,5	14,04	104,72	1	1	1	12,5	14,04	90,83	1	1	1	12,5	14,04
P05388	60S acidic ribosomal protein P0 OS=Homo sapiens OX=9606 GN=RPLP0 PE=1 SV=1	267,65	12	7	7	21,21	28,08	232,51	10	6	6	18,18	24,29	214,52	10	6	6	18,18	25,87
P05783	Keratin, type I cytoskeletal 18 OS=Homo sapiens OX=9606 GN=KRT18 PE=1 SV=2	261,02	16	13	11	23,21	34,42	195,47	6	4	3	7,14	14,19	179,22	8	6	5	10,71	19,77

P05787	Keratin, type II cytoskeletal 8 OS=Homo sapiens OX=9606 GN=KRT8 PE=1 SV=7	329,61	38	22	15	34,38	49,9	268,23	27	15	8	23,44	37,27	266,16	47	19	11	29,69	37,47
P06396	Gelsolin OS=Homo sapiens OX=9606 GN=GSN PE=1 SV=1							134,73	3	3	3	3,57	9,97	115,39	2	2	2	2,38	2,94
P06493	Cyclin-dependent kinase 1 OS=Homo sapiens OX=9606 GN=CDK1 PE=1 SV=3	236,64	10	8	8	21,62	33,67	178,33	4	4	4	10,81	20,88	155,18	6	5	5	13,51	23,57
P06576	ATP synthase subunit beta, mitochondrial OS=Homo sapiens OX=9606 GN=ATP5F1B PE=1 SV=3	139,88	3	3	3	6	8,88	108,2	2	2	2	4	6,62						
P06733	Alpha-enolase OS=Homo sapiens OX=9606 GN=ENO1 PE=1 SV=2	51,53	1	1	1	1,82	2,07	279,28	12	11	11	20	41,71	114,04	2	2	2	3,64	5,07
P06744	Glucose-6-phosphate isomerase OS=Homo sapiens OX=9606 GN=GPI PE=1	151,09	4	4	4	6,56	13,26							77,59	1	1	1	1,64	2,69
P06748	Nucleophosmin OS=Homo sapiens OX=9606 GN=NPM1 PE=1 SV=2	209,97	9	6	6	15	38,1	223,02	10	6	6	15	40,48	203,62	11	6	6	15	32,31
P06899	Histone H2B type 1-J OS=Homo sapiens OX=9606 GN=H2BC11 PE=1 SV=3	234,71	19	9	3	32,14	51,59	225,76	13	5	2	17,86	32,54	182,56	16	6	1	21,43	42,86
P07195	L-lactate dehydrogenase B chain OS=Homo sapiens OX=9606 GN=LDHB	65,3	1	1	1	2,86	3,29	141,52	4	3	2	8,57	9,58						
P07237	Protein disulfide-isomerase OS=Homo sapiens OX=9606 GN=P4HB PE=1 SV=3	43,6	1	1	1	1,61	7,48	165,33	4	4	4	6,45	13,78						
P07355	Annexin A2 OS=Homo sapiens OX=9606 GN=ANXA2 PE=1 SV=2	226,46	8	7	7	13,21	31,56	269,85	11	10	10	18,87	42,48	202,41	7	7	7	13,21	30,38
P07437	Tubulin beta chain OS=Homo sapiens OX=9606 GN=TUBB PE=1 SV=2	470,61	139	35	6	94,59	83,78	438,85	68	29	5	78,38	83,11	380,17	107	29	5	78,38	83,11
P07858	Cathepsin B OS=Homo sapiens OX=9606 GN=CTSB PE=1 SV=3	80,71	2	1	1	3,85	5,31	81,98	1	1	1	3,85	5,31	55,97	1	1	1	3,85	5,31
P07900	Heat shock protein HSP 90-alpha OS=Homo sapiens OX=9606							342	23	18	8	16,51	32,92	199,41	8	7	1	6,42	10,25
P07910	Heterogeneous nuclear ribonucleoproteins C1/C2 OS=Homo sapiens OX=9606 GN=HNRNPC PE=1 SV=4	164,85	8	5	5	10,42	21,9	195,77	9	6	6	12,5	29,74	150,62	9	5	5	10,42	20,26
P07951	Tropomyosin beta chain OS=Homo sapiens OX=9606 GN=TPM2 PE=1 SV=1							270,47	13	10	1	18,52	33,1	150,67	5	4	4	7,41	14,79
P08238	Heat shock protein HSP 90-beta OS=Homo sapiens OX=9606	264,96	13	11	11	10,38	16,85	401,41	34	26	15	24,53	45,17	255,45	16	14	8	13,21	22,79
P08670	Vimentin OS=Homo sapiens OX=9606 GN=VIM PE=1 SV=4	423,93	96	41	40	65,08	85,41	407,48	69	39	38	61,9	85,41	388,89	90	43	40	68,25	85,19
P08708	40S ribosomal protein S17 OS=Homo sapiens OX=9606 GN=RPS17 PE=1 SV=2	248,19	18	8	8	29,63	55,56	193,4	8	4	4	14,81	51,11	164,65	9	4	4	14,81	51,11

P08779	Keratin, type I cytoskeletal 16 OS=Homo sapiens OX=9606 GN=KRT16 PE=1 SV=4	308,73	27	16	5	32,65	32,56	286,99	21	14	5	28,57	35,52	294,73	38	19	6	38,78	39,53
P08865	40S ribosomal protein SA OS=Homo sapiens OX=9606 GN=RPSA PE=1 SV=4	293,51	15	9	9	33,33	49,49	279,19	11	9	9	33,33	54,24	233,51	12	8	8	29,63	39,66
P09493	Tropomyosin alpha-1 chain OS=Homo sapiens OX=9606 GN=TPM1 PE=1 SV=2							259,36	10	8	1	14,81	23,94	150,67	5	4	4	7,41	14,79
P09651	Heterogeneous nuclear ribonucleoprotein A1 OS=Homo sapiens OX=9606 GN=HNRNPA1 PE=1 SV=5	243,65	15	7	7	17,07	21,51	235,47	16	8	8	19,51	27,69	246,6	20	11	11	26,83	40,59
P09661	U2 small nuclear ribonucleoprotein A' OS=Homo sapiens OX=9606 GN=SNRPA1 PE=1 SV=2	86,64	2	2	2	4,88	24,71							45,82	1	1	1	2,44	13,73
P09874	Poly [ADP-ribose] polymerase 1 OS=Homo sapiens OX=9606 GN=PARP1 PE=1 SV=4	57,17	1	1	1	0,65	1,08	154,81	4	4	4	2,6	6,8	59,93	1	1	1	0,65	1,87
P0C0L4	Complement C4-A OS=Homo sapiens OX=9606 GN=C4A PE=1 SV=2	187,28	4	4	4	2,26	4,24	70,94	1	1	1	0,56	0,69	97,19	2	2	2	1,13	1,55
P0C0S5	Histone H2A.Z OS=Homo sapiens OX=9606 GN=H2AZ1 PE=1 SV=2	195,52	7	4	3	16,67	53,91	192,11	8	4	3	16,67	53,91	175,61	10	4	3	16,67	53,91
P0CF74	Immunoglobulin lambda constant 6 OS=Homo sapiens OX=9606 GN=IGLC6 PE=1 SV=1	71,32	3	1	1	10	7,55	58,78	2	1	1	10	7,55	61,58	2	1	1	10	7,55
P0DMV8	Heat shock 70 kDa protein 1A OS=Homo sapiens OX=9606 GN=HSPA1A PE=1 SV=1	255,7	13	11	6	13,58	18,88	207,6	8	7	5	8,64	15,29	175,91	7	6	4	7,41	10,45
P0DMV9	Heat shock 70 kDa protein 1B OS=Homo sapiens OX=9606 GN=HSPA1B PE=1 SV=1	255,7	13	11	6	13,58	18,88	207,6	8	7	5	8,64	15,29	175,91	7	6	4	7,41	10,45
P0DN76	Splicing factor U2AF 35 kDa subunit-like protein OS=Homo sapiens OX=9606 GN=U2AF1L5 PE=1 SV=1	104,21	1	1	1	2,22	5,42	53,62	1	1	1	2,22	5,83	74,13	1	1	1	2,22	5,42
P0DP09	Immunoglobulin kappa variable 1-13 OS=Homo sapiens OX=9606 GN=IGKV1-13 PE=3 SV=1	52,73	1	1	1	12,5	13,68	76,14	3	1	1	12,5	13,68	61,02	1	1	1	12,5	13,68
P10412	Histone H1.4 OS=Homo sapiens OX=9606 GN=H1-4 PE=1 SV=2	202,49	8	5	5	8,77	17,35	191,76	5	4	4	7,02	17,35	202,84	12	6	3	10,53	24,66
P10809	60 kDa heat shock protein, mitochondrial OS=Homo sapiens OX=9606 GN=HSPD1 PE=1 SV=2	164,61	7	5	5	6,85	9,42	221,93	7	6	6	8,22	11,34	105,07	2	2	2	2,74	4,19
P11021	Endoplasmic reticulum chaperone BiP OS=Homo sapiens OX=9606 GN=HSPA5 PE=1 SV=2	398,71	45	31	29	35,63	55,81	380,6	33	24	23	27,59	51,07	313,49	30	21	20	24,14	44,19

P11142	Heat shock cognate 71 kDa protein OS=Homo sapiens OX=9606 GN=HSPA8 PE=1 SV=1	370,08	57	25	22	30,49	45,2	323,67	24	19	17	23,17	35,6	324,65	31	21	19	25,61	42,72
P11279	Lysosome-associated membrane glycoprotein 1 OS=Homo sapiens OX=9606 GN=LAMP1 PE=1 SV=3	87,4	1	1	1	2,78	5,04							99,82	2	2	2	5,56	7,19
P11940	Polyadenylate-binding protein 1 OS=Homo sapiens OX=9606 GN=PABPC1 PE=1 SV=2	358,59	37	26	18	33,77	50,63	322,51	23	18	14	23,38	39,94	297,73	22	20	14	25,97	38,36
P12236	ADP/ATP translocase 3 OS=Homo sapiens OX=9606 GN=SLC25A6 PE=1 SV=4	273,51	21	13	2	31,71	42,95	193,12	6	6	1	14,63	26,17	202,79	12	8	1	19,51	32,89
P12268	Inosine-5'-monophosphate dehydrogenase 2 OS=Homo sapiens OX=9606 GN=IMPDH2 PE=1 SV=2	326,92	21	15	14	25,42	41,63	361,56	31	23	22	38,98	53,5	278,57	23	17	17	28,81	48,64
P12814	Alpha-actinin-1 OS=Homo sapiens OX=9606 GN=ACTN1 PE=1 SV=2	171,52	7	5	5	4,85	5,72	170,79	6	5	3	4,85	6,73	172,57	5	5	2	4,85	7,29
P12956	X-ray repair cross-complementing protein 6 OS=Homo sapiens OX=9606 GN=XRCC6 PE=1 SV=2	152,49	5	5	5	5,75	16,42	258,9	12	9	9	10,34	25,62	211,35	15	9	9	10,34	26,77
P13010	X-ray repair cross-complementing protein 5 OS=Homo sapiens OX=9606 GN=XRCC5 PE=1 SV=3	62,37	1	1	1	1,11	3,01	222,9	8	8	8	8,89	16,26	261,59	24	14	14	15,56	28,55
P13639	Elongation factor 2 OS=Homo sapiens OX=9606 GN=EEF2 PE=1 SV=4	258,94	17	13	13	12,38	22,26	234,34	12	9	9	8,57	17,72	164,46	8	6	6	5,71	10,26
P13645	Keratin, type I cytoskeletal 10 OS=Homo sapiens OX=9606 GN=KRT10 PE=1 SV=6	459,84	137	44	36	91,67	65,58	426,77	80	37	31	77,08	62,67	385	190	43	36	89,58	62,67
P13647	Keratin, type II cytoskeletal 5 OS=Homo sapiens OX=9606 GN=KRT5 PE=1 SV=3	353,02	41	23	11	34,33	34,41	322,03	30	20	11	29,85	30,51	295,99	48	26	13	38,81	35,76
P13804	Electron transfer flavoprotein subunit alpha, mitochondrial OS=Homo sapiens OX=9606 GN=ETFA PE=1 SV=1							135,23	2	1	1	2,78	5,71	94,54	1	1	1	2,78	5,71
P14618	Pyruvate kinase PKM OS=Homo sapiens OX=9606 GN=PKM PE=1 SV=4	86,69	2	2	2	3,23	5,46							49,12	1	1	1	1,61	2,07
P14866	Heterogeneous nuclear ribonucleoprotein L OS=Homo sapiens OX=9606 GN=HNRNPL PE=1 SV=2	243,05	13	10	10	16,13	27,16	173,92	7	5	5	8,06	16,81	182,78	11	6	6	9,68	21,39

P14923	Junction plakoglobin OS=Homo sapiens OX=9606 GN=JUP PE=1 SV=3	88,94	2	2	2	3,08	4,03	133,34	3	3	3	4,62	5,64	156,71	4	3	3	4,62	5,64
P15814	Immunoglobulin lambda-like polypeptide 1 OS=Homo sapiens OX=9606 GN=IGLL1 PE=1 SV=1	71,32	3	1	1	4,76	3,76	58,78	2	1	1	4,76	3,76	61,58	2	1	1	4,76	3,76
P15880	40S ribosomal protein S2 OS=Homo sapiens OX=9606 GN=RPS2 PE=1 SV=2	212,8	7	6	6	12,5	24,57	200,51	5	5	5	10,42	23,89	190,26	7	6	6	12,5	25,94
P16383	Intron Large complex component GCFC2 OS=Homo sapiens OX=9606 GN=GCFC2 PE=1 SV=2	291,74	17	14	14	12,61	26,38	162,51	3	3	3	2,7	5,63	208	9	8	8	7,21	15,49
P16402	Histone H1.3 OS=Homo sapiens OX=9606 GN=H1-3 PE=1 SV=2	202,49	8	5	5	8,77	17,19	191,76	5	4	4	7,02	17,19						
P16403	Histone H1.2 OS=Homo sapiens OX=9606 GN=H1-2 PE=1 SV=2	202,49	8	5	5	9,43	17,84	191,76	5	4	4	7,55	17,84						
P16989	Y-box-binding protein 3 OS=Homo sapiens OX=9606 GN=YBX3 PE=1 SV=4	216,85	10	8	5	16,67	31,72							181,88	6	5	2	10,42	18,01
P17844	Probable ATP-dependent RNA helicase DDX5 OS=Homo sapiens OX=9606 GN=DDX5 PE=1 SV=1	204,86	14	11	6	13,1	16,12	167,03	4	4	3	4,76	5,7	219,35	11	10	8	11,9	17,26
P18077	60S ribosomal protein L35a OS=Homo sapiens OX=9606 GN=RPL35A PE=1 SV=2	111,62	5	4	4	16,67	21,82							71,09	1	1	1	4,17	7,27
P18124	60S ribosomal protein L7 OS=Homo sapiens OX=9606 GN=RPL7 PE=1 SV=1	220,21	8	6	6	9,68	26,61	156,27	4	3	3	4,84	16,94	181,44	6	5	5	8,06	24,6
P18621	60S ribosomal protein L17 OS=Homo sapiens OX=9606 GN=RPL17 PE=1 SV=3	206,81	10	7	7	17,5	39,13	160,42	6	5	5	12,5	31,52	105,71	2	2	2	5	13,59
P19105	Myosin regulatory light chain 12A OS=Homo sapiens OX=9606 GN=MYL12A PE=1 SV=2	69,12	1	1	1	4,35	5,85							64,81	1	1	1	4,35	5,85
P19338	Nucleolin OS=Homo sapiens OX=9606 GN=NCL PE=1 SV=3	127,51	2	2	2	1,75	5,49	200,86	7	5	5	4,39	9,44	81,47	2	2	2	1,75	3,66
P19474	E3 ubiquitin-protein ligase TRIM21 OS=Homo sapiens OX=9606 GN=TRIM21 PE=1 SV=1	61,38	1	1	1	1,79	3,79	139,16	2	2	2	3,57	5,68	96,5	2	2	2	3,57	5,68
P19784	Casein kinase II subunit alpha' OS=Homo sapiens OX=9606 GN=CSNK2A2 PE=1 SV=1	167,63	4	4	4	8,33	14	64,02	1	1	1	2,08	2,57						

P20700	Lamin-B1 OS=Homo sapiens OX=9606 GN=LMNB1 PE=1 SV=2	110,62	4	3	3	3,41	8,36	169,17	5	4	4	4,55	14,16	194,91	10	8	7	9,09	22,01
P20930	Filaggrin OS=Homo sapiens OX=9606 GN=FLG PE=1 SV=3							82,01	1	1	1	0,2	0,34	131,03	3	3	3	0,6	0,57
P21333	Filamin-A OS=Homo sapiens OX=9606 GN=FLNA PE=1 SV=4	103,67	3	3	1	1,24	1,17	68,1	1	1	1	0,41	0,72						
P22087	rRNA 2'-O-methyltransferase fibrillarin OS=Homo sapiens OX=9606 GN=FBL PE=1 SV=2	79,67	1	1	1	2,22	4,05	75,86	1	1	1	2,22	4,05	122,4	3	3	3	6,67	14,02
P22234	Multifunctional protein ADE2 OS=Homo sapiens OX=9606 GN=PAICS PE=1 SV=3	59,51	1	1	1	1,92	3,06	59,73	1	1	1	1,92	3,06	64,65	1	1	1	1,92	3,06
P22626	Heterogeneous nuclear ribonucleoproteins A2/B1 OS=Homo sapiens OX=9606 GN=HNRNPA2B1 PE=1 SV=2	193,07	9	7	7	16,28	26,91	220,77	11	8	8	18,6	35,69	184,3	7	6	6	13,95	22,1
P22695	Cytochrome b-c1 complex subunit 2, mitochondrial OS=Homo sapiens OX=9606 GN=UQCRC2 PE=1 SV=3	170,99	4	4	4	9,52	10,6							109,63	1	1	1	2,38	4,64
P23396	40S ribosomal protein S3 OS=Homo sapiens OX=9606 GN=RPS3 PE=1 SV=2	354,49	29	19	19	51,35	75,31	276,38	12	9	9	24,32	47,74	264,88	16	13	13	35,14	57,61
P23527	Histone H2B type 1-O OS=Homo sapiens OX=9606 GN=H2BC17 PE=1 SV=3	234,71	19	9	3	32,14	51,59	225,76	13	5	2	17,86	32,54	182,56	16	6	1	21,43	42,86
P25398	40S ribosomal protein S12 OS=Homo sapiens OX=9606 GN=RPS12 PE=1 SV=3	238,92	9	8	8	40	60,61	110,13	2	2	2	10	18,18	150,72	5	5	5	25	40,15
P25705	ATP synthase subunit alpha, mitochondrial OS=Homo sapiens OX=9606 GN=ATP5F1A PE=1 SV=1	318,6	21	19	19	28,36	35,99	219,06	7	5	5	7,46	14,1	218,51	9	7	7	10,45	18,08
P26373	60S ribosomal protein L13 OS=Homo sapiens OX=9606 GN=RPL13 PE=1 SV=4	208,18	8	6	6	11,54	31,28	134,76	3	3	3	5,77	20,85	156,7	6	4	4	7,69	21,33
P27449	V-type proton ATPase 16 kDa proteolipid subunit OS=Homo sapiens OX=9606 GN=ATP6V0C PE=1 SV=1							130,82	2	2	2	28,57	31,61	100,51	3	1	1	14,29	20
P27635	60S ribosomal protein L10 OS=Homo sapiens OX=9606 GN=RPL10 PE=1 SV=4	232,76	15	10	10	23,26	53,74	174,84	6	5	5	11,63	33,64	175,16	10	7	7	16,28	42,06

P29692	Elongation factor 1-delta OS=Homo sapiens OX=9606 GN=EEF1D PE=1 SV=5	94,29	1	1	1	2,94	8,54	184,49	5	4	4	11,76	20,64						
P30050	60S ribosomal protein L12 OS=Homo sapiens OX=9606 GN=RPL12 PE=1 SV=1	187,79	4	3	3	11,54	23,64	134,15	3	2	2	7,69	14,55	173,97	5	4	4	15,38	35,15
P30536	Translocator protein OS=Homo sapiens OX=9606 GN=TSPO PE=1 SV=3	39,47	1	1	1	9,09	4,73							49,27	1	1	1	9,09	4,73
P31146	Coronin-1A OS=Homo sapiens OX=9606 GN=CORO1A PE=1 SV=4	172,91	5	4	4	7,27	16,49							98,56	2	2	2	3,64	7,59
P31350	Ribonucleoside-diphosphate reductase subunit M2 OS=Homo sapiens OX=9606 GN=RRM2 PE=1 SV=1	291,4	16	12	12	26,67	42,67	179,69	4	3	3	6,67	12,6	214,2	9	7	7	15,56	25,19
P31689	DnaJ homolog subfamily A member 1 OS=Homo sapiens OX=9606 GN=DNAJA1 PE=1 SV=2	211,65	12	6	6	10,17	24,18	168,82	7	4	4	6,78	15,11	185,38	9	6	6	10,17	24,18
P31942	Heterogeneous nuclear ribonucleoprotein H3 OS=Homo sapiens OX=9606 GN=HNRNPH3 PE=1 SV=2	41,19	1	1	1	2,86	2,6							66,69	1	1	1	2,86	3,47
P31943	Heterogeneous nuclear ribonucleoprotein H OS=Homo sapiens OX=9606 GN=HNRNPH1 PE=1 SV=4	219,9	6	5	3	11,11	16,26	196,84	4	3	1	6,67	9,8	206,73	7	6	4	13,33	20,49
P31944	Caspase-14 OS=Homo sapiens OX=9606 GN=CASP14 PE=1 SV=2	60,23	1	1	1	2,94	4,96							53,94	1	1	1	2,94	4,55
P31946	14-3-3 protein beta/alpha OS=Homo sapiens OX=9606 GN=YWHAB PE=1 SV=3	81,75	1	1	1	3,23	5,69	128,07	1	1	1	3,23	5,69	91,83	1	1	1	3,23	5,69
P32969	60S ribosomal protein L9 OS=Homo sapiens OX=9606 GN=RPL9 PE=1 SV=1	241,6	12	8	8	24,24	54,17	207,06	6	5	5	15,15	45,31	166,52	5	3	3	9,09	22,92
P33778	Histone H2B type 1-B OS=Homo sapiens OX=9606 GN=H2BC3 PE=1 SV=2	234,71	19	9	3	32,14	51,59	225,76	13	5	2	17,86	32,54	182,56	16	6	1	21,43	42,86
P33993	DNA replication licensing factor MCM7 OS=Homo sapiens OX=9606 GN=MCM7 PE=1 SV=4	81,66	1	1	1	1,1	1,81							73,54	1	1	1	1,1	1,81
P35221	Catenin alpha-1 OS=Homo sapiens OX=9606 GN=CTNNA1 PE=1 SV=1							76,48	1	1	1	0,83	2,76	45,55	1	1	1	0,83	3,2

P35222	Catenin beta-1 OS=Homo sapiens OX=9606 GN=CTNNB1 PE=1 SV=1	61,67	1	1	1	1,56	2,69	91,91	1	1	1	1,56	1,54	110,91	2	2	2	3,12	4,23
P35232	Prohibitin OS=Homo sapiens OX=9606 GN=PHB PE=1 SV=1							325,54	29	19	19	65,52	82,72	248,7	17	12	12	41,38	67,28
P35268	60S ribosomal protein L22 OS=Homo sapiens OX=9606 GN=RPL22 PE=1 SV=2	173,7	8	4	4	15,38	49,22	160,25	5	4	4	15,38	49,22	132,4	4	3	3	11,54	39,84
P35527	Keratin, type I cytoskeletal 9 OS=Homo sapiens OX=9606 GN=KRT9 PE=1 SV=3	463,92	77	26	26	50,98	66,93	480,93	91	31	31	60,78	70,95	414,43	112	33	32	64,71	71,91
P35579	Myosin-9 OS=Homo sapiens OX=9606 GN=MYH9 PE=1 SV=4	276,11	16	14	14	4,28	9,8	512,41	127	83	76	25,38	49,54	456,46	144	88	77	26,91	51,99
P35580	Myosin-10 OS=Homo sapiens OX=9606 GN=MYH10 PE=1 SV=3							281,9	18	12	8	3,66	9,77	320,25	31	24	15	7,32	15,38
P35637	RNA-binding protein FUS OS=Homo sapiens OX=9606 GN=FUS PE=1 SV=1	118,67	2	2	2	4	3,04							84,03	1	1	1	2	2,66
P35908	Keratin, type II cytoskeletal 2 epidermal OS=Homo sapiens OX=9606 GN=KRT2 PE=1 SV=2	473,46	98	48	31	72,73	79,19	432,55	65	40	28	60,61	75,59	422,07	128	51	38	77,27	81,06
P36578	60S ribosomal protein L4 OS=Homo sapiens OX=9606 GN=RPL4 PE=1 SV=5	260,71	14	11	11	12,09	29,74	86,7	1	1	1	1,1	2,81	140,32	4	4	4	4,4	12,88
P36873	Serine/threonine-protein phosphatase PP1-gamma catalytic subunit OS=Homo sapiens OX=9606 GN=PPP1CC PE=1 SV=1	96,3	3	2	1	5,56	9,29							169,45	5	5	1	13,89	17,65
P36957	Dihydrolipoyllysine-residue succinyltransferase component of 2-oxoglutarate dehydrogenase complex, mitochondrial OS=Homo sapiens OX=9606 GN=DLST PE=1 SV=4	88,68	2	1	1	1,89	2,87	102,13	1	1	1	1,89	2,87	92,65	2	1	1	1,89	2,87
P38159	RNA-binding motif protein, X chromosome OS=Homo sapiens OX=9606 GN=RBMX PE=1 SV=3	128,77	3	2	2	2,9	7,67	174,15	5	5	5	7,25	18,16	199,24	8	7	7	10,14	21,23
P38646	Stress-70 protein, mitochondrial OS=Homo sapiens OX=9606 GN=HSPA9 PE=1 SV=2	299,46	23	20	20	22,47	40,35	279,43	12	11	11	12,36	27,98	217,5	10	9	9	10,11	19,88
P39019	40S ribosomal protein S19 OS=Homo sapiens OX=9606 GN=RPS19 PE=1 SV=2	258,11	13	9	9	32,14	50,34	223,06	8	5	5	17,86	34,48	74,24	1	1	1	1,82	3,41
P39023	60S ribosomal protein L3 OS=Homo sapiens OX=9606 GN=RPL3 PE=1 SV=2	188,57	8	7	7	8,24	17,87	108,69	1	1	1	1,18	6,45	131,74	3	3	3	3,53	10,67

P39656	Dolichyl-diphosphooligosaccharide--protein glycosyltransferase 48 kDa subunit OS=Homo sapiens OX=9606 GN=DDOST PE=1 SV=4	82,36	1	1	1	2,33	1,97							58,34	1	1	1	2,33	1,97
P40429	60S ribosomal protein L13a OS=Homo sapiens OX=9606 GN=RPL13A PE=1 SV=2	152,98	5	5	5	10	23,15							105,8	2	2	2	4	9,36
P42285	Exosome RNA helicase MTR4 OS=Homo sapiens OX=9606 GN=MTREX PE=1 SV=3	167,46	4	4	4	3,03	5,95							55,3	1	1	1	0,76	2,11
P42677	40S ribosomal protein S27 OS=Homo sapiens OX=9606 GN=RPS27 PE=1 SV=3	160,65	8	4	4	25	41,67	105,52	2	2	2	12,5	25	144,82	5	3	3	18,75	40,48
P43243	Matrin-3 OS=Homo sapiens OX=9606 GN=MATR3 PE=1 SV=2	345,02	36	22	22	19,13	38,02	301,56	20	17	17	14,78	34,83	283,17	23	16	16	13,91	30,81
P43246	DNA mismatch repair protein Msh2 OS=Homo sapiens OX=9606 GN=MSH2 PE=1 SV=1	105,2	3	3	3	2,86	2,46							76,41	2	2	2	1,9	4,28
P43686	26S proteasome regulatory subunit 6B OS=Homo sapiens OX=9606 GN=PSMC4 PE=1 SV=2	59,53	1	1	1	2,08	4,31	102,51	2	2	2	4,17	7,66	45,42	1	1	1	2,08	2,87
P46776	60S ribosomal protein L27a OS=Homo sapiens OX=9606 GN=RPL27A PE=1 SV=2	121,79	2	2	2	6,06	23,65							105,68	2	2	2	6,06	16,89
P46777	60S ribosomal protein L5 OS=Homo sapiens OX=9606 GN=RPL5 PE=1 SV=3	286,5	20	13	13	22,41	50,51	206,68	9	7	7	12,07	28,28	201,79	10	8	8	13,79	37,37
P46778	60S ribosomal protein L21 OS=Homo sapiens OX=9606 GN=RPL21 PE=1 SV=2	174,73	6	5	5	13,16	30,62	86,37	1	1	1	2,63	9,38	152,56	3	2	2	5,26	18,75
P46779	60S ribosomal protein L28 OS=Homo sapiens OX=9606 GN=RPL28 PE=1 SV=3	150,31	6	5	5	16,13	35,04	118,44	2	2	2	6,45	14,6	142,69	4	3	3	9,68	17,52
P46781	40S ribosomal protein S9 OS=Homo sapiens OX=9606 GN=RPS9 PE=1 SV=3	167,69	10	6	6	13,33	29,38	101,72	1	1	1	2,22	4,64	156,15	7	7	7	15,56	30,93
P46782	40S ribosomal protein S5 OS=Homo sapiens OX=9606 GN=RPS5 PE=1 SV=4	277,2	14	9	9	27,27	38,24	238,34	8	6	6	18,18	26,96	184,63	4	3	3	9,09	18,14
P46783	40S ribosomal protein S10 OS=Homo sapiens OX=9606 GN=RPS10 PE=1 SV=1	222,61	8	5	5	17,86	38,18	88,4	2	1	1	3,57	5,45	168,4	5	4	4	14,29	20

P46821	Microtubule-associated protein 1B OS=Homo sapiens OX=9606 GN=MAP1B PE=1 SV=2	148,93	5	5	5	1,83	2,63							54,65	1	1	1	0,37	0,61
P47756	F-actin-capping protein subunit beta OS=Homo sapiens OX=9606 GN=CAPZB PE=1 SV=4	115,79	3	2	2	5,56	12,64	164,18	7	5	5	13,89	28,52	143,47	6	3	3	8,33	17,69
P48047	ATP synthase subunit O, mitochondrial OS=Homo sapiens OX=9606 GN=ATP5PO PE=1 SV=1	117,52	3	2	2	6,67	10,8							46,17	1	1	1	3,33	5,16
P48643	T-complex protein 1 subunit epsilon OS=Homo sapiens OX=9606 GN=CCT5 PE=1 SV=1	115,05	1	1	1	1,52	5,55	106,12	1	1	1	1,52	5,55	86,79	1	1	1	1,52	5,55
P48729	Casein kinase I isoform alpha OS=Homo sapiens OX=9606 GN=CSNK1A1 PE=1 SV=2	107,43	2	2	2	3,92	8,9							87,98	1	1	1	1,96	6,23
P49454	Centromere protein F OS=Homo sapiens OX=9606 GN=CENPF PE=1 SV=3	173,94	5	5	5	1,13	1,8							80,03	2	2	2	0,45	1,03
P50402	Emerin OS=Homo sapiens OX=9606 GN=EMD PE=1 SV=1	124,05	3	2	2	8	11,42							99,58	3	2	2	8	11,42
P50454	Serpin H1 OS=Homo sapiens OX=9606 GN=SERPINH1 PE=1 SV=2							53,2	1	1	1	1,89	6,22	59,73	1	1	1	1,89	6,22
P50552	Vasodilator-stimulated phosphoprotein OS=Homo sapiens OX=9606 GN=VASP PE=1 SV=3	380,83	51	20	20	46,51	52,11	342,08	27	16	16	37,21	47,63	288,57	26	17	17	39,53	46,58
P50914	60S ribosomal protein L14 OS=Homo sapiens OX=9606 GN=RPL14 PE=1 SV=4	160,93	3	3	3	5,88	16,28	118,1	2	2	2	3,92	11,16	109,19	2	2	2	3,92	11,16
P51114	Fragile X mental retardation syndrome-related protein 1 OS=Homo sapiens OX=9606 GN=FXR1 PE=1 SV=3	217,56	8	7	4	7,69	12,08	200,67	5	5	5	5,49	9,82	183,43	8	5	3	5,49	9,82
P51116	Fragile X mental retardation syndrome-related protein 2 OS=Homo sapiens OX=9606 GN=FXR2 PE=1 SV=2	181,35	8	7	4	7,87	12,33							143,12	4	4	2	4,49	6,54
P51571	Translocon-associated protein subunit delta OS=Homo sapiens OX=9606 GN=SSR4 PE=1 SV=1	73,26	1	1	1	7,14	6,36							57,63	1	1	1	7,14	10,98

P51991	Heterogeneous nuclear ribonucleoprotein A3 OS=Homo sapiens OX=9606 GN=HNRNPA3 PE=1 SV=2	53,88	1	1	1	2,13	3,7							62,67	1	1	1	2,13	3,44
P52272	Heterogeneous nuclear ribonucleoprotein M OS=Homo sapiens OX=9606 GN=HNRNPM PE=1 SV=3	246,67	15	13	13	14,29	21,92	285,93	16	15	15	16,48	26,3	259,31	20	17	17	18,68	27,4
P52292	Importin subunit alpha-1 OS=Homo sapiens OX=9606 GN=KPNA2 PE=1 SV=1	135,66	3	3	3	6	12,85	141,11	3	3	3	6	13,8	57,28	1	1	1	2	6,24
P52597	Heterogeneous nuclear ribonucleoprotein F OS=Homo sapiens OX=9606 GN=HNRNPF PE=1 SV=3	238,23	10	7	5	18,42	24,1	165,82	4	3	1	7,89	8,43	183,44	6	5	3	13,16	20,48
P52701	DNA mismatch repair protein Msh6 OS=Homo sapiens OX=9606 GN=MSH6 PE=1 SV=2	155,6	3	3	3	1,66	3,75	63,55	1	1	1	0,55	2,13						
P55072	Transitional endoplasmic reticulum ATPase OS=Homo sapiens OX=9606 GN=VCP PE=1 SV=4	133,17	4	4	4	4,04	4,09	359,12	34	25	25	25,25	42,18	300,32	24	21	21	21,21	35,73
P55209	Nucleosome assembly protein 1-like 1 OS=Homo sapiens OX=9606 GN=NAP1L1 PE=1 SV=1	113,38	2	2	2	4	7,42	171,62	5	5	4	10	21,74	82,65	2	2	2	4	7,42
P55884	Eukaryotic translation initiation factor 3 subunit B OS=Homo sapiens OX=9606 GN=EIF3B PE=1 SV=3	173,93	4	4	4	4,26	11,3	90,11	1	1	1	1,06	1,72	94,37	1	1	1	1,06	1,72
P56134	ATP synthase subunit f, mitochondrial OS=Homo sapiens OX=9606 GN=ATP5MF PE=1 SV=3	52,91	1	1	1	6,25	11,7	60,26	1	1	1	6,25	11,7						
P58876	Histone H2B type 1-D OS=Homo sapiens OX=9606 GN=H2BC5 PE=1 SV=2	240,51	20	9	3	32,14	51,59	239,22	15	6	3	21,43	32,54	193,26	19	7	2	25	42,86
P60174	Triosephosphate isomerase OS=Homo sapiens OX=9606 GN=TPI1 PE=1 SV=4	97,02	1	1	1	3,57	5,22	125,7	2	2	2	7,14	11,24	78,63	1	1	1	3,57	5,22
P60228	Eukaryotic translation initiation factor 3 subunit E OS=Homo sapiens OX=9606 GN=EIF3E PE=1 SV=1	158,34	4	4	4	7,55	10,56	97,24	1	1	1	1,89	4,49						

P60660	Myosin light polypeptide 6 OS=Homo sapiens OX=9606 GN=MYL6 PE=1 SV=2	154,26	4	3	3	20	26,49	189,42	6	4	4	26,67	36,42	149,79	6	3	3	20	26,49
P60709	Actin, cytoplasmic 1 OS=Homo sapiens OX=9606 GN=ACTB PE=1 SV=1	457,24	186	35	12	94,59	81,87	436,4	108	26	13	70,27	76,53	380,39	212	32	10	86,49	84,27
P60866	40S ribosomal protein S20 OS=Homo sapiens OX=9606 GN=RPS20 PE=1 SV=1	230,17	11	7	7	29,17	52,94	165,16	5	3	3	12,5	22,69	157,76	6	3	3	12,5	22,69
P61158	Actin-related protein 3 OS=Homo sapiens OX=9606 GN=ACTR3 PE=1 SV=3	57,33	1	1	1	2,22	2,63	145,93	3	3	3	6,67	11,24	151,61	6	4	4	8,89	22,01
P61160	Actin-related protein 2 OS=Homo sapiens OX=9606 GN=ACTR2 PE=1 SV=1							65,46	1	1	1	2,04	3,05	93,78	3	2	2	4,08	12,18
P61247	40S ribosomal protein S3a OS=Homo sapiens OX=9606 GN=RPS3A PE=1 SV=2	350,85	39	22	22	42,31	64,77	257,66	16	12	12	23,08	39,02	247,05	19	14	14	26,92	50,38
P61254	60S ribosomal protein L26 OS=Homo sapiens OX=9606 GN=RPL26 PE=1 SV=1	216,01	10	6	6	14,63	37,24	146,18	4	3	3	7,32	24,14	180,27	8	6	1	14,63	30,34
P61313	60S ribosomal protein L15 OS=Homo sapiens OX=9606 GN=RPL15 PE=1 SV=2	127,23	3	2	2	4,44	12,25	85,21	2	1	1	2,22	7,84	78,93	2	1	1	2,22	7,84
P61353	60S ribosomal protein L27 OS=Homo sapiens OX=9606 GN=RPL27 PE=1 SV=2	181,77	10	5	5	14,29	43,38	142,97	4	3	3	8,57	32,35	139,46	4	4	4	11,43	38,24
P61513	60S ribosomal protein L37a OS=Homo sapiens OX=9606 GN=RPL37A PE=1 SV=2	104,05	2	2	2	8,7	28,26							89,2	2	2	2	8,7	28,26
P61978	Heterogeneous nuclear ribonucleoprotein K OS=Homo sapiens OX=9606 GN=HNRNPK PE=1 SV=1	85,54	2	2	2	3,7	5,62	155,09	3	3	3	5,56	7,99	76,34	1	1	1	1,85	2,59
P62081	40S ribosomal protein S7 OS=Homo sapiens OX=9606 GN=RPS7 PE=1 SV=1	269,86	20	14	14	40	51,55	235,79	8	6	6	17,14	30,41	216,55	12	8	8	22,86	41,75
P62136	Serine/threonine-protein phosphatase PP1-alpha catalytic subunit OS=Homo sapiens OX=9606 GN=PPP1CA PE=1 SV=1	145,61	4	4	3	11,11	16,97	115,19	3	3	3	8,33	11,82	178,8	6	6	2	16,67	22,12

P62195	26S proteasome regulatory subunit 8 OS=Homo sapiens OX=9606 GN=PSMC5 PE=1 SV=1	111,02	2	2	1	3,39	6,16	92,72	1	1	1	1,69	3,2	94,36	2	2	2	3,39	5,42
P62241	40S ribosomal protein S8 OS=Homo sapiens OX=9606 GN=RPS8 PE=1 SV=2	273,35	15	9	9	17,65	40,87	163,69	3	3	3	5,88	17,31	252,03	15	11	11	21,57	52,4
P62244	40S ribosomal protein S15a OS=Homo sapiens OX=9606 GN=RPS15A PE=1 SV=2	262,78	11	9	9	40,91	65,38	168,42	3	3	3	13,64	21,54	151,78	4	4	4	18,18	32,31
P62249	40S ribosomal protein S16 OS=Homo sapiens OX=9606 GN=RPS16 PE=1 SV=2	221,93	14	9	9	31,03	45,89	201,53	6	5	5	17,24	33,56	194,41	11	8	8	27,59	43,84
P62263	40S ribosomal protein S14 OS=Homo sapiens OX=9606 GN=RPS14 PE=1 SV=3	263,22	16	7	7	25	35,1	255,45	12	8	8	28,57	35,1	213,2	11	7	7	25	35,1
P62266	40S ribosomal protein S23 OS=Homo sapiens OX=9606 GN=RPS23 PE=1 SV=3	150,69	4	3	3	9,38	27,97							120,26	2	2	2	6,25	27,27
P62269	40S ribosomal protein S18 OS=Homo sapiens OX=9606 GN=RPS18 PE=1 SV=3	241,69	24	14	14	38,89	49,34	212,38	12	9	9	25	42,76	196,99	14	11	11	30,56	47,37
P62273	40S ribosomal protein S29 OS=Homo sapiens OX=9606 GN=RPS29 PE=1 SV=2	88,22	3	2	2	16,67	33,93	88,34	1	1	1	8,33	19,64						
P62277	40S ribosomal protein S13 OS=Homo sapiens OX=9606 GN=RPS13 PE=1 SV=2	223,91	12	7	7	21,21	40,4	179,95	4	3	3	9,09	28,48	168,34	9	5	5	15,15	39,07
P62280	40S ribosomal protein S11 OS=Homo sapiens OX=9606 GN=RPS11 PE=1 SV=3	279,43	17	11	11	31,43	58,86	177,3	7	6	6	17,14	29,75	219,82	10	8	8	22,86	41,77
P62314	Small nuclear ribonucleoprotein Sm D1 OS=Homo sapiens OX=9606 GN=SNRPD1 PE=1 SV=1	124,88	5	2	2	7,69	27,73	73,59	3	1	1	3,85	16,81	110,97	5	2	2	7,69	27,73
P62316	Small nuclear ribonucleoprotein Sm D2 OS=Homo sapiens OX=9606 GN=SNRPD2 PE=1 SV=1	136,78	2	2	2	9,09	16,95	109,23	2	2	2	9,09	16,95	106,43	2	2	2	9,09	16,95
P62318	Small nuclear ribonucleoprotein Sm D3 OS=Homo sapiens OX=9606 GN=SNRPD3 PE=1 SV=1	118,29	2	2	2	9,09	24,6	144,68	3	2	2	9,09	24,6	135,04	3	2	2	9,09	24,6

P62424	60S ribosomal protein L7a OS=Homo sapiens OX=9606 GN=RPL7A PE=1 SV=2	259,92	14	12	12	20	41,35	118,11	3	3	3	5	13,91	226,23	11	9	9	15	34,96
P62701	40S ribosomal protein S4, X isoform OS=Homo sapiens OX=9606 GN=RPS4X PE=1 SV=2	311,72	34	18	18	37,5	55,13	263,25	17	11	11	22,92	47,91	259,88	18	14	14	29,17	47,53
P62714	Serine/threonine-protein phosphatase 2A catalytic subunit beta isoform OS=Homo sapiens OX=9606 GN=PPP2CB PE=1 SV=1	146,2	3	3	3	9,38	9,71							59,57	1	1	1	3,12	2,27
P62750	60S ribosomal protein L23a OS=Homo sapiens OX=9606 GN=RPL23A PE=1 SV=1	226,94	12	8	8	20	42,31	114,27	2	2	2	5	13,46	134,7	5	5	5	12,5	27,56
P62753	40S ribosomal protein S6 OS=Homo sapiens OX=9606 GN=RPS6 PE=1 SV=1	245,88	12	10	10	15,38	41,37	85,04	1	1	1	1,54	5,62	207,63	8	8	8	12,31	30,12
P62805	Histone H4 OS=Homo sapiens OX=9606 GN=H4C1 PE=1 SV=2	286,21	28	13	13	52	59,22	267,75	32	12	12	48	59,22	255,12	46	13	13	52	59,22
P62807	Histone H2B type 1-C/E/F/G/I OS=Homo sapiens OX=9606 GN=H2BC4 PE=1 SV=4	240,51	20	9	3	32,14	51,59	239,22	15	6	3	21,43	32,54	193,26	19	7	2	25	42,86
P62826	GTP-binding nuclear protein Ran OS=Homo sapiens OX=9606 GN=RAN PE=1 SV=3							133,44	4	4	4	14,81	39,35	51,28	1	1	1	3,7	5,09
P62829	60S ribosomal protein L23 OS=Homo sapiens OX=9606 GN=RPL23 PE=1 SV=1	257,64	14	9	9	34,62	53,57	263,64	9	9	9	34,62	52,86	207,67	11	7	7	26,92	50,71
P62841	40S ribosomal protein S15 OS=Homo sapiens OX=9606 GN=RPS15 PE=1 SV=2	199,86	30	5	5	18,52	51,72	156	13	3	3	11,11	24,14	167,15	21	4	4	14,81	42,76
P62847	40S ribosomal protein S24 OS=Homo sapiens OX=9606 GN=RPS24 PE=1 SV=1	132,02	4	2	2	5,56	18,05	124,26	4	2	2	5,56	18,05	76,22	2	1	1	2,78	9,02
P62851	40S ribosomal protein S25 OS=Homo sapiens OX=9606 GN=RPS25 PE=1 SV=1	178,05	7	5	5	14,71	32,8	149,18	6	4	4	11,76	22,4	140,35	6	5	5	14,71	32,8
P62854	40S ribosomal protein S26 OS=Homo sapiens OX=9606 GN=RPS26 PE=1 SV=3	142,18	6	3	3	11,11	31,3	87,53	3	1	1	3,7	13,04	127,18	5	3	3	11,11	33,91

P62888	60S ribosomal protein L30 OS=Homo sapiens OX=9606 GN=RPL30 PE=1 SV=2	206,4	8	5	5	26,32	59,13	83,2	1	1	1	5,26	13,91	166,64	6	5	5	26,32	58,26
P62899	60S ribosomal protein L31 OS=Homo sapiens OX=9606 GN=RPL31 PE=1 SV=1	171,52	8	6	6	19,35	29,6	77,42	1	1	1	3,23	7,2	153,9	5	5	5	16,13	35,2
P62906	60S ribosomal protein L10a OS=Homo sapiens OX=9606 GN=RPL10A PE=1 SV=2	232,89	12	8	8	19,51	38,25	161,77	5	4	4	9,76	16,13	186,26	7	5	5	12,2	31,34
P62913	60S ribosomal protein L11 OS=Homo sapiens OX=9606 GN=RPL11 PE=1 SV=2	215,9	15	8	8	25	36,52	180,2	5	4	4	12,5	23,03	156,55	7	6	6	18,75	29,78
P62917	60S ribosomal protein L8 OS=Homo sapiens OX=9606 GN=RPL8 PE=1 SV=2	226,58	8	6	6	11,54	17,12	142,43	3	3	3	5,77	14,4	163,05	5	5	5	9,62	14,4
P62937	Peptidyl-prolyl cis-trans isomerase A OS=Homo sapiens OX=9606 GN=PPIA PE=1 SV=2	104,45	2	2	2	9,52	14,55	206,89	9	6	6	28,57	43,64	60,47	1	1	1	4,76	5,45
P62979	Ubiquitin-40S ribosomal protein S27a OS=Homo sapiens OX=9606 GN=RPS27A PE=1 SV=2	150,41	3	3	3	8,82	28,21	162,36	4	3	3	8,82	21,79	145,67	5	3	3	8,82	28,21
P63104	14-3-3 protein zeta/delta OS=Homo sapiens OX=9606 GN=YWHAZ PE=1 SV=1	54,4	1	1	1	3,12	6,12	121,43	2	2	2	6,25	12,24						
P63173	60S ribosomal protein L38 OS=Homo sapiens OX=9606 GN=RPL38 PE=1 SV=2	113,5	2	2	2	9,52	32,86	82,54	1	1	1	4,76	18,57	73,2	1	1	1	4,76	18,57
P63220	40S ribosomal protein S21 OS=Homo sapiens OX=9606 GN=RPS21 PE=1 SV=1	62,31	1	1	1	7,69	12,05	62,31	1	1	1	7,69	12,05						
P63244	Receptor of activated protein C kinase 1 OS=Homo sapiens OX=9606 GN=RACK1 PE=1 SV=3	313,92	20	17	17	54,84	61,51	253,45	11	10	10	32,26	43,85	263,13	16	13	13	41,94	52,05
P63261	Actin, cytoplasmic 2 OS=Homo sapiens OX=9606 GN=ACTG1 PE=1 SV=1	457,24	186	35	12	94,59	81,87	436,4	108	26	13	70,27	76,53	380,39	212	32	10	86,49	84,27
P67775	Serine/threonine-protein phosphatase 2A catalytic subunit alpha isoform OS=Homo sapiens OX=9606 GN=PPP2CA PE=1 SV=1	146,2	3	3	3	9,09	9,71							59,57	1	1	1	3,03	2,27

P67809	Y-box-binding protein 1 OS=Homo sapiens OX=9606 GN=YBX1 PE=1 SV=3	311,13	18	11	8	22,92	47,84	260,26	13	7	6	14,58	47,84	273,68	21	11	8	22,92	47,84
P67870	Casein kinase II subunit beta OS=Homo sapiens OX=9606 GN=CSNK2B PE=1 SV=1	200,64	10	5	5	29,41	39,07	180,38	3	3	3	17,65	17,21	150,28	5	4	4	23,53	35,35
P68032	Actin, alpha cardiac muscle 1 OS=Homo sapiens OX=9606 GN=ACTC1 PE=1 SV=1	371,94	114	20	2	54,05	35,54	354,8	51	15	2	40,54	30,5	318,68	111	21	3	56,76	46,95
P68104	Elongation factor 1-alpha 1 OS=Homo sapiens OX=9606 GN=EEF1A1 PE=1 SV=1	345,06	44	19	19	31,67	60,39	284,94	15	9	9	15	32,9	286,39	25	15	15	25	43,07
P68133	Actin, alpha skeletal muscle OS=Homo sapiens OX=9606 GN=ACTA1 PE=1 SV=1	371,94	114	20	2	54,05	35,54	354,8	51	15	2	40,54	30,5						
P68363	Tubulin alpha-1B chain OS=Homo sapiens OX=9606 GN=TUBA1B PE=1 SV=1							387,95	61	20	1	50	62,31	349,58	76	23	1	57,5	71,18
P68371	Tubulin beta-4B chain OS=Homo sapiens OX=9606 GN=TUBB4B PE=1 SV=1	471,92	129	34	1	91,89	83,6	430,58	63	30	2	81,08	83,82	374,81	93	28	1	75,68	79,55
P68400	Casein kinase II subunit alpha OS=Homo sapiens OX=9606 GN=CSNK2A1 PE=1 SV=1	288,27	22	13	13	27,66	42,2	250,78	12	8	8	17,02	30,95	167,95	6	4	4	8,51	16,62
P78527	DNA-dependent protein kinase catalytic subunit OS=Homo sapiens OX=9606 GN=PRKDC PE=1 SV=3	240,77	19	15	15	3,12	4,82	67,87	1	1	1	0,21	0,39	215,34	12	9	9	1,88	3,44
P81605	Dermcidin OS=Homo sapiens OX=9606 GN=DCD PE=1 SV=2	157,99	3	3	3	21,43	35,45	114,94	2	2	2	14,29	22,73	141,19	4	3	3	21,43	35,45
P83731	60S ribosomal protein L24 OS=Homo sapiens OX=9606 GN=RPL24 PE=1 SV=1	185,19	5	4	4	9,52	23,57							82,83	2	2	2	4,76	12,74
P83881	60S ribosomal protein L36a OS=Homo sapiens OX=9606 GN=RPL36A PE=1 SV=2	74,89	2	2	2	6,06	15,09							80,72	1	1	1	3,03	8,49
P84098	60S ribosomal protein L19 OS=Homo sapiens OX=9606 GN=RPL19 PE=1 SV=1	109,05	5	3	3	4,84	9,18	81,2	2	1	1	1,61	4,08	73,41	2	2	2	3,23	8,67
P84103	Serine/arginine-rich splicing factor 3 OS=Homo sapiens OX=9606 GN=SRSF3 PE=1 SV=1	165,86	6	5	5	11,63	32,32	187,64	4	4	4	9,3	27,44	124,84	2	2	2	4,65	18,29

P84243	Histone H3.3 OS=Homo sapiens OX=9606 GN=H3-3A PE=1 SV=2							142,07	9	4	1	13,33	43,38	179,85	23	6	1	20	48,53
Q00325	Phosphate carrier protein, mitochondrial OS=Homo sapiens OX=9606 GN=SLC25A3 PE=1 SV=2	106,11	3	3	3	7,5	7,73							59,59	1	1	1	2,5	3,31
Q00839	Heterogeneous nuclear ribonucleoprotein U OS=Homo sapiens OX=9606 GN=HNRNPU PE=1 SV=6	230	10	9	9	8,18	15,76	202,63	6	6	6	5,45	12,36	218,97	10	8	8	7,27	11,39
Q01081	Splicing factor U2AF 35 kDa subunit OS=Homo sapiens OX=9606 GN=U2AF1 PE=1 SV=3	104,21	1	1	1	2,22	5,42	53,62	1	1	1	2,22	5,83	74,13	1	1	1	2,22	5,42
Q01130	Serine/arginine-rich splicing factor 2 OS=Homo sapiens OX=9606 GN=SRSF2 PE=1 SV=4	51,8	1	1	1	1,56	3,62	147,69	4	4	4	6,25	16,74	58,2	1	1	1	1,56	3,62
Q01650	Large neutral amino acids transporter small subunit 1 OS=Homo sapiens OX=9606 GN=SLC7A5 PE=1 SV=2	127,64	3	2	2	6,9	3,16	72,72	1	1	1	3,45	3,16	117	2	2	2	6,9	3,16
Q02413	Desmoglein-1 OS=Homo sapiens OX=9606 GN=DSG1 PE=1 SV=2	81,57	1	1	1	1,33	1,33	152,07	3	3	3	4	4,67	177,88	5	5	5	6,67	7,44
Q02543	60S ribosomal protein L18a OS=Homo sapiens OX=9606 GN=RPL18A PE=1 SV=2	212,67	9	6	6	16,67	40,91	107,82	2	2	2	5,56	14,77	186,27	6	5	5	13,89	27,27
Q02878	60S ribosomal protein L6 OS=Homo sapiens OX=9606 GN=RPL6 PE=1 SV=3	94,45	2	2	2	2,94	6,6	127,55	2	2	2	2,94	10,42	119,12	3	3	3	4,41	11,81
Q03001	Dystonin OS=Homo sapiens OX=9606 GN=DST PE=1 SV=4	47,96	2	1	1	0,11	0,16							50,47	2	1	1	0,11	0,16
Q03135	Caveolin-1 OS=Homo sapiens OX=9606 GN=CAV1 PE=1 SV=4							177,37	4	3	3	17,65	29,21	110,6	2	2	2	11,76	19,66
Q04695	Keratin, type I cytoskeletal 17 OS=Homo sapiens OX=9606 GN=KRT17 PE=1 SV=2	282,6	20	12	1	23,08	24,54	272,88	19	12	3	23,08	26,85	273,12	35	20	7	38,46	39,58
Q04760	Lactoylglutathione lyase OS=Homo sapiens OX=9606 GN=GLO1 PE=1 SV=4							55,97	1	1	1	4,35	3,8	49,54	1	1	1	4,35	3,8
Q04837	Single-stranded DNA-binding protein, mitochondrial OS=Homo sapiens OX=9606 GN=SSBP1 PE=1 SV=1	61,26	1	1	1	4,55	5,41	90,04	1	1	1	4,55	10,14	81,81	1	1	1	4,55	10,14
Q06830	Peroxiredoxin-1 OS=Homo sapiens OX=9606 GN=PRDX1 PE=1 SV=1	64,37	2	1	1	4,35	4,52	99,81	2	2	2	8,7	13,57						

Q07020	60S ribosomal protein L18 OS=Homo sapiens OX=9606 GN=RPL18 PE=1 SV=2	161,35	7	3	3	6,25	19,68	79,79	1	1	1	2,08	7,45	138,08	6	3	3	6,25	19,68
Q07021	Complement component 1 Q subcomponent-binding protein, mitochondrial OS=Homo sapiens OX=9606 GN=C1QBP PE=1 SV=1	99,66	1	1	1	3,45	9,57	92,43	1	1	1	3,45	9,57	135,17	3	2	2	6,9	20,21
Q07157	Tight junction protein ZO-1 OS=Homo sapiens OX=9606 GN=TJP1 PE=1 SV=3	443,93	74	45	45	23,32	37,36	413,61	38	32	32	16,58	30,32	346,94	36	29	29	15,03	27,23
Q07666	KH domain-containing, RNA-binding, signal transduction-associated protein 1 OS=Homo sapiens OX=9606 GN=KHDRBS1 PE=1 SV=1	105,33	2	1	1	1,75	3,16	68,55	1	1	1	1,75	3,16	73,35	1	1	1	1,75	3,16
Q07955	Serine/arginine-rich splicing factor 1 OS=Homo sapiens OX=9606 GN=SRSF1 PE=1 SV=2	133,04	4	3	2	5,77	16,13	219,51	9	5	5	9,62	25,81	182,77	7	6	5	11,54	29,03
Q08211	ATP-dependent RNA helicase A OS=Homo sapiens OX=9606 GN=DHX9 PE=1 SV=4	160,58	5	5	5	3,68	6,38	55,45	1	1	1	0,74	0,94	191,9	8	8	8	5,88	7,72
Q08554	Desmocollin-1 OS=Homo sapiens OX=9606 GN=DSC1 PE=1 SV=2	103,18	2	2	2	2,2	3,36	123,63	3	2	2	2,2	3,36	129,06	3	3	3	3,3	4,92
Q09666	Neuroblast differentiation-associated protein AHNAK OS=Homo sapiens OX=9606 GN=AHNAK PE=1 SV=2	88,74	2	2	2	0,23	0,31	239,39	11	10	10	1,16	2,46						
Q12905	Interleukin enhancer-binding factor 2 OS=Homo sapiens OX=9606 GN=ILF2 PE=1 SV=2	113,58	3	3	3	7,89	10,26	124,25	2	2	2	5,26	9,49	129,16	4	4	4	10,53	14,36
Q12906	Interleukin enhancer-binding factor 3 OS=Homo sapiens OX=9606 GN=ILF3 PE=1 SV=3	110,79	2	2	2	2,11	2,8	55,67	1	1	1	1,05	1,34						
Q13200	26S proteasome non-ATPase regulatory subunit 2 OS=Homo sapiens OX=9606 GN=PSMD2 PE=1 SV=3	156,01	6	6	6	6,82	8,7	103,35	1	1	1	1,14	1,65	123,13	3	3	3	3,41	5,4
Q13242	Serine/arginine-rich splicing factor 9 OS=Homo sapiens OX=9606 GN=SRSF9 PE=1 SV=1	70,79	2	2	1	5,26	7,69	87,24	2	1	1	2,63	4,52	78,33	2	2	1	5,26	7,69
Q13243	Serine/arginine-rich splicing factor 5 OS=Homo sapiens OX=9606 GN=SRSF5 PE=1 SV=1	84,32	1	1	1	1,39	3,31	116,03	2	2	1	2,78	8,46						

Q13247	Serine/arginine-rich splicing factor 6 OS=Homo sapiens OX=9606 GN=SRSF6 PE=1 SV=2							99,04	2	2	1	2,13	6,69	109,77	2	2	2	2,13	6,69
Q13268	Dehydrogenase/reductase SDR family member 2, mitochondrial OS=Homo sapiens OX=9606 GN=DHRS2 PE=1 SV=4	204,94	8	7	7	22,58	31,79	223,39	10	8	8	25,81	37,86	225,26	11	10	10	32,26	56,07
Q13283	Ras GTPase-activating protein-binding protein 1 OS=Homo sapiens OX=9606 GN=G3BP1 PE=1 SV=1	302,58	27	14	13	29,17	37,55	216,22	10	7	7	14,58	21,67	181,68	8	5	5	10,42	17,81
Q13310	Polyadenylate-binding protein 4 OS=Homo sapiens OX=9606 GN=PABPC4 PE=1 SV=1	306,07	19	17	9	21,25	31,99	246,37	9	9	5	11,25	17,7	232,56	9	9	3	11,25	15,68
Q13435	Splicing factor 3B subunit 2 OS=Homo sapiens OX=9606 GN=SF3B2 PE=1 SV=2	212,15	7	7	7	5,74	11,06	149,16	3	3	3	2,46	7,71	88,03	2	2	2	1,64	3,58
Q13501	Sequestosome-1 OS=Homo sapiens OX=9606 GN=SQSTM1 PE=1 SV=1	153,16	2	2	2	5	8,64	97,21	1	1	1	2,5	5,91	152,03	3	3	3	7,5	8,86
Q13671	Ras and Rab interactor 1 OS=Homo sapiens OX=9606 GN=RIN1 PE=1 SV=4	193,64	5	5	5	6,58	7,02							98,06	2	2	2	2,63	2,68
Q14103	Heterogeneous nuclear ribonucleoprotein D0 OS=Homo sapiens OX=9606 GN=HNRNPD PE=1 SV=1	180,75	4	4	3	8,7	15,77	165,52	4	4	3	8,7	14,93	168,21	6	5	3	10,87	17,75
Q14137	Ribosome biogenesis protein BOP1 OS=Homo sapiens OX=9606 GN=BOP1 PE=1 SV=2	55,84	1	1	1	1,22	3,49	51,48	1	1	1	1,22	3,49	81,1	2	2	2	2,44	7,64
Q14152	Eukaryotic translation initiation factor 3 subunit A OS=Homo sapiens OX=9606 GN=EIF3A PE=1 SV=1	165,06	6	6	6	2,02	5,07	58,69	1	1	1	0,34	0,72	69,01	1	1	1	0,34	0,72
Q14157	Ubiquitin-associated protein 2-like OS=Homo sapiens OX=9606 GN=UBAP2L PE=1 SV=2	137,71	6	6	6	8,11	7,18	52,77	1	1	1	1,35	0,83						
Q14192	Four and a half LIM domains protein 2 OS=Homo sapiens OX=9606 GN=FHL2 PE=1 SV=3	278,85	14	10	10	27,78	43,01	132,59	3	3	3	8,33	12,9	223,47	12	11	11	30,56	51,61
Q14247	Src substrate cortactin OS=Homo sapiens OX=9606 GN=CTTN PE=1 SV=2	80,67	3	2	2	2,6	4,55	144,58	2	2	2	2,6	4,18	122,62	5	4	4	5,19	7,82
Q14254	Flotillin-2 OS=Homo sapiens OX=9606 GN=FLOT2 PE=1 SV=2							135,93	3	3	3	5,45	9,81	212,18	9	9	9	16,36	29,67

Q14315	Filamin-C OS=Homo sapiens OX=9606 GN=FLNC PE=1 SV=3	340,72	33	29	27	11,15	13,03							147,49	5	5	5	1,92	2,06
Q14739	Delta(14)-sterol reductase LBR OS=Homo sapiens OX=9606 GN=LBR PE=1 SV=2	230,69	10	7	7	9,72	13,66	125,39	3	3	3	4,17	3,9	166,39	6	5	5	6,94	7,15
Q14764	Major vault protein OS=Homo sapiens OX=9606 GN=MVP PE=1 SV=4	298,94	23	17	17	16,19	32,03	390,87	42	25	25	23,81	47,7	404,29	91	42	42	40	68,98
Q14974	Importin subunit beta-1 OS=Homo sapiens OX=9606 GN=KPNB1 PE=1 SV=2							86,72	1	1	1	1,45	1,37	54,76	1	1	1	1,45	1,37
Q15084	Protein disulfide-isomerase A6 OS=Homo sapiens OX=9606 GN=PDIA6 PE=1 SV=1	43,3	1	1	1	2,04	2,27	153,56	4	4	4	8,16	13,18						
Q15149	Plectin OS=Homo sapiens OX=9606 GN=PLEC PE=1 SV=3	480,51	118	106	106	15,38	27,95	436,48	74	68	68	9,87	20,43	442,94	132	116	116	16,84	32,3
Q15233	Non-POU domain-containing octamer-binding protein OS=Homo sapiens OX=9606 GN=NONO PE=1 SV=4	164,01	6	4	4	5,71	12,95	97,57	1	1	1	1,43	4,88	81,29	1	1	1	1,43	4,88
Q15393	Splicing factor 3B subunit 3 OS=Homo sapiens OX=9606 GN=SF3B3 PE=1 SV=4	179,97	5	5	5	4,39	8,22							128,43	4	3	3	2,63	5,01
Q15459	Splicing factor 3A subunit 1 OS=Homo sapiens OX=9606 GN=SF3A1 PE=1 SV=1	151,6	5	5	5	5,81	6,94	135,64	3	3	3	3,49	4,92						
Q15717	ELAV-like protein 1 OS=Homo sapiens OX=9606 GN=ELAVL1 PE=1 SV=2	67,27	1	1	1	2,78	3,37	66,19	1	1	1	2,78	3,37						
Q16181	Septin-7 OS=Homo sapiens OX=9606 GN=SEPTIN7 PE=1 SV=2	212,83	16	10	9	13,89	23,57	196,89	10	7	6	9,72	19,91	182,33	9	7	6	9,72	21,97
Q16629	Serine/arginine-rich splicing factor 7 OS=Homo sapiens OX=9606 GN=SRSF7 PE=1 SV=1	134,31	3	3	3	4,76	14,71	177,88	4	4	4	6,35	24,37	142,86	4	4	4	6,35	23,53
Q16643	Drebrin OS=Homo sapiens OX=9606 GN=DBN1 PE=1 SV=4	101,42	2	2	2	3,51	3,7	172,06	3	3	3	5,26	9,24	192,52	5	5	5	8,77	13,87
Q16777	Histone H2A type 2-C OS=Homo sapiens OX=9606 GN=H2AC20 PE=1 SV=4	270,23	38	9	5	34,62	58,91	255,39	33	7	3	26,92	58,91	219,66	38	6	1	23,08	58,14
Q16778	Histone H2B type 2-E OS=Homo sapiens OX=9606 GN=H2BC21 PE=1 SV=3	234,71	19	9	3	32,14	51,59	225,76	13	5	2	17,86	32,54	182,56	16	6	1	21,43	42,86

Q16891	MICOS complex subunit MIC60 OS=Homo sapiens OX=9606 GN=IMMT PE=1 SV=1	124,46	3	3	3	3,23	5,15	114,79	1	1	1	1,08	1,72	132,47	4	4	4	4,3	6,86
Q3YEC7	Rab-like protein 6 OS=Homo sapiens OX=9606 GN=RABL6 PE=1 SV=2	385,42	46	23	23	25,27	32,78	259,11	10	8	8	8,79	18,24	297,2	26	16	16	17,58	25,65
Q562R1	Beta-actin-like protein 2 OS=Homo sapiens OX=9606 GN=ACTBL2 PE=1 SV=2	240,7	51	9	1	23,68	16,76	240,47	13	5	1	13,16	14,1						
Q5D862	Filaggrin-2 OS=Homo sapiens OX=9606 GN=FLG2 PE=1 SV=1	124,64	2	2	2	1,17	1,25	145,13	3	3	3	1,75	2,3	177,62	6	5	5	2,92	3,76
Q5JNZ5	Putative 40S ribosomal protein S26-like 1 OS=Homo sapiens OX=9606 GN=RPS26P11 PE=5 SV=1	142,18	6	3	3	12,5	31,3	87,53	3	1	1	4,17	13,04						
Q5QNW6	Histone H2B type 2-F OS=Homo sapiens OX=9606 GN=H2BC18 PE=1 SV=3	240,51	20	9	3	32,14	51,59	239,22	15	6	3	21,43	32,54	193,26	19	7	2	25	42,86
Q5SW79	Centrosomal protein of 170 kDa OS=Homo sapiens OX=9606 GN=CEP170 PE=1 SV=1	400,14	50	36	36	16,44	30,43	161,02	4	3	3	1,37	2,97	213,11	11	8	8	3,65	7,2
Q5T5X7	BEN domain-containing protein 3 OS=Homo sapiens OX=9606 GN=BEND3 PE=1 SV=1	301,62	16	11	11	10,58	21,38	208,97	8	7	7	6,73	13,65	225,85	9	7	7	6,73	17,03
Q5T749	Keratinocyte proline-rich protein OS=Homo sapiens OX=9606 GN=KPRP PE=1 SV=1	87,24	1	1	1	2,13	1,9	79,1	1	1	1	2,13	1,9	111,48	2	1	1	2,13	1,9
Q5T7W0	Zinc finger protein 618 OS=Homo sapiens OX=9606 GN=ZNF618 PE=1 SV=1	154,05	5	5	5	4,9	10,38							138,14	3	3	3	2,94	4,72
Q5VTE0	Putative elongation factor 1-alpha-like 3 OS=Homo sapiens OX=9606 GN=EEF1A1P5 PE=5 SV=1							284,94	15	9	9	14,75	32,9	286,39	25	15	15	24,59	43,07
Q5VUA4	Zinc finger protein 318 OS=Homo sapiens OX=9606 GN=ZNF318 PE=1 SV=2	269,44	23	17	17	5,69	9,57							154,05	6	5	5	1,67	2,19
Q6FI13	Histone H2A type 2-A OS=Homo sapiens OX=9606 GN=H2AC18 PE=1 SV=3	270,23	38	9	5	34,62	58,46	255,39	33	7	3	26,92	58,46	219,66	38	6	1	23,08	57,69
Q6NYC8	Phostensin OS=Homo sapiens OX=9606 GN=PPP1R18 PE=1 SV=1	245,02	10	10	10	12,2	26,43	214,69	8	8	8	9,76	23,49	181,35	5	5	5	6,1	11,58

Q6NZI2	Caveolae-associated protein 1 OS=Homo sapiens OX=9606 GN=CAVIN1 PE=1 SV=1	226,75	11	6	6	8,96	15,9	201,24	7	5	5	7,46	14,87	170,27	5	4	4	5,97	15,38
Q6UWP8	Suprabasin OS=Homo sapiens OX=9606 GN=SBSN PE=1 SV=2	83,84	2	2	2	4	5,42	187,45	4	3	3	6	8,47	131,81	3	2	2	4	6,1
Q6WCQ1	Myosin phosphatase Rho-interacting protein OS=Homo sapiens OX=9606 GN=MPRIP PE=1 SV=3							136,9	2	2	2	1,32	4,78	123,38	2	2	2	1,32	3,22
Q6ZVX7	F-box only protein 50 OS=Homo sapiens OX=9606 GN=NCCRP1 PE=1 SV=1	66,09	1	1	1	3,57	4							68,21	1	1	1	3,57	4
Q71DI3	Histone H3.2 OS=Homo sapiens OX=9606 GN=H3C15 PE=1 SV=3	165,36	13	5	5	16,67	48,53	185,49	10	4	1	13,33	43,38	190,52	34	6	1	20	48,53
Q71RC2	La-related protein 4 OS=Homo sapiens OX=9606 GN=LARP4 PE=1 SV=3	346,68	28	17	17	22,97	26,93	244,75	9	9	9	12,16	19,2	199,41	8	8	8	10,81	16,02
Q71U36	Tubulin alpha-1A chain OS=Homo sapiens OX=9606 GN=TUBA1A PE=1 SV=1							386,89	55	20	1	50	62,31	348,87	69	23	1	57,5	71,18
Q71UI9	Histone H2A.V OS=Homo sapiens OX=9606 GN=H2AZ2 PE=1 SV=3	195,52	7	4	3	16,67	53,91	192,11	8	4	3	16,67	53,91	175,61	10	4	3	16,67	53,91
Q7KZI7	Serine/threonine-protein kinase MARK2 OS=Homo sapiens OX=9606 GN=MARK2 PE=1 SV=2	41,44	1	1	1	0,91	1,14							78,1	1	1	1	0,91	2,54
Q7L7L0	Histone H2A type 3 OS=Homo sapiens OX=9606 GN=H2AW PE=1 SV=3	196,65	15	5	1	19,23	36,15	209,66	15	5	1	19,23	36,15	193,19	22	6	2	23,08	57,69
Q86TI0	TBC1 domain family member 1 OS=Homo sapiens OX=9606 GN=TBC1D1 PE=1 SV=2	229,55	13	9	9	6,47	10,19	71,68	1	1	1	0,72	1,03	131,65	5	3	3	2,16	3,08
Q86UE8	Serine/threonine-protein kinase tousled-like 2 OS=Homo sapiens OX=9606 GN=TLK2 PE=1 SV=2	80,71	1	1	1	0,93	1,04							67,83	1	1	1	0,93	2,2
Q86V48	Leucine zipper protein 1 OS=Homo sapiens OX=9606 GN=LUZP1 PE=1 SV=2	125,02	4	3	3	1,75	3,16	77,4	1	1	1	0,58	1,21						
Q86V81	THO complex subunit 4 OS=Homo sapiens OX=9606 GN=ALYREF PE=1 SV=3	93,05	2	2	2	5,13	14,79	84,69	1	1	1	2,56	4,28	65,67	1	1	1	2,56	4,28
Q86YZ3	Hornerin OS=Homo sapiens OX=9606 GN=HRNR PE=1 SV=2	267,23	13	7	7	3,83	5,37	281,81	13	6	6	3,28	3,65	239,91	15	8	8	4,37	5,51

Q8IUD2	ELKS/Rab6-interacting/CAST family member 1 OS=Homo sapiens OX=9606 GN=ERC1 PE=1 SV=1	300,42	22	16	16	8,6	18,01	100,19	2	2	2	1,08	3,49	161,44	5	4	4	2,15	5,29
Q8IUE6	Histone H2A type 2-B OS=Homo sapiens OX=9606 GN=H2AC21 PE=1 SV=3	156,47	8	5	2	20,83	35,38	163,82	9	4	1	16,67	35,38						
Q8IWX8	Calcium homeostasis endoplasmic reticulum protein OS=Homo sapiens OX=9606 GN=CHERP PE=1 SV=3	59,74	1	1	1	0,99	1,42	58,33	1	1	1	0,99	1,42						
Q8N357	Solute carrier family 35 member F6 OS=Homo sapiens OX=9606 GN=SLC35F6 PE=1 SV=1	82,47	1	1	1	4,55	3,23							74,77	1	1	1	4,55	3,23
Q8N3V7	Synaptopodin OS=Homo sapiens OX=9606 GN=SYNPO PE=1 SV=2	109,37	3	2	2	2,47	3,98	59,4	1	1	1	1,23	2,05	89,52	2	2	2	2,47	3,98
Q8N8S7	Protein enabled homolog OS=Homo sapiens OX=9606 GN=ENAH PE=1 SV=2	192,45	7	6	6	6,98	15,74	97,53	2	1	1	1,16	3,72	90,65	2	1	1	1,16	3,72
Q8NB37	Glutamine amidotransferase-like class 1 domain-containing protein 1 OS=Homo sapiens OX=9606 GN=GATD1 PE=1 SV=1	265,08	8	6	6	40	48,64	194,65	4	4	4	26,67	28,18	207,11	6	6	6	40	50,45
Q8NB90	ATPase family protein 2 homolog OS=Homo sapiens OX=9606 GN=SPATA5 PE=1 SV=3	304,82	21	18	17	18,75	28,78	203,29	8	6	6	6,25	10,64	177,33	7	6	6	6,25	9,29
Q8NC51	Plasminogen activator inhibitor 1 RNA-binding protein OS=Homo sapiens OX=9606 GN=SERBP1 PE=1 SV=2	214,5	11	6	6	9,09	17,4	185,99	5	4	4	6,06	11,76	169,79	6	5	5	7,58	14,46
Q8TBC3	SH3KBP1-binding protein 1 OS=Homo sapiens OX=9606 GN=SHKBP1 PE=1 SV=2	284,99	15	12	11	18,75	30,13							216,31	9	7	6	10,94	19,24
Q8TEJ3	E3 ubiquitin-protein ligase SH3RF3 OS=Homo sapiens OX=9606 GN=SH3RF3 PE=1 SV=2	255,1	8	8	8	8,99	14,85							101,83	2	2	2	2,25	4,65
Q8WX93	Palladin OS=Homo sapiens OX=9606 GN=PALLD PE=1 SV=3	310,1	23	17	17	13,08	17,35	277,57	16	13	13	10	13,88	204,61	11	9	9	6,92	9,4
Q92522	Histone H1.10 OS=Homo sapiens OX=9606 GN=H1-10 PE=1 SV=1	102,01	1	1	1	1,96	7,51	122,42	2	2	2	3,92	7,51	149,41	3	2	2	3,92	7,51
Q92614	Unconventional myosin-XVIIIa OS=Homo sapiens OX=9606 GN=MYO18A PE=1 SV=3	507,63	115	78	78	25,24	48,49	417,19	40	36	36	11,65	28,19	379,01	70	53	53	17,15	35

Q92841	Probable ATP-dependent RNA helicase DDX17 OS=Homo sapiens OX=9606 GN=DDX17 PE=1 SV=2	225,28	10	10	5	11,49	13,72	170,67	3	3	2	3,45	5,21	168,4	4	4	2	4,6	6,72
Q92974	Rho guanine nucleotide exchange factor 2 OS=Homo sapiens OX=9606 GN=ARHGEF2 PE=1 SV=4	294	22	19	19	13,48	26,88	177,71	6	6	6	4,26	9,33	194,52	7	7	7	4,96	11,56
Q93077	Histone H2A type 1-C OS=Homo sapiens OX=9606 GN=H2AC6 PE=1 SV=3	196,65	15	5	1	19,23	36,15	209,66	15	5	1	19,23	36,15	193,19	22	6	2	23,08	57,69
Q93079	Histone H2B type 1-H OS=Homo sapiens OX=9606 GN=H2BC9 PE=1 SV=3	240,51	20	9	3	32,14	51,59	239,22	15	6	3	21,43	32,54	193,26	19	7	2	25	42,86
Q969Q0	60S ribosomal protein L36a-like OS=Homo sapiens OX=9606 GN=RPL36AL PE=1 SV=3	74,89	2	2	2	6,06	15,09							80,72	1	1	1	3,03	8,49
Q969V6	Myocardin-related transcription factor A OS=Homo sapiens OX=9606 GN=MRTFA PE=1 SV=1	223,71	8	7	7	8,86	14,07	59,64	1	1	1	1,27	3,11	63,19	1	1	1	1,27	3,11
Q96EY1	DnaJ homolog subfamily A member 3, mitochondrial OS=Homo sapiens OX=9606 GN=DNAJA3 PE=1 SV=2	44,66	1	1	1	1,56	4,79	56,93	1	1	1	1,56	4,79	69,64	1	1	1	1,56	4,79
Q96GY0	Zinc finger C2HC domain-containing protein 1A OS=Homo sapiens OX=9606 GN=ZC2HC1A PE=1 SV=2	306,78	34	17	17	36,17	44,62	221,36	11	7	7	14,89	22,77	223,53	16	11	11	23,4	31,69
Q96HS1	Serine/threonine-protein phosphatase PGAM5, mitochondrial OS=Homo sapiens OX=9606 GN=PGAM5 PE=1 SV=2	214,82	9	7	7	20,59	24,22	71,67	1	1	1	2,94	5,19	101,15	2	2	2	5,88	7,27
Q96HU8	GTP-binding protein Di-Ras2 OS=Homo sapiens OX=9606 GN=DIRAS2 PE=1 SV=1							54,35	2	1	1	3,33	5,53	50,44	2	1	1	3,33	5,53
Q96I25	Splicing factor 45 OS=Homo sapiens OX=9606 GN=RBM17 PE=1 SV=1	72,34	2	1	1	1,52	3,24	56,33	1	1	1	1,52	3,24						
Q96II8	DISP complex protein LRCH3 OS=Homo sapiens OX=9606 GN=LRCH3 PE=1 SV=2	330,2	25	19	18	24,36	35,26	175,07	6	5	5	6,41	8,49	238,57	14	11	11	14,1	23,68
Q96N67	Dedicator of cytokinesis protein 7 OS=Homo sapiens OX=9606 GN=DOCK7 PE=1 SV=4	478,62	114	72	72	30,38	42,8	343,72	33	27	27	11,39	18,27	380,04	73	55	55	23,21	35,28

Q96NY7	Chloride intracellular channel protein 6 OS=Homo sapiens OX=9606 GN=CLIC6 PE=2 SV=3	60,21	1	1	1	1,59	1,42							55,07	1	1	1	1,59	1,42
Q96P16	Regulation of nuclear pre-mRNA domain-containing protein 1A OS=Homo sapiens OX=9606 GN=RPRD1A PE=1 SV=1	269,82	12	10	7	20,83	41,67	121,71	2	2	2	4,17	13,78	180,72	5	5	2	10,42	18,91
Q96P70	Importin-9 OS=Homo sapiens OX=9606 GN=IPO9 PE=1 SV=3	61,09	1	1	1	1,33	2,69							70,02	2	1	1	1,33	2,69
Q96PK6	RNA-binding protein 14 OS=Homo sapiens OX=9606 GN=RBM14 PE=1 SV=2	124,52	3	3	3	5,26	5,53	137,62	2	2	2	3,51	5,38	128,43	3	3	3	5,26	6,13
Q96SN8	CDK5 regulatory subunit-associated protein 2 OS=Homo sapiens OX=9606 GN=CDK5RAP2 PE=1 SV=5	178,73	6	6	6	2,36	5,6							81,67	3	2	2	0,79	1,16
Q99623	Prohibitin-2 OS=Homo sapiens OX=9606 GN=PHB2 PE=1 SV=2	88,38	1	1	1	2,44	4,01	307,91	22	16	16	39,02	60,87	250,99	17	12	12	29,27	46,15
Q99735	Microsomal glutathione S-transferase 2 OS=Homo sapiens OX=9606 GN=MGST2 PE=1 SV=1	87,84	1	1	1	6,25	9,52							65,95	1	1	1	6,25	9,52
Q99759	Mitogen-activated protein kinase kinase kinase 3 OS=Homo sapiens OX=9606 GN=MAP3K3 PE=1 SV=2	143,72	5	4	4	4,94	9,27							51,83	1	1	1	1,23	1,76
Q99877	Histone H2B type 1-N OS=Homo sapiens OX=9606 GN=H2BC15 PE=1 SV=3	240,51	20	9	3	32,14	51,59	239,22	15	6	3	21,43	32,54	193,26	19	7	2	25	42,86
Q99879	Histone H2B type 1-M OS=Homo sapiens OX=9606 GN=H2BC14 PE=1 SV=3	240,51	20	9	3	32,14	51,59	239,22	15	6	3	21,43	32,54	193,26	19	7	2	25	42,86
Q99880	Histone H2B type 1-L OS=Homo sapiens OX=9606 GN=H2BC13 PE=1 SV=3	240,51	20	9	3	32,14	51,59	239,22	15	6	3	21,43	32,54	193,26	19	7	2	25	42,86
Q9BQE3	Tubulin alpha-1C chain OS=Homo sapiens OX=9606 GN=TUBA1C PE=1 SV=1	418,63	82	26	2	65	66,15	373,52	58	19	1	47,5	58,8	340,24	73	22	1	55	67,71
Q9BRL6	Serine/arginine-rich splicing factor 8 OS=Homo sapiens OX=9606 GN=SRSF8 PE=1 SV=1	51,8	1	1	1	1,37	2,84							58,2	1	1	1	1,37	2,84
Q9BUF5	Tubulin beta-6 chain OS=Homo sapiens OX=9606 GN=TUBB6 PE=1 SV=1	319,75	46	13	3	36,11	39,69	282,58	16	10	1	27,78	31,84	278,88	36	14	3	38,89	42,38

Q9BUJ2	Heterogeneous nuclear ribonucleoprotein U-like protein 1 OS=Homo sapiens OX=9606 GN=HNRNPUL1 PE=1 SV=2	270,27	9	9	9	9,28	14,72	158,48	3	3	3	3,09	5,96	213,31	7	7	7	7,22	10,51
Q9BVQ7	Spermatogenesis-associated protein 5-like protein 1 OS=Homo sapiens OX=9606 GN=SPATA5L1 PE=1 SV=2	262,42	15	11	11	13,41	23,51	84,62	2	1	1	1,22	2,52	112,08	4	2	2	2,44	4,12
Q9BW62	Katanin p60 ATPase-containing subunit A-like 1 OS=Homo sapiens OX=9606 GN=KATNAL1 PE=1 SV=1							66,9	1	1	1	1,37	2,45	57,87	1	1	1	1,37	2,45
Q9BXP5	Serrate RNA effector molecule homolog OS=Homo sapiens OX=9606 GN=SRRT PE=1 SV=1	108,51	3	3	3	2,16	5,14							104,8	3	3	3	2,16	5,25
Q9C0C2	182 kDa tankyrase-1-binding protein OS=Homo sapiens OX=9606 GN=TNKS1BP1 PE=1 SV=4	96,64	1	1	1	0,69	0,98	83,55	1	1	1	0,69	0,98						
Q9GZQ8	Microtubule-associated proteins 1A/1B light chain 3B OS=Homo sapiens OX=9606 GN=MAP1LC3B PE=1 SV=3	182,47	10	7	7	35	38,4	162,65	4	4	4	20	30,4	100,8	3	2	2	10	19,2
Q9H6R4	Nucleolar protein 6 OS=Homo sapiens OX=9606 GN=NOL6 PE=1 SV=2	47,32	1	1	1	0,89	2,71							85	2	1	1	0,89	2,71
Q9H9B4	Sideroflexin-1 OS=Homo sapiens OX=9606 GN=SFXN1 PE=1 SV=4	157,34	5	4	4	12,9	20,19	107,1	1	1	1	3,23	10,56	77,68	2	2	2	6,45	13,66
Q9NQG5	Regulation of nuclear pre-mRNA domain-containing protein 1B OS=Homo sapiens OX=9606 GN=RPRD1B PE=1 SV=1	325,71	30	17	14	36,17	63,5	235,52	10	7	7	14,89	36,81	258,36	19	12	9	25,53	49,69
Q9NQW6	Anillin OS=Homo sapiens OX=9606 GN=ANLN PE=1 SV=2	262	13	12	12	8,22	13,17	99,89	1	1	1	0,68	0,8						
Q9NUQ6	SPATS2-like protein OS=Homo sapiens OX=9606 GN=SPATS2L PE=1 SV=2	234,33	7	7	7	8,24	17,03							119,56	2	2	2	2,35	4,12
Q9NVA2	Septin-11 OS=Homo sapiens OX=9606 GN=SEPTIN11 PE=1 SV=3	137,72	5	4	2	6,25	10,96	162,85	5	4	3	6,25	13,05	90,16	3	3	1	4,69	8,39
Q9NX04	Uncharacterized protein C1orf109 OS=Homo sapiens OX=9606 GN=C1orf109 PE=1 SV=1	77,63	2	1	1	4	5,42	69,05	1	1	1	4	5,42	63,16	1	1	1	4	5,42

Q9NYF8	Bcl-2-associated transcription factor 1 OS=Homo sapiens OX=9606 GN=BCLAF1 PE=1 SV=2	61,21	1	1	1	0,5	0,98	95,91	2	2	2	1	2,28	109,74	3	2	2	1	2,28
Q9NYL9	Tropomodulin-3 OS=Homo sapiens OX=9606 GN=TMOD3 PE=1 SV=1							71,57	1	1	1	2,27	3,12	88,59	1	1	1	2,27	3,12
Q9NZB2	Constitutive coactivator of PPAR-gamma-like protein 1 OS=Homo sapiens OX=9606 GN=FAM120A PE=1 SV=2	141,59	2	2	2	1,75	3,85							65,63	1	1	1	0,88	1,61
Q9P0K7	Ankycorbin OS=Homo sapiens OX=9606 GN=RAI14 PE=1 SV=2							130,48	3	3	3	2,21	4,8	55,09	1	1	1	0,74	1,43
Q9P0M6	Core histone macro-H2A.2 OS=Homo sapiens OX=9606 GN=MACROH2A2 PE=1 SV=3							58,99	1	1	1	1,64	7,8	52,15	1	1	1	1,64	2,42
Q9P2E9	Ribosome-binding protein 1 OS=Homo sapiens OX=9606 GN=RRBP1 PE=1 SV=5	72,36	2	2	2	0,88	1,91							54,13	1	1	1	0,44	0,92
Q9P2M7	Cingulin OS=Homo sapiens OX=9606 GN=CGN PE=1 SV=2	46,46	1	1	1	0,5	0,84							52,64	1	1	1	0,5	0,84
Q9UBX3	Mitochondrial dicarboxylate carrier OS=Homo sapiens OX=9606 GN=SLC25A10 PE=1 SV=2	76,67	1	1	1	3,03	8,36							57,47	1	1	1	3,03	3,48
Q9UDY2	Tight junction protein ZO-2 OS=Homo sapiens OX=9606 GN=TJP2 PE=1 SV=2	264,96	18	14	14	8,14	15,88	100,56	2	1	1	0,58	0,76	102,19	5	3	3	1,74	4,37
Q9UHB6	LIM domain and actin-binding protein 1 OS=Homo sapiens OX=9606 GN=LIMA1 PE=1 SV=1	142,1	4	4	4	3,67	4,61	151,53	4	4	4	3,67	7,77	173,87	5	5	5	4,59	9,75
Q9UHD8	Septin-9 OS=Homo sapiens OX=9606 GN=SEPTIN9 PE=1 SV=2	318,05	20	18	18	21,18	36,35	256,43	11	10	10	11,76	23,38	191,22	6	6	6	7,06	12,97
Q9UI10	Translation initiation factor eIF-2B subunit delta OS=Homo sapiens OX=9606 GN=EIF2B4 PE=1 SV=2	72,44	1	1	1	1,41	2,68							61,38	1	1	1	1,41	2,68
Q9UJU6	Drebrin-like protein OS=Homo sapiens OX=9606 GN=DBNL PE=1 SV=1	242,95	9	8	8	16	22,79	145,1	3	3	3	6	6,98	90,29	1	1	1	2	2,33
Q9UJZ1	Stomatin-like protein 2, mitochondrial OS=Homo sapiens OX=9606 GN=STOML2 PE=1 SV=1							83,48	1	1	1	2,27	4,49	73,75	1	1	1	2,27	4,49

Q9UKK3	Protein mono-ADP-ribosyltransferase PARP4 OS=Homo sapiens OX=9606 GN=PARP4 PE=1 SV=3							88,99	1	1	1	0,61	0,58	160,76	4	4	4	2,44	3,02
Q9UKM9	RNA-binding protein Raly OS=Homo sapiens OX=9606 GN=RALY PE=1 SV=1	138,51	5	4	4	10	13,4	168,01	5	4	4	10	14,05	171,34	6	5	5	12,5	17,32
Q9ULV4	Coronin-1C OS=Homo sapiens OX=9606 GN=CORO1C PE=1 SV=1	137,97	4	4	4	6,67	13,71							133,37	3	3	3	5	10,76
Q9UN86	Ras GTPase-activating protein-binding protein 2 OS=Homo sapiens OX=9606 GN=G3BP2 PE=1 SV=2	129,99	4	2	1	3,57	5,19							78,17	1	1	1	1,79	2,7
Q9Y262	Eukaryotic translation initiation factor 3 subunit L OS=Homo sapiens OX=9606 GN=EIF3L PE=1 SV=1	87,3	2	2	2	3,17	5,14							54,56	1	1	1	1,59	1,6
Q9Y3B9	RRP15-like protein OS=Homo sapiens OX=9606 GN=RRP15 PE=1 SV=2							66,44	1	1	1	2	4,61	69,33	1	1	1	2	4,61
Q9Y4B5	Microtubule cross-linking factor 1 OS=Homo sapiens OX=9606 GN=MTCL1 PE=1 SV=5	183,87	5	5	5	1,98	3,1	72,7	1	1	1	0,4	0,79	76,6	1	1	1	0,4	0,68
Q9Y597	BTB/POZ domain-containing protein KCTD3 OS=Homo sapiens OX=9606 GN=KCTD3 PE=1 SV=2	191,57	7	7	6	8,54	10,31							135,75	4	4	3	4,88	6,5
Q9Y5B9	FACT complex subunit SPT16 OS=Homo sapiens OX=9606 GN=SUPT16H PE=1 SV=1	43,48	1	1	1	0,65	1,24							48,9	1	1	1	0,65	1,24
Q9Y678	Coatomer subunit gamma-1 OS=Homo sapiens OX=9606 GN=COPG1 PE=1 SV=1	54,45	2	1	1	1,08	1,72							48,03	1	1	1	1,08	1,72
Q9Y6I4	Ubiquitin carboxyl-terminal hydrolase 3 OS=Homo sapiens OX=9606 GN=USP3 PE=1 SV=2	361,58	28	19	19	29,69	37,12	233,12	9	7	7	10,94	16,73	270,08	18	14	14	21,88	37,88
Q9Y6M1	Insulin-like growth factor 2 mRNA-binding protein 2 OS=Homo sapiens OX=9606 GN=IGF2BP2 PE=1 SV=2	132,51	3	3	3	4,29	6,51	56,51	1	1	1	1,43	2,34	49,81	1	1	1	1,43	2,34
Q9Y6R4	Mitogen-activated protein kinase kinase kinase 4 OS=Homo sapiens OX=9606 GN=MAP3K4 PE=1 SV=2	231,29	9	9	9	4,71	6,97							127,1	3	3	3	1,57	2,18

Table S2. Dynein-associated proteins interacting with LC3s during IAV cell cytoplasm entry identified by mass spectrometry

	Gene name	UniProt ID	Known function
**1**	BIN1	O00499	BIN1 recruits dynein onto endophilin-mediated endocytic carriers.
**2**	CCT5	P48643	CCT proteins have a role in dynein-mediated transport along MTs.
**3**	CDK1	P06493	CDK1 phosphorylates the dynein adaptor NDE1, to control its binding to centrosome during G2 to anaphase.
**4**	DSG1	Q02413	DSG1 binds to DYNLT1, one of the light chains of cytoplasmic dynein motor complex, to promote keratinocyte delamination.
**5**	DST	Q03001	DST interacts with MAP1B in the centrosomal region to maintain MTs acetylation.
**6**	MARK2	Q7KZI7	The cortical dynein–mediated pulling forces are actively counteracted by a MARK2-mediated spindle-centering mechanism to ensure the rotation of the spindle toward its pre-determined final position during cell division.
**7**	MTCL1	Q9Y4B5	MTCL1 is one of the numerous proteins that associates with LC8, a dynein light chain, to promote their stable dimerization.
**8**	PALLD	Q8WX93	PALLD is a MTs-associated protein responsible for spindle orientation.
**9**	PCNT	O95613	PCNT directly interacts with cytoplasmic dynein light intermediate chain to promote the mitotic spindle organization.
**10**	PPP1CC	P36873	PPP1CC interacts with TCTEX1D4, a Tctex1 dynein light chain family member, which is involved in MTs dynamics.
**11**	PPIA	P62937	PPIA directly interacts with the dynein/dynactin motor protein complex to perform a general function related to the binding cargo for retrograde movement along MT.
**12**	RABL6	Q3YEC7	RAB6 family proteins interact with the dynein light chain protein DYNLRB1 to regulate retrograde transport from endosomes via the Golgi to the ER.
**13**	SEPTIN7	Q16181	SEPT7 form a complex with SEPT9 and may have the same function as SEPT9.
**14**	SEPTIN9	Q9UHD8	SEPT9 interacts with both dynein and dynactin, and associates with lysosomes promoting their perinuclear clustering at steady state and during cellular adaptation to stress.
**15**	SEPTIN11	Q9NVA2	SEPT11 is a binding partner of SEPT9, which may have the same function as SEPT9.

Table S3. siRNAs used in this study

	Targeted protein	Company and catalogue number
**1**	HDAC6	QIAGEN, SI03058706
**2**	ATG7	Dharmacon, J-020112-08-0002
**3**	ATG13	Dharmacon, J-020765-12-0020
**4**	Scramble	Dharmacon, D-001810-01-05
**5**	DYNC1I1	Dharmacon, L-019799-00-0005
**6**	LC3A	Dharmacon, L-013579-00-0005
**7**	LC3B	Dharmacon, L-012846-00-0005
**8**	LC3C	Dharmacon, L-032399-01-0005
**9**	BIN1	Dharmacon, L-008246-00-0005
**10**	CCT5	Dharmacon, L-012797-00-0005
**11**	CDK1	Dharmacon, L-003224-00-0005
**12**	DSG1	Dharmacon, L-011644-00-0005
**13**	DST	Dharmacon, L-011596-00-0005
**14**	MARK2	Dharmacon, L-004260-00-0005
**15**	MTCL1	Dharmacon, L-023376-01-0005
**16**	PALLD	Dharmacon, L-016891-00-0005
**17**	PCNT	Dharmacon, L-012172-00-0005
**18**	PPP1CC	Dharmacon, L-006827-00-0005
**19**	PPIA	Dharmacon, L-004979-04-0005
**20**	RABL6	Dharmacon, L-031749-01-0005
**21**	SEPTIN7	Dharmacon, L-011607-00-0005
**22**	SEPTIN9	Dharmacon, L-006373-00-0005
**23**	SEPTIN11	Dharmacon, L-020249-00-0005
**24**	ATP6V1A	Dharmacon, L-017590-01-0005
**25**	ATP6V1B2	Dharmacon, L-011589-01-0005
